# A large aberrant stem ichthyosauriform indicating early rise and demise of ichthyosauromorphs in the wake of the end-Permian extinction

**DOI:** 10.1038/srep26232

**Published:** 2016-05-23

**Authors:** Da-Yong Jiang, Ryosuke Motani, Jian-Dong Huang, Andrea Tintori, Yuan-Chao Hu, Olivier Rieppel, Nicholas C. Fraser, Cheng Ji, Neil P. Kelley, Wan-Lu Fu, Rong Zhang

**Affiliations:** 1 Laboratory of Orogenic Belt and Crustal Evolution, Ministry of Education; Department of Geology and Geological Museum, Peking University, Yiheyuan Street. 5, Beijing 100871, People’s Republic of China; 2 Department of Earth and Planetary Sciences, University of California, Davis, One Shields Avenue, Davis, California 95616, United States of America; 3 Department of Research, Anhui Geological Museum, Jiahe Road 999, Hefei, Anhui 230031, People’s Republic of China; 4 Dipartimento di Scienze della Terra, Università degli Studi di Milano, Via Mangiagalli 34-20133 Milano, Italy; 5 Center of Integrative Research, The Field Museum, Chicago, IL 60605-2496, United States of America; 6 National Museums Scotland, Chambers Street, Edinburgh EH1 1JF, United Kingdom; 7 Key Laboratory of Economic Stratigraphy and Palaeogeography, Nanjing Institute of Geology and Palaeontology, Chinese Academy of Sciences, 39 East Beijing Road, Nanjing 210008, People’s Republic of China; 8 Smithsonian Institution, National Museum of Natural History, Washington, DC 20560-0121, United States of America

## Abstract

Contrary to the fast radiation of most metazoans after the end-Permian mass extinction, it is believed that early marine reptiles evolved slowly during the same time interval. However, emerging discoveries of Early Triassic marine reptiles are questioning this traditional view. Here we present an aberrant basal ichthyosauriform with a hitherto unknown body design that suggests a fast radiation of early marine reptiles. The new species is larger than coeval marine reptiles and has an extremely small head and a long tail without a fluke. Its heavily-built body bears flattened and overlapping gastral elements reminiscent of hupehsuchians. A phylogenetic analysis places the new species at the base of ichthyosauriforms, as the sister taxon of *Cartorhynchus* with which it shares a short snout with rostrally extended nasals. It now appears that ichthyosauriforms evolved rapidly within the first one million years of their evolution, in the Spathian (Early Triassic), and their true diversity has yet to be fully uncovered. Early ichthyosauromorphs quickly became extinct near the Early-Middle Triassic boundary, during the last large environmental perturbation after the end-Permian extinction involving redox fluctuations, sea level changes and volcanism. Marine reptile faunas shifted from ichthyosauromorph-dominated to sauropterygian-dominated composition after the perturbation.

Ichthyopterygia comprise a group of Mesozoic marine reptiles that are commonly referred to as ichthyosaurs. It is best known for the evolution of fish-shaped body profiles among its derived members that are represented by abundant well-preserved fossils^1^. In contrast, the earliest evolution of the group still remains elusive, although recent studies recognize that they form the clade Ichthyosauromorpha with Hupehsuchia, a group of Early Triassic marine reptiles that inhabited a part of the South China block that later became western Hubei Province, China^2^,^3^,^4^. However, there is a wide anatomical gap between Hupehsuchia and Ichthyopterygia and only one species is so-far known to fill the gap, namely the basal ichthyosauriform *Cartorhynchus lenticarpus*^2^. Although the discovery of *Cartorhynchus* largely advanced our understanding of the early evolution of ichthyosaurs, additional information is clearly missing. Here we report a second species of basal ichthyosauriform that differs significantly from *Cartorhynchus* in its anatomical features.

## Materials and Methods

The main material for the study is the holotype of the new taxon proposed below. We also examined the holotype of *Cartorhynchus lenticarpus* (Anhui Geological Museum (AGB) 6257)^2^ and described specimens of *Chaohusaurus* (Institute of Vertebrate Paleontology and Paleoanthropology (IVPP) V4001, AGB P45-H85-25, AGB P45-H85-20, AGB MT10010)^5^,^6^,^7^, *Hupehsuchus* (IVPP V3232, Wuhan Center of China Geological Survey (WGSC) V26000)^8^,^9^, *Eohupehsuchus* (WGSC 26003)^10^, *Nanchangosaurus* (Geological Museum of China (GMC) V646, WGSC 26006)^3^,^11^, and *Parahupehsuchus* (WGSC 26005)^12^ for comparison.

Phylogenetic analyses are based on the most recent osteological datasets for diapsid relationships and ichthyosauromorph phylogeny^2^,^13^. The new taxon was added to these matrices, together with five new characters for the diapsid matrix, and 12 for the ichthyosauromorph matrix. The diapsid matrix was analyzed in two ways following a published procedure to test for the influence of aquatic adaptations^3^. See Supplementary Information for the details of added characters and coding. The ichthyosauromorph matrix was analyzed with and without poorly known taxa, namely *Parvinatator*, *Acamptonectes*, *Maiaspondylus*, *Arthropterygius*, *Malawania*, *Chacaicosaurus*, *Mollesaurus*, *Leninia*, *Thalattoarchon*, *Palvennia*, and *Sisteronia*, following a published procedure^13^.

Phylogenetic analyses were conducted using a heuristic search option of PAUP*4b10 (hold = 50, nreps = 100, addseq = random, swap = tbr). All multistate characters were treated as unordered. The shortness of the most parsimonious solutions suggested by heuristic searches were compared to the outcome of new technology searches of TNT 1.1. Bremer support values were estimated using TNT 1.1. For comparative purpose, we also conducted Bayesian phylogenetic analyses (Supplementary Method).

Systematc Paleontology

Reptilia Laurenti, 1768.

Diapsida Osborn, 1903.

Ichthyosauromorpha Motani *et al.*, 2015.

Ichthyosauriformes Motani *et al.*, 2015.

Nasorostra nov.

### Etymology

Nasus (Latin nose) and rostrum (Latin beak), referring to the snout with the nasal bone reaching the tip.

### Diagnosis

Rostrally elongate nasal reaching snout tip; preorbital and postorbital skull lengths sub-equal; frontal without distinctive posterolateral process; deep posterior mandible with slanting end and low jaw joint; ribcage deepest near shoulder; scapular blade wider distally than proximally.

*Sclerocormus parviceps* gen. et sp. nov.

### Etymology

Genus name from Greek skleros and kormos, ‘stiff trunk’; species name from Latin parvus and caput, ‘small skull’.

### Holotype

**Figure 1:**
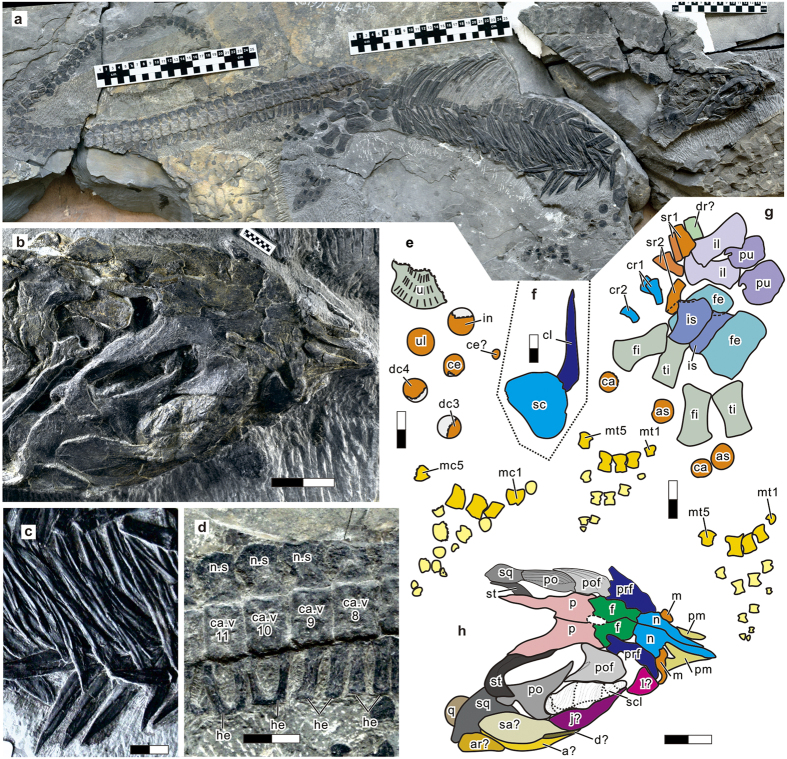
The holotype of *Sclerocormus parviceps* gen. et sp. nov. (**a**) Whole specimen. (**b**) Skull. (**c**) Close-up of gastral basket. (**d**) Close-up of U-shaped haemal arches. (**e**) Right forelimb. (**f**) Shoulder elements. (**g**) Pelvic girdle and hind limb. (**h**) Skull elements. Abbreviations: a, angular; ar, articular; as, astragalus; ca, calcaneum; car, caudal rib; ca.v, caudal vertebra; ce, centralia; cl, clavicle; d, dentary; dc, distal carpal; f, frontal; fe, femur; fi, fibula; he, hemal arch; il, ilium; in, intermedium; is, ischium; j, jugal, l, lacrimal; m, maxilla; mc, metacarpal; mt, metatarsal; n, nasal; p, parietal; pm, premaxilla; po, postorbital; pof, postfrontal; prf, prefrontal; pu, pubis; q, quadrate; sa, surangular; sc, scapula; scl, scleral ossicles; sq, squamosal; sr, sacral rib; st, supratemporal; ti, tibia; u, ulna; ul, ulnare. Scale unit in (**a**) is 1 cm, other scale bars are 2 cm.

(Figure 1) Anhui Geological Museum AGB6265.

### Diagnosis

Skull very short, occupying 6.25% of total length; tail long, about 58% of total length; body trunk short and deep; preorbital snout constricted and extremely short, about 30% of skull length; orbit large, more than one third of skull length; pineal foramen large, located at fronto-parietal suture; nasal large; ribs flattened, with blunt distal ends; gastralia robust, forming tight ventral basket; dorsal neural spines tall and vertical, with craniad and caudad flanges sandwiching the thickened shaft; caudal neural spines short with rounded top; femur straight, without shaft constriction.

### Locality and horizon

**Figure 2:**
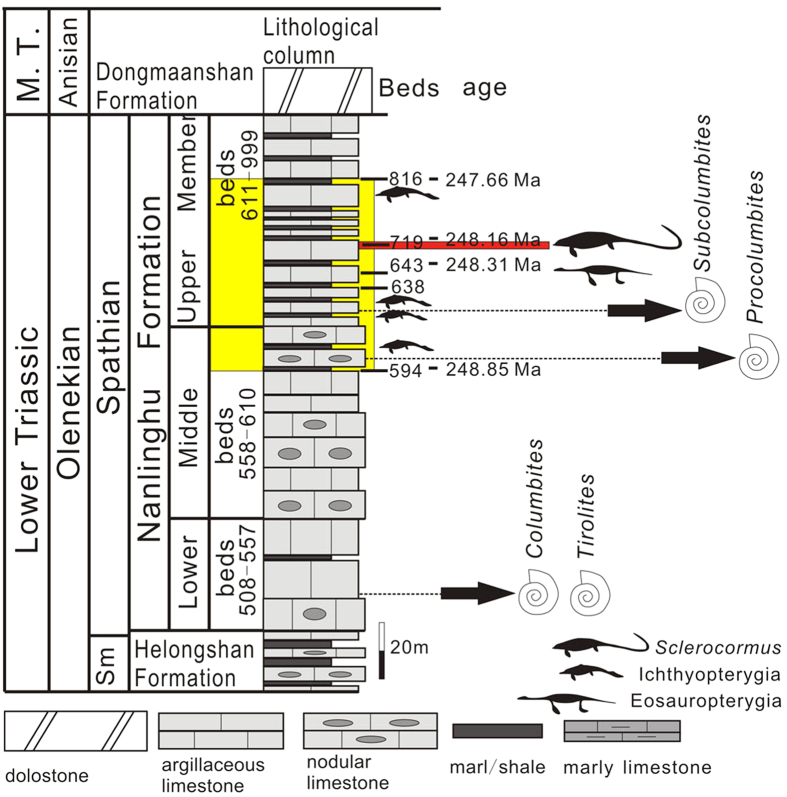
Stratigraphic horizon of *Sclerocormus parviceps* gen. et sp. nov. The specimen is from bed 719 (red) within the Upper Member of the Nanlinghu Formation. Stratigraphic ranges of Early Triassic marine reptiles are marked by yellow. Abbreviations: M.T., Middle Triassic; Sm, Smithian.

From the first level of Majiashan Quarry, Chaohu, Anhui Province, China. Bed 719, about 27 m above the bottom of the Upper Member of the Nanlinghu Formation, within the ammonite *Subcolumbites* zone, Spathian, Olenekian, Lower Triassic (Fig. 2).

## Description

**Figure 3:**
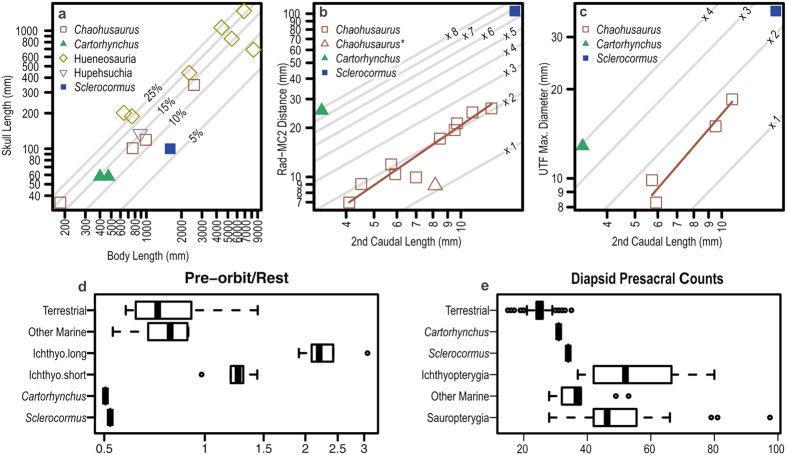
Quantitative comparisons of selected features. (**a**) Skull proportions relative to the body length in Ichthyosauromorpha. (**b**) Carpus length relative to body size. The carpus length is measured as the distance between the radius and the second metacarpal, whereas length of the second caudal vertebra is used as the proxy for body size, following a previous work^41^. (**c**) Maximum diameter of the upper temporal fenestra (UTF) relative to a body size proxy. (**d**) Snout proportions relative to the post-snout skull length. The part of the skull anterior to the orbit is considered the snout. (**e**) Presacral count of *Sclerocormus* compared to other diapsids, based on published data^42^. In (**a**), gray lines represent isoclines for the proportion of the skull length relative to the body length. Symbols with white infill represent estimated positions of newborns, which tend to have a large skull for the body. Two points for *Cartorhynchus* represent estimated body lengths based on *Chaohusaurus* and *Sclerocormus* tail/body proportions, respectively. *Sclerocormus* has an unusually small skull.

The skeleton measures 159.9 cm along the vertebral column, of which 92.1 cm comprises the tail. There are about 34 presacral, two sacral, and at least 67 caudal vertebrae. Given the incomplete preservation of the vertebral column, the presacral count is based on the number of cervical and dorsal neural spines for the anterior half, and that of ribs for the posterior half, accounting for the presence of right and left ribs. There is a wide crack in the mid-dorsal region, potentially lowering the accuracy of the vertebral count. However, given that some ribs crossover the crack, the count is more accurate than it may appear, although it may be off the true count by plus or minus 1. These counts suggest that the trunk is short compared to ichthyopterygians (Fig. 3e), most of which have 40 to 80 presacral vertebrae, with an exception of *Chaohusaurus* with 36. There seem to be five cervicals but the exact count is uncertain because the anterior dorsal region was damaged (Fig. 1a) during excavation.

The holotype skull of *Sclerocormus* has been dorso-laterally compressed leading to the displacement and plastic deformation of some elements and fracturing of others. It is a particularly small skull, measuring only 6.25% of the total body length (Figs 1a–d and 3a). In basal ichthyosauromorphs, this ratio ranges from about 12% in *Chaohusaurus* to 15% in *Hupehsuchus* (Fig. 3a). The snout of *Sclerocormus* is short (Fig. 3d), edentulous and much narrower than the skull roof, as in *Cartorhynchus*. This would allow syringe-like pressure concentration common among suction feeders^14^. The nasal is large, extending anteriorly to the tip of the snout as in *Cartorhynchus* but unlike the condition in most reptiles. The external naris is preserved as a small slit (due to compaction during preservation) and closely located to the orbit, which is large and contains extensive scleral plates as in most ichthyosauriforms. These plates have been finely fractured from compression unlike the surrounding bones, yet they still retain clear plate boundaries that allow recognition of at least five different plates. Their surfaces lack the long striations seen in other cranial bones. The frontal does not enter the margin of the orbit, unlike *Cartorhynchus*. The pineal foramen measures 9.1 mm in length, and is located between the parietals and frontals, near the anterior margin of the large upper temporal fenestrae. As with other ichthyosauriforms, the pineal foramen lies between the orbits, whereas it is located behind the orbits in most other reptiles. The antero-posterior length of this fenestra is about 36.1 mm, which is large for the body size compared to that of *Chaohusaurus* (Fig. 3c). The large size of the upper temporal fenestrae may indicate a strong biting force^15^,^16^. However, no teeth appear to be present in *Sclerocormus*.

The limb elements of *Sclerocormus* are widely spaced as in *Cartorhynchus* but slightly better ossified. The mesopodia are unusually long (Fig. 3b) and bear sparse, round elements. The forelimb is strongly bent as in *Cartorhynchus*, although this may reflect postmortem displacement of the distal part, unlike in the holotype of the latter genus. There are three rows of carpals, probably including two centralia. Five digits, the longest of which comprises up to four phalanges, are recognized in both fore- and hind limbs. The femur is straight, lacking a marked distal expansion, as in *Cartorhynchus* but unlike in other basal ichthyosauromorphs. Apart from the astragalus and calcaneum, no other tarsal ossifications are present. Despite the poor ossification of limb bones, the type specimen of *Sclerocormus* is most likely mature given the advanced ossification of the rest of the body, as in *Cartorhynchus* and *Chaohusaurus*. Delay in limb ossification is a common feature among basal ichthyosauriforms.

The short trunk is heavily built. The ribs, which are flattened and broad, are single-headed and articulate almost exclusively with the vertebral centra as in all ichthyosauriforms, although rib articulation may minimally extend to the neural arch in the anterior trunk. The ribcage shallows behind the shoulder with a constant slope, forming a nearly linear ventral margin as in *Cartorhynchus* but unlike in other ichthyosauromorphs where the margin is curved. The trunk of *Sclerocormus* is covered ventrally by an extensive gastral rib basket (Fig. 1a,h). The gastral elements are in two parallel series per side, of which the lateral series is unique to ichthyosauriforms among Ichthyosauromorpha. The medial series elements are flat triangles that overlap each other, with the caudad element outlying its craniad neighbour. The flatness and overlapping pattern are shared with Hupehsuchia^4^,^12^. The absence of symmetrical median gastral elements is unique to *Sclerocormus* and *Cartorhynchus* among basal ichthyosauromorphs. Dermal ossicles are present in the cervical region but not on the neural spines, unlike in hupehsuchians. They are small rounded elements of various diameters between 2.0–5.0 mm. Each ossicle resembles the pelvic ossicles of saurosphargids^17^, although the locations differ.

The cervical and anterior dorsal neural spines are vertical, unlike those of ichthyopterygians, yet they are antero-posteriorly broadened to leave almost no interspinal space as in Hupehsuchia, but unlike in basal Ichthyopterygia where these neural spines are posteriorly inclined with spaces in between them. Despite the expanded width, the neural spines are at least as tall as they are wide, or taller. Strangely, there are craniad and caudad flanges sandwiching the thickened shaft of each neural spine. This peculiar feature is also seen in one specimen of *Hupehsuchus*^12^, and among Ichthyopterygia, Merriam^18^ described the neural spine of *Shastasaurus* and *Californosaurus* to have a thickened central shaft with craniad and caudad ridges or flanges. Also, a previous study noticed weak craniad and caudad ridges in the vertical neural spines of *Hovasaurus*, a freshwater reptile from the Late Permian of Madagascar^19^. However, the features do not appear homologous according to our phylogenetic analysis. The caudal neural spines are lower than wide and their tops are rounded in side view, yet they also possess craniad and caudad flanges and a central shaft (Fig. 1j). The haemal arches show a unique morphology. The first eight pairs are not fused distally right to left. The ninth and later pairs, however, are fused distally and appear U-shaped in cranial view, not V- or Y-shaped as in typical diapsid haemal arches (Fig. 1a,j). The modification of haemal arches differs from those of placodonts^20^ and *Atopodentatus*^21^ involving antero-posterior lengthening rather than lateral widening, or of drepanosaurs, with distal forking and secondary fusion^22^.

## Results

**Figure 4:**
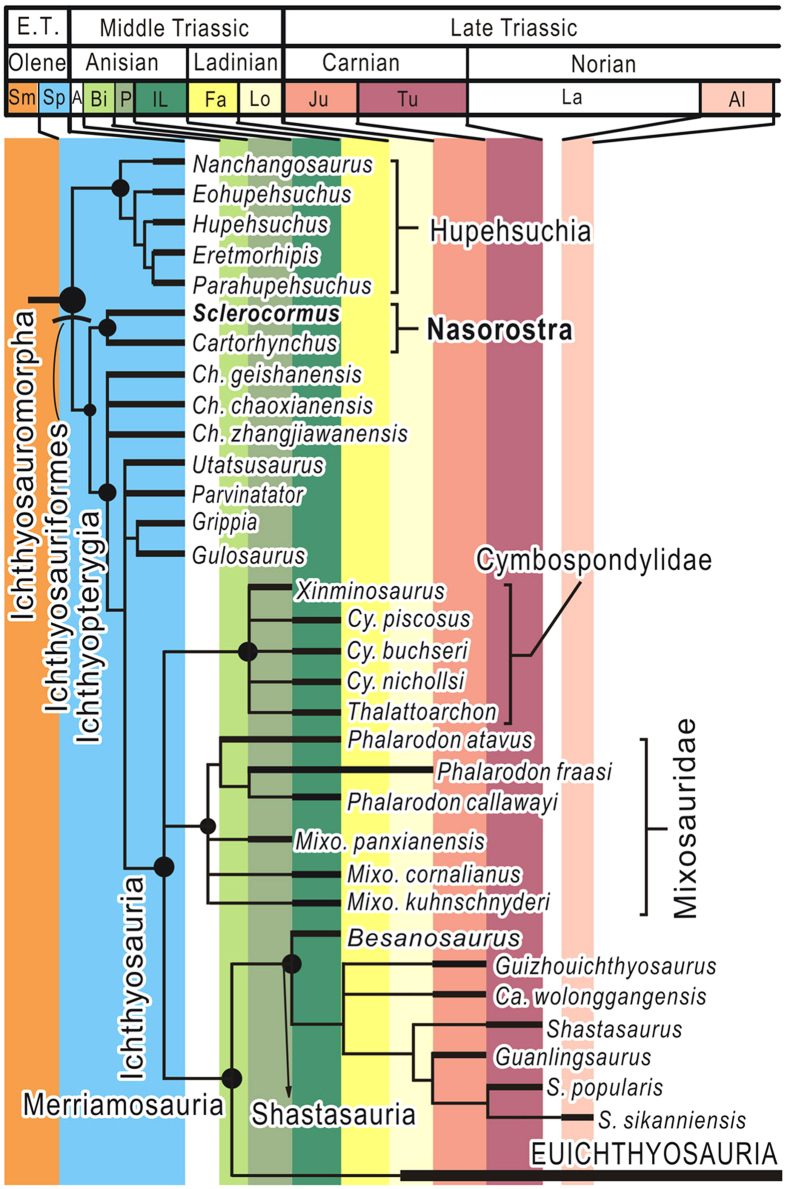
Phylogenetic position of *Sclerocormus*. Analyses are based on the most recent phylogenetic datasets for marine reptile relationships and ichthyopterygian phylogeny^13^ (Supplementary Information). The tree was abbreviated from the more complete topology (Extended Data Figure 1). Phylogenetic hypotheses of *Sclerocormus* among Diapsida are shown in Extended Data Figure 2. Abbreviations: A, Aegean; Al, Alaunian; Bi, Bithynian; Ca, *Callawayia*; Ch, *Chaohusaurus*; Cy, *Cymbospondylus*; E.T., Early Triassic; Fa, Fassanian; Il, Illyrian; Ju, Julian; La, Lacian; Lo, Longobardian; Mixo, *Mixosaurus*; Olene, Olenekian; P, Pelsonian; S, *Shonisaurus*; Sm, Smithian; Sp, Spathian; Tu, Tuvalian.

Heuristic searches in PAUP* 4b10 found the following numbers of most parsimonious trees from three data matrices that were analyzed: 800 (Tree Length (TL) = 640, Consistency Index (CI) = 0.369, Retention Index (RI) = 0.781) for the data matrix for ichthyosauromorphs when poorly-known taxa were removed; more than 100000 (TL = 674, CI = 0.350, RI = 0.778) for the same data matrix with all taxa included; 2 (TL = 822, CI = 0.310, RI = 0.589) for the diapsid matrix without special treatment of aquatic adaptations; and 12 (TL = 857, CI = 0.307, RI = 0.614) for the diapsid matrix where aquatic adaptations were coded as ambiguous. The strict consensus trees are given in Fig. 4 and Extended Data Figs 1 and 2, with Bremer support values that were estimated using TNT 1.1. In all three cases, a sister-group relationship between *Sclerocormus* and *Cartorhynchus*, forming the clade Nasorostra (Fig. 4), was recognized, with high Bremer support value of 5 to 7. For comparisons, results from Bayesian phylogenetic analyses are given in Supplementary Extended Data Figs 3 and 4. In all cases, *Sclerocormus* formed the clade Nasorostra with *Cartorhynchus*, as the sister group of Ichthyopterygia.

The monophyly of the genus *Chaohusaurus* became ambiguous (Fig. 4). The three species in this genus formed a clade in 60% of the equally most parsimonious trees with or without the poorly-known taxa included in the analysis. This is partly because those characters that were considered to be synapomorphies of the three species turned out to be plesiomorphies of ichthyosauriformes after the recent discoveries of *Cartorhynchus* and *Sclerocormus*.

## Discussion

Despite many similarities mentioned above, a list of features establishes that *Sclerocormus* is not a large *Cartorhynchus*. The presacral count of *Sclerocormus* (34) is closer to that of *Chaohusaurus* (36) than of *Cartorhynchus* (31), and changes in presacral count are unlikely during post-embryonic growth. The pineal foramen is located between the parietals in *Cartorhynchus*, while it lies at the suture between the parietal and frontal in *Sclerocormus*. The frontal participates in the margin of the orbit in *Cartorhynchus*, but not in *Sclerocormus*. The neural spines of *Cartorhynchus* are inclined, narrow and lack flanges, unlike the vertical and broadly flanged neural spines of *Sclerocormus*. No dermal ossicle is known in *Cartorhynchus*. Finally, the gastralia of *Cartorhynchus* comprise a series of narrow rods per side, unlike the flattened elements forming a robust basket in *Sclerocormus*.

Nasorostrans are known only from the Spathian of Majiashan. However, given the major differences between *Cartorhynchus* and *Sclerocormus* in body size, shape, and stratigraphic horizon, it is likely that there is a hidden diversity of this group still to be uncovered. For example, there is an enigmatic marine reptile from the Spathian *Grippia* level of Spitsbergen (SVT 203)^23^—its pubis, ischium, and femur resemble those of *Sclerocormus*, although there are differences in the tibiae and phalanges. The species diversity of Early Triassic ichthyosauromorphs has almost doubled over the last three years, to at least 15. Given that the fossil record of Ichthyosauromorpha is unlikely to extend back to the Smithian (see discussions in the supplementary information of a previous work^9^), their evolution likely proceeded very rapidly in the early-mid Spathian. The diversification probably continued until the late Spathian—it was previously suggested that the body size of Ichthyopterygia became large by the *Subcolumbites* zone of the late Spathian, potentially suggesting a burst of evolution^24^, although the suggested body size (~10 m) may be questionable given that it was based on a single unusually-shaped bone, and by assuming isometric scaling which may not be realistic. The bone, which was tentatively identified as an ichthyopterygian humerus^24^, somewhat resembled the type-2 humerus^25^ of Ichthyopterygia in having a posterior displacement of the putative deltopectoral crest. However, the displacement is too extensive, and the anterior flange too thick and expanded proximally for an ichthyopterygian humerus. The bone may also be a coracoid or one of other girdle bones of either Ichthyopterygia or other marine reptiles, in which case the estimated body size may shrink substantially. A concrete conclusion on its identity is difficult to draw at this point because of the isolated nature of the specimen. However, given that the bone likely belongs to a marine reptile, it does indicate at least some degree of body size enlargement among marine reptiles by the *Subcolumbites* zone of the Spathian.

**Figure 5:**
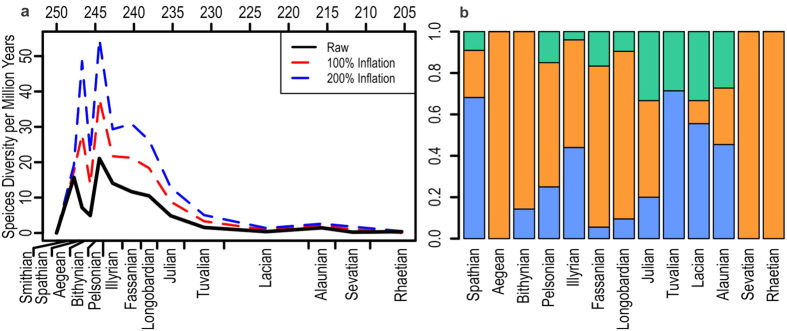
Taxonomic diversity of Triassic marine reptiles. (**a**) Species diversity per million years. (**b**) Clade proportions through the Triassic. Line colors in (**a**) black, raw data; red, data with inflated stratigraphic ranges where half of the species are assumed to have their records missing from the substages before and after the actual record; blue, data with extremely inflated stratigraphic ranges where all species are assumed to be missing their records from the substages before and after the actual record. Blue line is unlikely given that 94.4% of about 150 species examined are only known from one substage, suggesting that species turnover was fast among marine reptiles. Fill colors in (**b**) blue, Ichthyosauromorpha; orange, Sauropterygia + Saurosphargidae; green, others. See Supplementary Tables S2 and S3 for data.

It has been considered that early Mesozoic marine reptiles evolved slowly in the Early Triassic after the end-Permian mass extinction^26^, contrary to the fast radiation of most metazoans during the same time interval^27^,^28^. The present and other recent discoveries of Early Triassic marine reptiles^2^,^4^,^10^,^12^,^29^,^30^ indicate that the diversification of Triassic marine reptiles was not a single phase of unbroken increase in diversity. There were at least two waves of marine reptile diversification, separated by a taxonomic bottleneck near the Early-Middle Triassic boundary (Fig. 5a). The first, a Spathian diversification, is principally attributable to Ichthyosauromorpha while the second radiation, following the bottleneck, was driven by the Sauropterygia-Saurosphargidae clade (Fig. 5b). The first occurred over about one million years, and the second over three to five million years. There is a faunal turnover from ichthyosauromorph-dominated to sauropterygian-saurosphargid-dominated composition at this bottleneck (Fig. 5b).

The timing of the taxonomic bottleneck corresponds to the last large environmental perturbation following the end-Permian mass extinction, associated with a positive carbon isotope excursion near the Early-Middle Triassic boundary^31^,^32^. The excursion reportedly reflects suboxia in deep and shallow seas^32^, and coincides with a major sea-level change^33^ and active volcanism^34^,^35^,^36^. These environmental changes reportedly disrupted the evolution of ammonoids^37^,^38^, and reduced the diversity of conodonts^39^. It seems that they also disturbed the early evolution of marine reptiles by removing many of the “early starters”. Ichthyosauromorphs occupied both demersal (pachyostotic taxa, such as hupehsuchians^9^ and nasorostrans^2^) and pelagic (most ichthyopterygians) niches and contributed to a high diversity of feeding function^9^ in the Spathian but never regained such a high functional diversity after the environmental perturbation, with the loss of hupehsuchians and nasorostrans.

Previous authors found that a marine ecosystem, incorporating large marine reptiles with a size of Killer Whales as macro predators, was already present by the mid-Middle Triassic^40^ (Illyrian, the last substage of the Anisian), about three to five million years after the faunal turnover and environmental perturbation discussed here. Given that the perturbation must have delayed the formation of the ecosystem to a large extent, the construction of the marine ecosystem within the Anisian likely proceeded faster than with a single stretch of unbroken radiation over the Spathian and Anisian (five to seven million years), which was implicitly assumed previously. Also, it is now unclear how many times enlargement of body size in top predators occurred before the late Anisian marine ecosystem was formed because there may have been a decrease of the top-predator size after the perturbation, partly reversing what was achieved during the Spathian. Improvement of the fossil record from the earliest Anisian would be necessary to clarify this question.

## Additional Information

**How to cite this article**: Jiang, D.-Y. *et al.* A large aberrant stem ichthyosauriform indicating early rise and demise of ichthyosauromorphs in the wake of the end-Permian extinction. *Sci. Rep.* **6**, 26232; doi: 10.1038/srep26232 (2016).

## Supplementary Material

Supplementary Information
