# Supplementary material for: A large aberrant stem ichthyosauriform indicating early rise and demise of ichthyosauromorphs in the wake of the end-Permian extinction
Source: Sci Rep. 2016 May 23;6:26232. doi: 10.1038/srep26232 (PMC4876504; doi:10.1038/srep26232)
Supplement: Supplementary Information [file srep26232-s1.pdf]

Supplementary Information for

A large aberrant stem ichthyosauriform indicating early rise and demise of ichthyosauromorphs in the wake of the end-Permian extinction

DA-YONG JIANG, RYOSUKE MOTANI, JIAN-DONG HUANG, ANDREA TINTORI, YUAN-CHAO HU, OLIVIER RIEPPEL, NICHOLAS C. FRASER, CHENG JI, NEIL P. KELLEY, WAN-LU FU, and RONG ZHANG

Table of Contents

Supplementary Extended Data Figures ········ 3

Extended Data Figure 1 ········ 3

Extended Data Figure 2 ········ 4

Extended Data Figure 3 ········ 5

Extended Data Figure 4 ········ 6

Supplementary Data S1 ········ 7

Character Coding Revision ········ 7

Character Number Correspondence ········ 9

Character Description ········ 10

Character Matrix ········ 17

Supplementary Data S2 ········ 26

New Added Characters ········ 26

Matrix without aquatic adaptations ········ 26

Matrix with aquatic adaptations ········ 30

Supplementary Table S1 ········ 35

Supplementary Table S2 ········ 38

Supplementary Table S3 ········ 40

Supplementary Methods ········ **47**

Supplementary NEX File Data1 ········ **48**

Supplementary NEX File Data2 ········ **78**

Supplementary NEX File Data3 ········ **83**

Supplementary Extended Data Figures

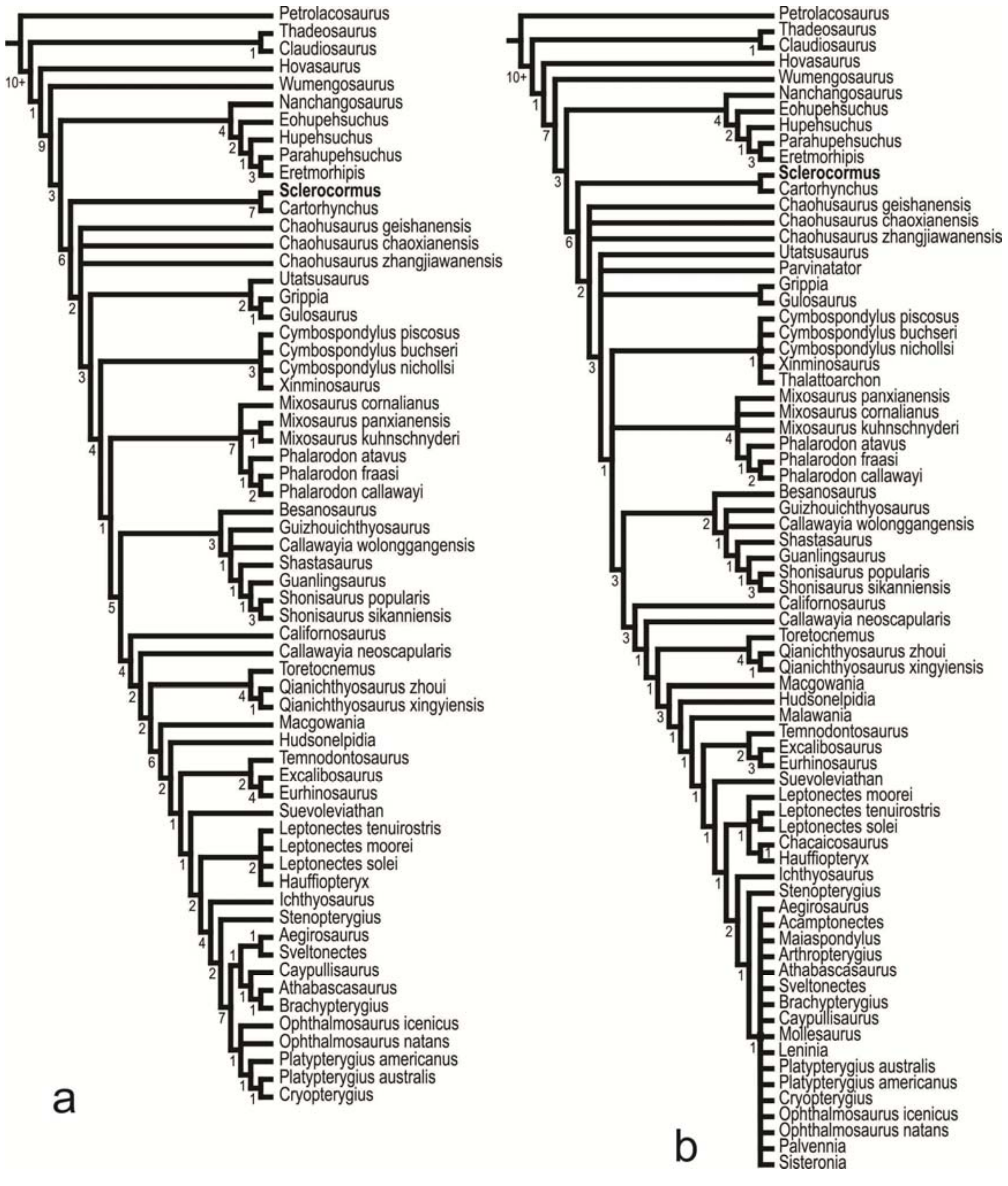

Extended Data Figure 1. Phylogenetic hypothesis of Ichthyosauromorpha based on parsimony analyses in PAUP* 4b10. **a**, When poorly known taxa are removed according to a previous work[1]. Strict consensus of 800 most parsimonious trees (TL=640, CI=0.369, RI=0.781) obtained by a heuristic search in PAUP* 4b10 (hold=10, nreps=100, addseq=random, swap=tbr). Taxa removed are: *Parvinatator*, *Acamptonectes*, *Maiaspondylus*, *Arthropterygius*, *Malawania*, *Chacaicosaurus*, *Mollesaurus*, *Leninia*, *Thalattoarchon*, *Palvennia*, and *Sisteronia*.. **b**, When all taxa in the data are included. Strict consensus of 100000 equally most parsimonious trees (TL=674, CI=0.350, RI=0.778). Original tree of Fig. 4. For both a and b, numbers associated with clades are Bremer support values calculated in TNT 1.1. Based on the data described in Supplementary Information.

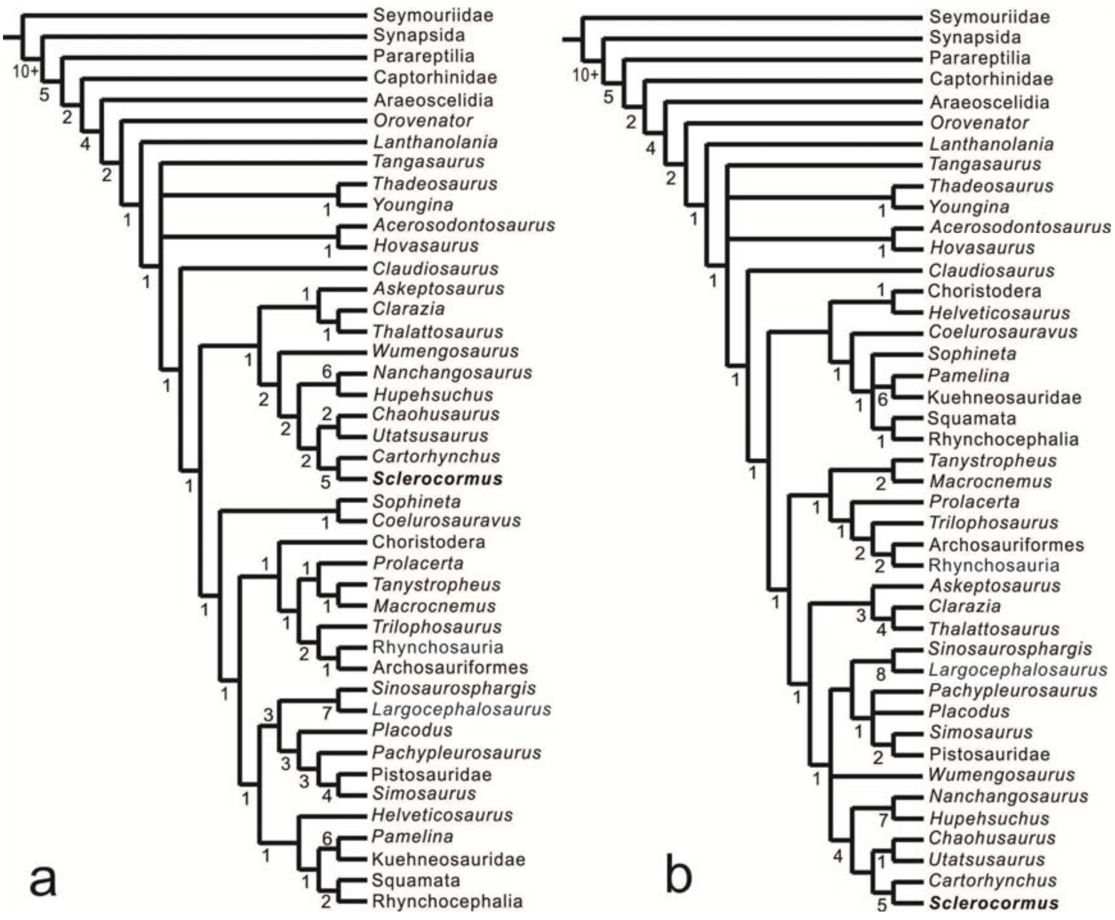

Extended Data Figure 2. Phylogenetic hypotheses of *Sclerocormus* among Diapsida. Based on parsimony analyses in PAUP* 4b10. **a**, When aquatic adaptations are recoded as ambiguous. Strict consensus of 2 most parsimonious trees (TL=822, CI=0.310, RI=0.589) obtained by a heuristic search in PAUP* 4b10 (hold=10, nreps=100, addseq=random, swap=tbr). **b**, When aquatic adaptations are coded normally. Strict consensus of 12 most parsimonious trees (TL=857, CI=0.307, RI=0.614) obtained by a heuristic search in PAUP* 4b10 (hold=10, nreps=100, addseq=random, swap=tbr). For both a and b, numbers associated with clades are Bremer support values calculated in TNT 1.1. Based on the data described in Supplementary Information.

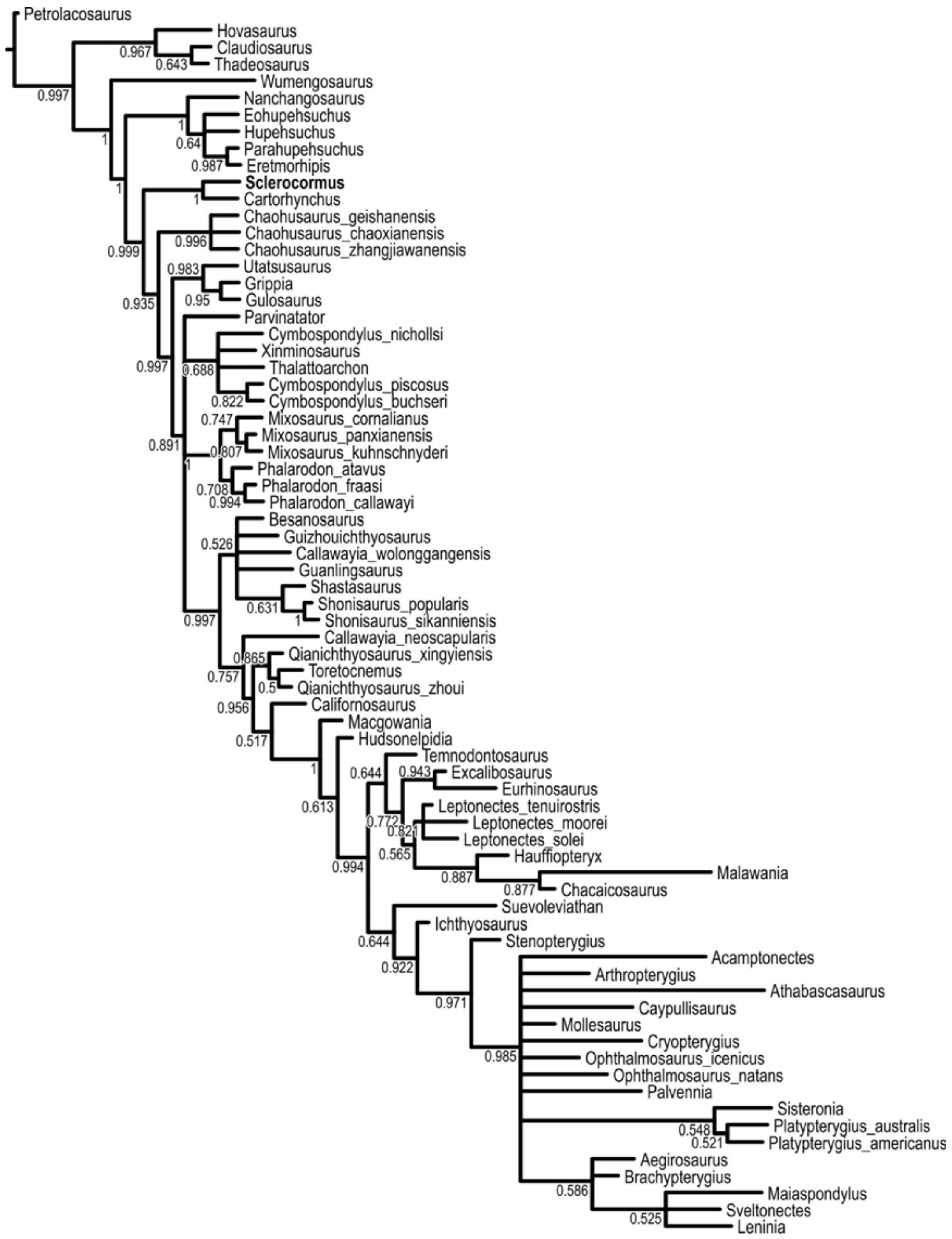

Extended Data Figure 3. Phylogenetic hypotheses of *Sclerocormus* among Ichthyosauromorpha based on Bayesian phylogenetic inference in MrBayes 3.2.6. The values associated with nodes are the mean posterior probabilities. See Supplementary Method for the settings used.

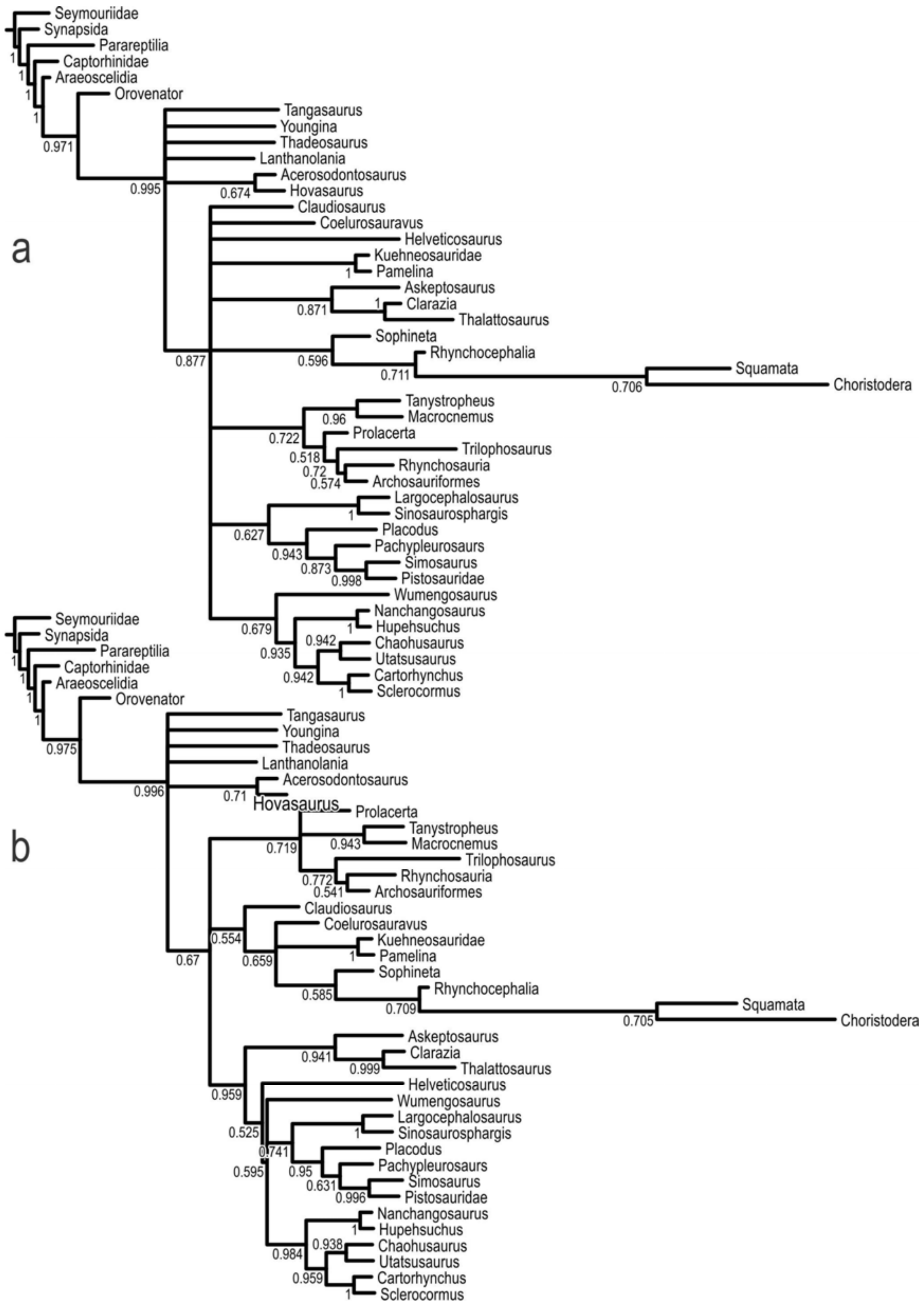

Extended Data Figure 4. Phylogenetic hypotheses of *Sclerocormus* among Diapsida based on Bayesian phylogenetic inference in MrBayes 3.2.6. **a**, When aquatic adaptations are recoded as ambiguous. **b**, When aquatic adaptations are coded normally. The values associated with nodes are the mean posterior probabilities. See Supplementary for the settings used.

Supplementary Data S1. Character list and matrix for the internal phylogeny of Ichthyosauriformes

Coding by Ji et al. (2016) [1] was adopted for all taxa except *Hupehsuchus*, *Chaohusaurus*, and *Cartorhynchus*, which were coded according to Motani et al. (2015) [2]. Note that *Chaohusaurus* was divided into three species by Motani et al. (2015). *Wumengosaurus* was added in case it may be closely related to Ichthyosauromorpha, as suggested by Chen et al. (2014) [3]. Character coding schemes differ between Motani et al. (2015) and Ji et al. (in press) for characters 2, 18, 48, 61, 86, 94, 107,126, 138, 147, and 152. Appropriate adjustments were made to those taxa for which coding from Motani et al. (2015) was employed to adjust for the scheme employed by Ji et al. (in press). New characters 184-195 were added. Character 94 of Motani et al. (2015) was removed following Ji et al. (2016).

Following Ji et al. (2016), the taxa below were considered poorly-known given that only a third of the characters or less were coded: *Parvinatator, Acamptonectes, Maiaspondylus, Arthropterygius, Malawania, Chacaicosaurus, Mollesaurus, Leninia, Thalattoarchon, Palvennia*, and *Sisteronia*. We therefore presented phylogenetic hypotheses with and without these taxa in Extended Data Figure 1b and a, respectively.

1. Ji C., JIang D.-y., Motani R., Rieppel O., Hao W.-c., Sun Z.-y. 2016 Phylogeny of Ichthyopterygia incorporating the recent discoveries from South China. *J Vertbr Paleontol* **36**(1), e1025956, doi: 10.1080/02724634.2015.1025956.
2. Motani R., Jiang D.-y., Chen G.-b., Tintori A., Rieppel O., Ji C., Huang J.-d. 2015 A basal ichthyosauriform with a short snout from the Lower Triassic of China. *Nature* **517**, 485-488.
3. Chen X., Motani R., Cheng L., Jiang D., Rieppel O. 2014 The enigmatic marine reptile *Nanchangosaurus* from the Lower Triassic of Hubei, China and the phylogenetic affinity of Hupehsuchia. *PLoS One* **9**(7), e102361. (doi:10.1371/journal.pone.0102361).

Character Coding Revision

The following character coding revisions were applied to the matrix of Ji et al. (2016) by JC. The character numbering follows Ji et al. (2016). See the table below for the correspondence of character numbers between this study and Ji et al. (2016).

Utatsusaurus 25 {0 -> 1}; 39 {0 -> ?); 119 {? -> 0}
Grippia 43 {1 -> ?); 50 {0 -> ?); 116 {? -> 0}; 140 {? -> 0}; 141 {? -> 0}; 142 {? -> 0}
Gulosaurus 56 {0 -> 1}; 76 {2 -> 1}; 82 {? -> 1}; 139 {0 -> ?); 151 {? -> 0}
Parvinatator 3 {0 -> ?); 7 {? -> 0}; 60 {1 -> 0}; 110 {1 -> 0}; 112 {0 -> ?)
Mixosaurus cornalianus 48 {? -> 1}; 55 {1 -> 0}; 78 {? -> 1}; 79 {? -> 1}; 80 {? -> 1}; 81 {? -> 0}; 138 {? -> 0}; 147 {? -> 0}; 163 {? -> 0}
Mixosaurus kuhnschnyderi 18 {? -> 1}; 19 {? -> 0}; 22 {? -> 0}; 27 {? -> 1}; 33 {? -> 1}; 34 {? -> 0}; 35 {? -> 1}; 48 {? -> 1}; 50 {? -> 0}; 55 {? -> 2}; 57 {? -> 0}; 59 {? -> 0}; 60 {? -> 0}; 66

{1 -> ?); 69 {? -> 1}; 70 {? -> 0}; 71 {? -> 0}; 72 {? -> 0}; 73 {? -> 0}; 74 {? -> 0}; 75 {? -> 0}; 76 {? -> 1}; 77 {? -> 0}; 78 {? -> 1}; 79 {? -> 1}; 81 {? -> 0}; 82 {? -> 1}; 83 {? -> 0}; 86 {? -> 0}; 87 {? -> 1}; 90 {? -> 1}; 91 {? -> 0}; 92 {? -> 1}; 93 {? -> 0}; 94 {? -> 0}; 95 {? -> 0}; 96 {? -> 0}; 145 {0 -> ?); 147 {? -> 0}; 148 {? -> 0}; 151 {? -> 1}

Phalarodon callawayi 56 {0 -> ?); 84 {0 -> ?); 116 {? -> 0}; 117 {? -> 0}; 119 {? -> 0}; 120 {? -> 1}; 121 {? -> 1}; 124 {? -> 0}; 126 {? -> 1}; 127 {? -> 0}; 128 {? -> 0}; 129 {? -> 0}; 130 {? -> 0}; 131 {? -> 0}; 132 {? -> 0}; 133 {? -> 1}; 134 {? -> 0}; 135 {? -> 0}; 136 {? -> 0}; 137 {? -> 0}; 138 {? -> 0}; 139 {? -> 0}; 140 {? -> 0}; 141 {? -> 0}

Cymbospondylus piscosus 20 {0 -> 1}; 68 {1 -> ?); 141 {0 -> ?)

Cymbospondylus buchseri 9 {0 -> ?); 116 {0 -> ?); 118 {0 -> ?); 120 {1 -> ?); 154 {0 -> ?)

Cymbospondylus nichollsi 6 {? -> 0}; 32 {? -> 1}; 43 {? -> 1}; 49 {? -> 0}

Xinminosaurus 26 {1 -> ?); 75 {? -> 0}; 114 {? -> 1}; 115 {? -> 0}

Besanosaurus 68 {1 -> ?); 89 {? -> 0}; 97 {? -> 0}; 107 {1 -> 2}; 162 {0 -> ?)

Guizhouichthyosaurus 91 {0 -> 1}; 107 {1 -> 2}

Callawayia wolonggangensis 77 {? -> 0}; 101 {? -> 1}; 110 {? -> 1}; 139 {0 -> ?)

Guanlingsaurus 25 {0 -> 1}; 72 {? -> 0}; 91 {0 -> 1}; 106 {1 -> 0}; 107 {1 -> 2}; 113 {1 -> 0}

Shastasaurus 30 {0 -> ?); 32 {? -> 1}; 55 {? -> 0}; 91 {0 -> 1}

Shonisaurus popularis 25 {0 -> ?); 27 {1 -> ?)

Shonisaurus sikanniensis 71 {? -> 2};72 {? -> 0}

Californosaurus 71 {? -> 2}; 74 {? -> 0}; 75 {? -> 0}; 76 {? -> 3}; 78 {? -> 1}; 79 {? -> 1}; 80 {? -> 0}; 82 {2 -> 1}; 83 {? -> 0}; 89 {? -> 0}

Toretocnemus 10 {1 -> ?); 26 {2 -> ?); 28 {? -> 0}; 112 {? -> 1}; 138 {2 -> 1}

Qianichthyosaurus zhoui 3 {? -> 0}; 26 {2 -> 1}; 38 {? -> 0}; 52 {? -> 0}; 73 {? -> 0}; 74 {? -> 0}; 79 {0 -> 1}; 139 {1 -> 0}; 154 {? -> 0}; 163 {? -> 1}

Qianichthyosaurus xingyiensis 56 {0 -> ?); 71 {0 -> 2}; 74 {1 -> 0}; 75 {? -> 0}

Callawayia neoscapularis 38 {? -> 0}; 66 {1 -> ?); 68 {1 -> ?); 86 {2 -> 1}; 104 {0 -> ?); 126 {0 -> 1}; 132 {? -> 1}; 138 {2 -> 1}

Hudsonelpidia 100 {? -> 1}; 130 {? -> 1}; 131 {? -> 0}; 132 {? -> 1}; 135 {0 -> ?); 136 {0 -> ?); 137 {0 -> ?)

Macgowania 16 {? -> 1}; 30 {0 -> ?); 48 {? -> 0}; 77 {0 -> ?)

Suevoleviathan 11 {? -> 0}; 47 {0 -> 1}; 97 {1 -> 0}; 115 {0 1 -> 1}; 139 {0 -> 1}; 149 {0 -> 1}; 160 {? -> 2}

Aegirosaurus 20 {1 -> 0}; 38 {1 -> ?); 101 {0 1 -> 1}; 113 {? -> 1}; 121 {0 -> ?)

Acamptonectes 48 {1 -> ?); 109 {1 -> ?)

Maiaspondylus 2 {0 -> 1}; 104 {? -> 0}; 105 {1 -> ?); 128 {1 -> ?); 129 {0 -> ?); 145 {0 -> ?); 146 {0 -> ?); 147 {? -> 1}

Arthropterygius 37 {1 -> ?); 40 {1 -> 0}; 46 {1 -> ?); 71 {1 -> ?); 100 {0 -> 1}; 108 {? -> 1}; 126 {? -> 0}

Athabascasaurus 18 {? -> 0}; 20 {1 -> 0}; 22 {0 -> 1}; 28 {? -> 1}; 30 {1 -> ?); 39 {0 -> 1}; 74 {1 -> ?); 118 {1 -> 2}; 125 {1 -> ?)

Sveltonectes 2 {1 -> ?); 23 {? -> 1}; 92 {1 -> ?); 93 {1 -> ?); 95 {0 -> ?); 129 {1 -> 0}; 157 {? -> 2}

Brachypterygius 6 {? -> 1}; 36 {0 -> 1}

Caypullisaurus 1 {? -> 0}; 2 {? -> 0}; 5 {? -> 0}; 6 {? -> 0 1}; 13 {0 -> 1}; 20 {0 -> 1}; 26 {? ->

2}; 31 {? -> 1}; 129 {? -> 0}; 145 {0 -> ?); 149 {1 -> ?)
Malawania 3 {0 -> ?)
Mollesaurus 6 {1 -> 0}; 43 {? -> 0}; 143 {1 -> ?); 146 {1 -> ?)
Leninia 12 {0 -> 1}; 13 {? -> 1}; 14 {? -> 1}; 20 {? -> 0}; 40 {? -> 0}; 86 {1 -> ?); 88 {0 -> ?); 89 {0 -> ?); 94 {1 -> ?); 96 {1 -> ?); 107 {1 -> ?); 108 {0 -> ?)
Hauffiopteryx 13 {1 -> 0}; 20 {1 -> 0}; 37 {? -> 0}; 56 {? -> 0}; 82 {2 -> 1}; 84 {? -> 1}; 104 {1 -> 0}; 110 {1 -> 0}; 112 {? -> 1}; 136 {0 -> 1}; 152 {? -> 0}; 160 {? -> 2}
***Platypterygius* australis** 48 {? -> 0}; 79 {0 -> 1}; 82 {0 -> 2}; 121 {? -> 0}; 141 {0 -> 1}; 148 {? -> 1}; 159 {? -> 1}
***Platypterygius* americanus** 46 {? -> 1}; 67 {1 -> ?); 82 {0 -> 2}; 116 {? -> 1}; 117 {? -> 2}; 118 {? -> 2}; 119 {? -> 1}; 120 {? -> 3}; 121 {? -> 0}; 134 {1 -> ?); 135 {0 -> ?); 139 {1 -> ?)
Cryopterygius 4 {0 -> 1}; 6 {1 -> 0}; 20 {1 -> ?); 26 {1 -> ?); 82 {0 -> 2}; 109 {0 -> 1}; 120 {3 -> 2}; 157 {1 -> 2}
Ophthalmosaurus icenicus 43 {? -> 0}; 48 {1 -> 0}; 66 {? -> 1}; 68 {? -> 1}; 118 {1 -> 2}
Ophthalmosaurus natans 14 {1 -> ?); 37 {? -> 1}; 38 {? -> 1}; 41 {? -> 2}; 42 {? -> 1}; 43 {1 -> 0}; 44 {? -> 1}; 48 {? -> 0}; 56 {? -> 0}; 76 {? -> 3}; 88 {0 -> 1}; 107 {2 -> ?); 128 {? -> 1}; 136 {1 -> 0}; 139 {1 -> 0}; 152 {? -> 1}
Sisteronia 146 {? -> 1}

Character Number Correspondence

The characters were renumbered in the present study relative to Ji et al. (2016) based on anatomical consistency rather than derivation, following editorial requirement. To facilitate comparison, below is a table for the correspondence between character numbers of the present study and Ji et al. (2016).

**This study**	***Ji et al.***								
		18	*16*	37	*32*	56	*51*	75	*64*
		19	*167*	38	*33*	57	*164*	76	*65*
1	*1*	20	*17*	39	*34*	58	*189*	77	*66*
2	*2*	21	*18*	40	*35*	59	*171*	78	*67*
3	*3*	22	*19*	41	*168*	60	*185*	79	*68*
4	*4*	23	*20*	42	*36*	61	*47*	80	*69*
5	*5*	24	*170*	43	*37*	62	*48*	81	*70*
6	*6*	25	*21*	44	*38*	63	*52*	82	*71*
7	*166*	26	*22*	45	*39*	64	*53*	83	*72*
8	*7*	27	*23*	46	*40*	65	*54*	84	*73*
9	*8*	28	*24*	47	*41*	66	*55*	85	*74*
10	*9*	29	*25*	48	*42*	67	*56*	86	*75*
11	*10*	30	*169*	49	*43*	68	*57*	87	*76*
12	*11*	31	*26*	50	*44*	69	*58*	88	*77*
13	*12*	32	*27*	51	*45*	70	*59*	89	*78*
14	*13*	33	*28*	52	*46*	71	*60*	90	*79*
15	*184*	34	*29*	53	*49*	72	*61*	91	*80*
16	*14*	35	*30*	54	*50*	73	*62*	92	*81*
17	*15*	36	*31*	55	*165*	74	*63*	93	*82*

94	*83*	115	*177*	136	*114*	157	*134*	178	*152*
95	*84*	116	*102*	137	*115*	158	*135*	179	*153*
96	*85*	117	*103*	138	*181*	159	*136*	180	*182*
97	*86*	118	*104*	139	*116*	160	*137*	181	*194*
98	*87*	119	*105*	140	*117*	161	*138*	182	*195*
99	*88*	120	*106*	141	*119*	162	*139*	183	*155*
100	*89*	121	*107*	142	*120*	163	*140*	184	*156*
101	*90*	122	*108*	143	*121*	164	*141*	185	*157*
102	*91*	123	*109*	144	*118*	165	*142*	186	*159*
103	*92*	124	*110*	145	*122*	166	*143*	187	*161*
104	*93*	125	*111*	146	*123*	167	*160*	188	*162*
105	*94*	126	*112*	147	*124*	168	*144*	189	*163*
106	*95*	127	*113*	148	*125*	169	*145*	190	*183*
107	*96*	128	*172*	149	*126*	170	*146*	191	*154*
108	*97*	129	*173*	150	*127*	171	*147*	192	*187*
109	*98*	130	*174*	151	*128*	172	*148*	193	*188*
110	*99*	131	*179*	152	*129*	173	*149*	194	*192*
111	*100*	132	*180*	153	*130*	174	*158*	195	*193*
112	*178*	133	*186*	154	*131*	175	*191*		
113	*175*	134	*190*	155	*132*	176	*150*		
114	*176*	135	*101*	156	*133*	177	*151*		

Character Description

1. Premaxilla dorsal process: (0) long; (1) short (modified from Maisch and Matzke, 2000:10)
2. Premaxilla ventral process: (0) long; (1) short (modified from Maisch and Matzke, 2000:9)
3. Maxilla anterior process: (0) reduced; (1) extending anteriorly as far as nasal or further anteriorly (Fischer et al., 2011:7)
4. Maxilla dorsal lamina: (0) absent; (1) present (Motani, 1999:2)
5. Maxilla prefrontal contact: (0) absent; (1) present (Maisch and Matzke, 2000:12)
6. Maxilla external naris contact: (0) present; (1) absent (Motani, 1999:3)
7. Maxilla longer than premaxilla: (0) true; (1) false
8. External naris orientation: (0) lateral; (1) dorsal (Motani, 1999:4)
9. Shallow groove anterior to the external naris: (0) absent; (1) present
10. Narial shelf: (0) absent; (1) present (Jiang et al., 2006:4)
11. Nasal anteriorly extending beyond external naris: (0) false; (1) true
12. Nasal parietal contact lateral to frontal: (0) absent; (1) present (Motani, 1999:7)
13. Nasal postfrontal contact: (0) no contact; (1) contact extensive, posterior extension of nasal separates frontal from prefrontal in dorsal view; (2) eliminated by prefrontal medially extension (Modified from Motani, 1999:6)
14. Descending process of the nasal on the dorsal border of the nares: (0) absent; (1) present (Fernández, 2007:2)

15. Nasals rostrally reaching snout tip: (0) false; (1) true
16. Processus narialis of prefrontal: (0) absent; (1) present (Fischer et al., 2011:11)
17. Supraorbital crest on prefrontal and postfrontal: (0) absent; (1) present (Maisch and Matzke, 2000:22)
18. Prefrontal–postfrontal contact: (0) absent; (1) present (Motani, 1999:8)
19. Prefrontal exposure in upper temporal fenestra: (0) absent; (1) present
20. Anterior orbital margin: (0) of regular rounded shape; (1) irregular (Maisch and Matzke, 2000:23)
21. Postfrontal medially extension: (0) over the anteriormost margin of upper temporal fenestra; (1) not over the anteriormost margin of upper temporal fenestra
22. Supratemporal antero-medial extension: (0) short; (1) long
23. Supratemporal-postorbital contact: (0) absent; (1) present (Fischer et al., 2013:15)
24. Supertemporal / squamosal relative size: (0) equal or st smaller; (1) st clearly larger
25. Sagittal eminence: (0) absent; (1) present but small, involving only the parietal; (2) present and large, involving the parietal, frontal and nasal (Motani, 1999:16)
26. Frontal dorsal exposure: (0) clearly present; (1) nearly absent
27. Frontal participation in upper temporal fenestra: (0) absent; (1) present
28. Squamosal triangular shape: (0) false; (1) true; (2) squamosal absent (Fischer et al., 2013:16)
29. Squamosal participation in upper temporal fenestra: (0) present; (1) absent; (2) squamosal absent (Motani, 1999:13)
30. Squamosal-quadrate articulation: (0) present; (1) absent
31. Postorbital postero-dorsal corner: (0) narrow, giving triradiate shape; (1) broad and triangular; (2) absent or round
32. Postorbital participation in upper temporal fenestra: (0) present; (1) absent (Motani, 1999:12)
33. Jugal anterior margin: (0) tapering, between lacrimal and maxilla; (1) broad and fan-like, covering ma
34. Jugal/quadratojugal lateral contact: (0) present; (1) absent (Motani, 1999:23)
35. Lower temporal arch between jugal and quadratojugal: (0) present; (1) lost (Fisher et al., 2013a:18)
36. Quadratojugal: (0) longer than tall; (1) taller than long.
37. Quadratojugal exposure: (0) quadratojugal small; (1) extensive; (2) small, largely covered by squamosal and postorbital (Fischer et al., 2013:17)
38. Parietal ridge: (0) absent; (1) present (Motani, 1999:17)
39. Parietal supratemporal process: (0) short; (1) long (Motani, 1999:18)
40. Parietals' anterior processes: (0) contacting each other anteriorly, eliminating frontal from pineal foramen; (1) narrowly separated anteriorly, forming parietal fork, and frontal dorsally visible along the pineal foramen; (2) widely open, resulting in absence of clear fork (Motani, 1999:19)
41. Parietal-frontal suture inter-digitation: (0) absent; (1) present
42. Anterior terrace of upper temporal fenestra: (0) absent; (1) present but small; (2) present and large (Motani, 1999:14)
43. Basioccipital peg: (0) clearly present; (1) absent or extremely reduced (Motani, 1999:29)
44. Basioccipital extracondylar area: (0) wide; (1) reduced to a narrow band of concavity (Motani, 1999:30)

45. Basioccipital/atlas articulation convexity: (0) flat or anterior; (1) posterior
46. Ventral notch in the extracondylar area of the basioccipital: (0) present; (1) absent (Fischer et al., 2012:19)
47. Pterygoid, transverse flange: (0) antero-lateral; (1) not well defined; (2) postero-lateral (Motani, 1999:26)
48. Basipterygoid processes: (0) short, giving basisphenoid a square outline in dorsal view; (1) markedly expanded laterally, being wing-like, giving basisphenoid a marked pentagonal shape in dorsal view (Fischer et al., 2011:18)
49. Interpterygoid vacuity: (0) present; (1) absent or extremely reduced (Maisch and Matzke, 1997:22)
50. Ectopterygoid: (0) present; (1) absent (Callaway, 1989:9)
51. Shape of the paroccipital process of the opisthotic: (0) short and robust; (1) elongated and slender (Fischer et al., 2012:20)
52. Stapes proximal head: (0) slender, much smaller than opisthotic proximal head; (1) massive, as large or larger than opisthotic (Fischer et al., 2013:24)
53. Cheek orientation: (0) lateral; (1) posterior (Motani, 1999:25)
54. Overbite: (0) absent or very slightly; (1) present (Motani, 1999:33)
55. Prenarial snout longer than the postorbital skull: (0) false; (1) true
56. Snout extremely slender: (0) no; (1) yes (Motani, 1999:34)
57. Snout, constriction: (0) absent; (1) present
58. Snout flattened: (0) false; (1) true
59. Scleral ring extensively ossified, filling or almost filling the orbit: (0) false; (1) true
60. Pineal foramen posterior to or between orbits: (0) posterior; (1) between
61. Angular lateral exposure at its maximum depth: (0) semi-equal to surangular; (1) clearly shallower than surangular; (2) much deeper than surangular (Motani, 1999:32)
62. Coronoid region: (0) slightly elevated or high; (1) flat (Jiang et al., 2006:13)
63. Root striations: (0) present; (1) absent (Fischer et al., 2013:4)
64. Plicidentine: (0) absent; (1) at least partly present (Motani, 1999:36)
65. Bony fixation of teeth: (0) present; (1) absent (Motani, 1999:43)
66. Tooth horizontal section: (0) circular; (1) disto-medially compressed; (2) lateral compressed (Motani, 1999:37)
67. Tooth size relative to the skull width: (0) over 0.1; (1) below 0.05 (Motani, 1999:39)
68. Dentigerous region in adult: (0) complete; (1) largely reduced; (2) edentulous
69. Dental groove: (0) present throughout jaw margin; (1) only present anteriorly; (2) absent (Motani, 1999:41, 42)
70. Anterior sockets: (0) present; (1) absent
71. Maxilla multiple tooth row: (0) absent; (1) present (Motani, 1999:40)
72. Dentary labial shelf: (0) present; (1) absent (Jiang et al., 2006: char14)
73. Posterior tooth crown: (0) conical; (1) rounded; (2) flat (Motani, 1999:38)
74. Tooth crown surface of at least one maxillary tooth with mesiodistal ridge: (0) false; (1) true (Jiang et al., 2006:18)
75. Ossified sternum: (0) absent; (1) present (Motani, 1999:50)
76. Ossified cleithrum: (0) present; (1) absent (Motani, 1999:51)
77. Clavicle orientation at proximal end: (0) oblique to sagittal plane; (1) transverse

78. Clavicle scapular process length distal to clavicular main body: (0) short; (1) long
79. Interclavicle anterior process separating clavicles: (0) present; (1) absent
80. Interclavicle posterior process: (0) rod-like; (1) triangular; (2) absent
81. Scapular blade shaft: (0) absent; (1) present at least proximally (Motani, 1999:47)
82. Scapula anterior flange: (0) complete; (1) emarginated; (2) absent (modified from Maisch and Matzke, 2000:70)
83. Scapula antero-proximal extension toward clavicle: (0) absent; (1) present
84. rominent acromion process of scapula: (0) absent; (1) present (Fischer et al., 2011:28)
85. Scapula posterior extension: (0) present; (1) absent (modified from Maisch and Matzke, 2000:71)
86. Scapular axis and glenoid facet orientations: (0) nearly parallel; (1) at 60 degrees or more (Motani, 1999:48)
87. Coracoid facet on scapula: (0) fused scapulocoracoid; (1) absent; (2) equal or smaller than glenoid facet of scapula; (3) twice as large as glenoid facet (Motani, 1999:49)
88. Coracoid parasagittal length vs. transverse width: (0) semi-equal or longer than wide; (1) clearly wider than long
89. Coracoid foramen: (0) present; (1) absent
90. Coracoid anterior notch or concavity: (0) absent; (1) present
91. Coracoid posterior notch or concavity: (0) absent; (1) present
92. Intercoracoid facet: (0) short, medial margin round shaped; (1) long, medial margin relatively straight (modified from Maisch and Matzke, 2000:73)
93. Humerus anterior flange: (0) absent; (1) present and complete; (2) present but reduced proximally, leaving leading edge tuberosity (Callaway, 1989:29)
94. Plate-like dorsal ridge on humerus: (0) absent; (1) present (Motani, 1999:56)
95. Protruding triangular deltopectoral crest on humerus: (0) present but small; (1) present and very large, bordered by concave areas (Fischer et al., 2013:39)
96. Humerus distal proximal width ratio: (0) nearly equal; (1) distal wider (Motani, 1999:55)
97. Humerus with posterodistally deflected ulnar facet and distally facing radial facet: (0) ulnar facet contains convexity in lateral view; (1) absent; (2) present (modified from Fischer et al., 2013:42).
98. Humerus distal articular facets: (0) not terminal; (1) radial facet larger than ulna facet; (2) two facet nearly equal; (3) three facets (Motani, 1999:52)
99. Humerus anterodistal facet for accessory zeugopodial element anterior to radius: (0) absent; (1) present (Fischer et al., 2013:41)
100. Humerus/intermedium contact: (0) absent; (1) present (Fernández, 2007:15)
101. Radius peripheral shaft: (0) complete or nearly complete; (1) notch or absent (modified from Motani, 1999:59) please see the text for detailed discussion.
102. Radius contiguous shaft: (0) about half or more of radial length; (1) notch or absent (modified from Motani, 1999:60) please see the text for detailed discussion.
103. Ulna peripheral shaft: (0) complete or nearly complete; (1) notch or absent (modified from Motani, 1999:62) please see the text for detailed discussion
104. Ulna contiguous shaft: (0) complete or nearly complete; (1) notch or absent (modified from Motani, 1999:63) please see the text for detailed discussion
105. Shape of the posterior surface of the ulna: (0) radius not discoidal; (1) rounded or straight and

nearly as thick as the rest of the element; (2) concave with a thin, blade-like margin (Fischer et al., 2012:36)

106. Radius/ulna relative size: (0) nearly equal; (1) radius much larger than ulna; (2) ulna larger than radius (Motani, 1999:64)
107. Radio-ulnar foramen: (0) present; (1) absent (Fischer et al., 2013:46)
108. Radiale, anterior notch: (0) absent; (1) present (Motani, 1999:65)
109. Manual pisiform: (0) present; (1) absent (Motani, 1999:67)
110. Manual pisiform 2 (neomorph): (0) absent; (1) present (Jiang et al., 2006:20)
111. Intermedium: (0) longer than wide; (1) as wide as long or wider than long (Jiang et al.,2006)
112. Proximal carpals: (0) packed; (1) small, round and separated
113. Distal carpal 1: (0) ossified; (1) unossified; (2) absent
114. Distal carpal 2: (0) ossified; (1) unossified; (2) absent
115. Manual centralia: (0) present; (1) absent
116. Metacarpal I peripheral shaft: (0) complete; (1) notch or largely reduced; (2) absent; (3) mc 1 not ossified (Motani, 1999:68)
117. Metacarpal III shaft: (0) present; (1) absent (Motani, 1999:69)
118. Metacarpal V: (0) present; (1) not ossified (Motani, 1999:70)
119. Manual digit 2 distal elements peripheral shaft: (0) complete; (1) notch or absent; (Motani, 1999:71)
120. Forelimb hyperphalangy with more than five phalanges ossified in longest digit: (0) absent; (1) present (Motani, 1999:77)
121. Notching of anterior facet of leading edge elements of forefin in adults: (0) elements not discoidal; (1) present; (2) absent (Fischer et al., 2013:48)
122. First preaxial accessory digits on forelimb: (0) absent; (1) present (Maisch and Matzke, 2000:91)
123. Postaxial accessory digit on forelimb: (0) absent; (1) only one; (2) more than one (Motani, 1999:72)
124. Proximal manual phalanges proximo-distal packing: (0) well-packed; (1) not packed
125. Manual digit between 4th and 5th digits: (0) absent; (1) present (Motani, 1999:73)
126. Propodial + epipodial versus manus length: (0) propodial + epipodial longer; (1) manus longer (Motani, 1999:58)
127. Forelimb/hindlimb ratio: (0) nearly equal or hind longer; (1) forelimb longer but less than twice as hindlimb; (2) forelimb longer twice as much as hindlimb (Motani, 1999:79)
128. Zeugopodium flattened: (0) false; (1) true
129. Manual anterior sesamoid: (0) absent; (1) present
130. Second preaxial accessory digit of forelimb: (0) absent; (1) present
131. Delayed mesopodial ossification: (0) present; (1) absent
132. Forelimb hypophalangy with less than five digits ossified in longest digit: (0) absent; (1) present
133. Carpus elongated, as long as the more distal forelimb part or longer: (0) false; (1) true
134. Extra proximal carpal preaxially: (0) absent; (1) present
135. Interdigital separation: (0) present; (1) absent (Motani, 1999:78)
136. Iliac blade shape: (0) with thick shaft; (1) plate-like; (2) narrow and styloidal (Motani, 1999:80)

137. Iliac antero-medial prominence: (0) absent; (1) present (Motani, 1999:81)
138. Ilium-pubis relative proximo-distal length: (0) semi-equal; (1) ilium clearly shorter
139. Pubis, styloidal or plate-like: (0) plate-like; (1) styloidal (Motani, 1999:85)
140. Pubis obturator foramen: (0) completely enclosed in pubis; (1) mostly in pubis but open on one side; (2) part of obturator fossa (Motani, 1999:84)
141. Pubis and ischium median symphysis: (0) present; (1) not well defined (modified from Maisch and Matzke, 2000:108)
142. Pubis and ischium fused in adult: (0) complete; (1) absent; (2) present only medially; (3) present medially and distally (Motani, 1999:83)
143. Pubis ischium relative size: (0) nearly equal or ischium slightly larger; (1) pubis twice as large as ischium (Motani, 1999:86)
144. Ischium, styloidal or plate-like: (0) plate-like, longer sagittally than wide transversely; (1) plate-like, wider transversely than long sagittally; (2) styloidal (modified from Motani, 1999:87)
145. Thyroid fenestra: (0) absent; (1) one median opening; (2) two openings, being medially separated (Motani, 1999:82)
146. Femur strongly constricted medially, forming a slender shaft region, proximal width remarkably larger than medial width: (0) present; (1) absent
147. Femur antero-distal expansion, forming a distinctive structure at the distal end: (0) absent; (1) present.
148. Prominent, ridge-like dorsal and ventral processes demarcated from the head of the femur and extending up to mid-shaft: (0) absent; (1) present (Fischer et al., 2011:46)
149. Wide distal femur blade: (0) absent, the proximal and distal extremity of the femur being sub-equal in dorsal view; (1) present (Fischer et al., 2013:61)
150. Femur distal facets: (0) two; (1) three (Maxwell, 2010:32)
151. Astragalus/femoral contact: (0) absent; (1) present (Maxwell, 2010:33)
152. Femur anterodistal facet for accessory zeugopodial element anterior to tibia: (0) absent; (1) present (Fischer et al., 2011:48)
153. Tibia contiguous shaft: (0) complete or nearly complete; (1) notch or absent (Motani, 1999:91)
154. Tibia peripheral shaft: (0) complete or nearly complete; (1) notch or absent (modified from Motani, 1999:92)
155. Tibia antero-proximal end nearly rectangular, forming a deep notch on the anterior margin: (0) absent; (1) present
156. Fibula posterior extent: (0) not fixed, fibula being mobile relative to femur; (1) much posterior to femur; (2) about the same level as femur (Motani, 1999:93)
157. Fibula contiguous margin: (0) concave; (1) nearly straight or convex
158. Fibula posterior flange: (0) absent; (1) present
159. Tibia and fibula: (0) in contact or closely placed with each other; (1) widely separated from each other (Motani, 1999:88)
160. Spatium interosseum between tibia and fibula: (0) present; (1) absent (Fischer et al., 2013:64)
161. Hind fin leading edge element in adults: (0) elements not discoidal; (1) notched; (2) straight (Fischer et al., 2013:65)
162. Postaxial accessory digit in hind limb: (0) absent; (1) present (Fischer et al., 2011:50)

163. Pes digit 1: (0) present; (1) absent (Motani, 1999:89)
164. Distal tarsal 2 in line with distal tarsals 3 and 4: (0) true; (1) false, distal tarsal 2 in line with calcaneum
165. Atlas/axis fusion: (0) absent; (1) present (Motani, 1999:94)
166. Presacral count: (0) less than 30; (1) between 40-52; (2) 55 or more (Motani, 1999:95)
167. Vertebra count between sacral and apical about: (0) tail stem not well defined; (1) 1/2 of the prepelvic count; (2) 2/3 of the prepelvic count or more
168. Posterior dorsal centra shape: (0) cylindrical; (1) discoidal (Motani, 1999:97)
169. Posterior dorsal/anterior caudal centra degree of shortening: (0) 3.5 times or less as high as long; (1) four times or more as high as long (Fischer et al., 2013:26)
170. Cervical bicipital rib facet: (0) absent; (1) present (Motani, 1999:99)
171. Rib articulation in thoracic region: (0) predominantly unicapitate; (1) exclusively bicapitate (Maisch and Matzke, 2000:53)
172. Antero-dorsal rib facets: (0) confluent with anterior facet in at least near pelvic girdle; (1) not confluent in any of the centra (Motani, 1999:101)
173. Posterior-dorsal bicipital rib facet: (0) absent; (1) present, might be resulted from the split of diapophysis (Motani, 1999:100)
174. Last caudal rib facet reaching the caudal peak area: (0) false; (1) true
175. Rib mid-shaft broadening rostro-caudally: (0) absent; (1) present
176. Sacral ribs: (0) at least two, distinguishable; (1) absent (Motani, 1999:104)
177. Anterior dorsal neural spine: (0) normal; (1) narrow (Motani, 1999:102)
178. Neural spines of atlas-axis: (0) functionally separate, never fused; (1) completely overlapping, may be fused (Druckenmiller and Maxwell, 2010:26)
179. Neural spine anticlination in tail: (0) absent; (1) present (Motani, 1999: 103)
180. Dorsal neural arch, transverse process: (0) present; (1) absent
181. Second dorsal segment on dorsal neural spines: (0) absent; (1) present
182. Anterior dorsal neural spines with thickened central axis and rostral and caudal median ridges or flanges: (0) absent; (1) present
183. Caudal peak with curved vertebral column, near anticlination of neural spine: (0) absent; (1) present (Motani, 1999:96)
184. Anterior caudal vertebral size about 1/2 of the largest dorsal vertebrae or less: (0) false; (1) true
185. Mid-caudal vertebrae height change: (0) gradually decrease; (1) increase; (2) sudden decrease (Motani, 1999:98)
186. Pre-flexural wedge-shaped centra: (0) absent; (1) present
187. Tail: (0) as long or longer than the rest of the body; (1) distinctly shorter (modified from Maisch and Matzke, 2000:65)
188. Lunate tailfin: (0) absent; (1) well developed lunate tailfin (modified from Maisch and Matzke, 2000:66)
189. Chevrons in apical region: (0) present; (1) absent (Sander et al., 2000:72)
190. Fluke vertebrae, laterally flattened and packed: (0) absent; (1) present
191. Posterior gastralia immediately craniad of pelvic girdle: (0) present; (1) absent (Motani, 1999:105)
192. Flat gastral elements with caudad one outlying the craniad one: (0) absent; (1) present

193. Dermal ossicle: (0) absent; (1) present
194. Dermal armor above caudal vertebrae with hemal arches: (0) absent; (1) present
195. Dermal armor above dorsal neural spines, first layer: (0) absent; (1) present

Character Matrix

Petrolacosaurus

000000000000000000000000000000000000000000?00?0?00??000000?000?000000000000
0000000000000000000000000000000000000000000000000000000000000000000000000000
0000000000000000000000000000000000?0000

Claudiosaurus

000100010000??00000000?000000000?101001010??0?0?10??0000000000?000000?010001
000000??0000000000?100000000000000100000000000000000000000000?00001001000000000
0000000000000?0?00000?00000000?0?000000

Thadeosaurus

??????0???0????????????110??0000
00000000000??00000000000000000000000000000000000000000010010000000000000000000
00?0000?0000000?00000000?0?0?0000

Hovasaurus

?????????????????0??00?000000000??0?0000?0?01?1?0?0???0????0?0????????????100?10
000000000000001?000000000000000000000000000000000000000000110000000000000000000
000000000010?00000?0001000000?000000

Wumengosaurus

001100101000000000000???000000000101000010??0?1?010?001010100??0??100?011001
0001121000201110000002000000000001001111000000000000100000000010001100000000000000
00000200??100000??000000000000000?000000

Nanchangosaurus

0??100?????0010000??000?000000000?010?1000??????????0????1?0???????2????????????
???????0100?10?0?20?0000000????????????????????1????0??????????????????????????????????1
?0?00?0?000??010?????????1101

Eohupehsuchus

0111001000000000?10100000000000001????0000??????????0?1001?0?0?????2????????00
?0000010?0000?10?0120000000000010000010000??000????1001?00000?0??????10010?00001000
0?????1?0?0?01?0?0???10????????01111

Hupehsuchus

01?1?0?00000000?0(01)?10???0?0?0??0???1?000?0??0?2?0???0010010000?????2????????
00??00??0020000010?0120000000000010000010000000000111001000010000?100110010000101
0000200001000?00001?00001(01)0000?0?001111

Parahupehsuchus

??1??????????????????????????
????????1??0120000000000010000010000000100001100100010?????????10?0000000100000000?
1?0??00??100?0?10????????01111

Eretmorhipis

??
????????1??1120000000000000000010000000100001110100100001?100110?0000000100000000
?1?0??000?100?00100????0??01111

Sclerocormus

001110100010011001001110000000?000????01200??????????0000101100????????????0100
00000011??????????????????1?1???101111010010100101110000101000011100010010000001000
02000?0200?0??010010101000000000001100

Cartorhynchus

11010000001000100000000?00000?00?1010010?1????????0?0000101100?????2???1???10
00000000???????20?0110010100000101111????1000010111000010100I???????1001?00000100
0020?0?0?0?00???100??100????????00000

Chaohusaurus geishanensis

??0???1???1??000?1?0??0????0??1?00010???????????????1011101111??110??1110?11???
00?0002?????10?01100000000000101010110000000001?1000100?????????????01?00???????020
??????0?0???0??0?100????0????0000

Chaohusaurus chaoxianensis

110100100010000000100000100000110000100000010?0?1?01??1011101111??1100?011001
1112000000201100 10?111000000000001011110100000000101110001001000011100110010000001
00002000?110000000000001100100 00??100000

Chaohusaurus zhangjiawanensis

???????????00????1??00??000?0??0?????000?1??0??????????1???1????????????????01?1??0
00000??????10?0110000000000?01?10??00??0???????????????00?00?100?10010?0000?00?0????
?110?00?0?100?11001?0?????00000

Utatsusaurus

0111001100100000000011001000011100110101001????0?11??0010101111??0110000100010
1000000001011101000010000000000000100011000000000000100100010000001001100100000001
0000000000011000?000000?1100100000?100000

Grippia

011100?1001000?00001101100000?1001101010?1????0?????0?101??111?10110001110?1??
??00000010111010010100000000000001000120000000101?10010001???00?????1001??????????
??0000??001??1???0??1???????????000??

Gulosaurus

00?1001100?0000000?10011000?1??0?????01001????0?????00101011?1?1??10??1?10????
??0000101011101?0?010000000000000000001000??0001???1001?001??????????????????????????
??????0010?0?0?0??100?????????0000

Parvinatator

???1?0?000?????0?0?0??11???011100?1????????????????????1?10?110?1?000?00100??????
????????????????0100000000000000000110001000000??10010001????????????????????????????
??????????????????????????????

Mixosaurus panxianensis

1111101000100000110010112000112?011?110102??????????0010101111??0?00000110011
11100000001011101001010010100000001100012000100000111001000110000011011001000000
00000000?111010?1?011?1100101000010000

Mixosaurus cornalianus

1111101000100000110011?1120??1121000?110102??1???11??0010101111??00100?0110011
111000000010111010010100001000000011000120001000001110010001?000011011001000000
00000000?11101001101?1100101000010000

Mixosaurus kuhnschnyderi

1???1?1??01?????11?010??20?????1?00??101?2????????????01?101111???200000110?????1
0000001011?01001010?1010000?????00????????????????1??0????????????????????????????????
?????00????1???????1????1?0???

Phalarodon atavus

111110?0011000?011001011200011210000110102????2?11??0?101??1??110200{12}00?00
?????1?00000??????1001?30000100000000100012000100000??1001???1??????????10?00??????0
0??000??1?1?????????1?1???1???0?0?0????

Phalarodon fraasi

11?110?0011????111?01?????????2?????????????????????????1?1?1?1??1?200100021?1?????
?00???0????1?0101??0010?0?00100001????????0???10010001?0???011??00?????000100????00
??????????0???11001??????1??000

Phalarodon callawayi

11111011011??00011?0??1120????2100011????2??????????0010101?1????0?0110021?????
???????1?????????????????????????0?????????????????????????1???00011???0?1000000100000000
0???000?????10?????????????????

Cymbospondylus piscosus

01110010001010000100011110101110000010110 1??0?2011??0010101110?1000020000001
?0??0100001000101000010?00100000???1????????????????1???????10000110011001000000100
00????021101000?010?1100100000?100000

Cymbospondylus buchseri

????0?????1???0?0100??1??0????110??????1????????????0010??1?10????0???010001?0??0100001?001?1?00010?00100000??11??????1????????1???????????????????????????????????????2?10?000?0?0??100?????????00?0

Cymbospondylus nichollsi

???100?0??101??00100111?101?1?1001101010?1??0?2?1???0?????1110????????????0100??????????0010??0??1?1000???0?????????????????

Xinminosaurus

??????1???0?1?101??0?1?2?0101120010012000000?1001?1001010000100000101011110000000001110010001100?011???10010000001000000?0?2110?0???010?110010000001?0000

Besanosaurus

???11?1???1?????01?0?????????????0????????????????????01110???1?1?0?0??0100??????020000201110100001001111101011???????1?112????1?????????1000111001100000000001000???????2?10100??010??100????0?0100000

Guizhouichthyosaurus

01110010{01}010100001000?01100011210??11??101??????????00101011111?1?0000001000111100200012111101000010011111110101020131011200101110010001100011100110000000000100001010?2?1010?0?010?11001???0001?0000

Callawayia wolonggangensis

0111001010101000001?000011010112{01}00012011?1????2?1???001{01}101111?1?000000100?????0????0??011101?0001001?11111?101????31011100101??????0?1?????????????????????????????2?10?0?0???????????????????0???

Guanlingsaurus

01111010001010000 1?000111010112{01}0????01001??????????00101011111?????2??????011????20001?1111?1000010011111110101120131110200101010011101100021100110000000001010010100 2110?00?1010?11001000001100000

Shastasaurus

????0?1???100???01?0000110100?2100?1101101?01?2011??0?1?????11?1?0????????011???120001211111100000100 11?11111???0??1?????1??????1001????10001110011000000000010000???0???101?????1??????1????????????

Shonisaurus popularis

??????1?????1???????????1??????????????1???01???????00??????11?1?0?1200???????1012000121111110000100101011 00???????311111???111???????10?011100110000000 0?10100??1???2?101??0?010??10?1??????100000

Shonisaurus sikanniensis

??????????1???0???111???0?12001?0?????012
0?0???111?10000100101011 0???????????????????????????????????????00000????01?0?????2?
1010?0???0????????????10?0??

Californosaurus

???200
0030110?10000200101010011 01???1????????1???1001???11100211001100000000110000?????1
210?01?1010?1100112????1?0600

Toretocnemus

????????????????????????????????0???????????????????001?1?1?10?110?0010100????????
???????0011?0?120010101001101020131111100001?10010001200001100101010001112000010
11???10?0?1??1???1?0?????????????

Qianichthyosaurus zhoui

11010010001000000 1?0??1110?1111{01}0???2????0?01???????0010101110 01?0?0??01000
1111??2000??011011000120010101001101020131111101001110010001200001100101010000 11
200002011?111010?11010?110011210?1100000

Qianichthyosaurus xingyiensis

10?100?00??0000011?0??1??0??????0????????????????????010101?1??????00?0?00??????
?20000?0??0?1000110010101000100????31111100001?????000120?011101?01010?00?120000?
????11100001?0?0?110010210?1?00000

Callawayia neoscapularis

11???01000101?0?01?00?01101?1121?????11101?01?2?????001010?111?110000001 0001??
????0?0??111101000110011101101001020131?1110010111001000120?021100110010001{01}12
10001?10022101000?0101?100?12?0??1?0000

Hudsonelpidia

????????????????????0????????????????????????????????001010?????????????????????????
?????????200112001111 1000??10??131011?01001?100100012101?1101?1001?001011????10???
?????????0?1??100????????10000

Macgowania

10000110001??00?01?0???????1??210??11???0???????????0010101110 01?0000?01000111?
?121?103?????2??01200111110011010201310111010 01?10010001?????????????????????????
????????????0?0??100?????????0000

Suevoleviathan

10000{01}100010100001?0??0110?1?12100111101?0??????????00101011110 1?0000?0100
0111??1210103?110120?112001111101010102013101120 10{01}11100100011101211012100100
01102100111110121?1?111010??1001?2?01?110000

Temnodontosaurus

1010011000111000011000011101112100111{01}100001020010000101011010110000101
0001111012?010301101{02}00112001111010101020131111101{01}0011100100012
0?121{12}01
2110100011021010101111210101?10101?10011210001?0000

Leptonectes tenuirostris

1000?110?0111000010000?101011121001111021010??1?01??1011101??011101001010001 1
11012101030110100011200111110{01}010102013101120000111001000120012110220001000 11
0210111011?121010111010??10011210??110000

Leptonectes moorei

1000?110?01{01}10000100???100??112100110?011???????????101110112011?0?00101000
1111012?01030110100011200111101010102013101120000 1?10010001?????11?????0??00?????
???????????0?0???0????100?????????00?0

Leptonectes solei

10?0?110?01?1???0?00???111???12????????2?????????????01110????1????????100011????
21?103011010??1?2??1111?0?0101120131?10?0010??10011101200121101200 0??001102101???
11?1?1??????0?0??100????0??1?0000

Excalibosaurus

10?00110001??00?01?0???1?1???12?0????????0??1???????1111101101?1101001010001111
01211103?1101200112001111011101020131111101001?1001000120???11?1?100100011021011
10?111?1?1??1?010??10011??000110000

Eurhinosaurus

100000100010?00001?0000101?1?12?0011210??0?01?????0011111011?1011010010100011
11012101030110?20?11200111101100102013111110100111001000120012110121001000110 21
011101111121010?1?0100?100112?000110000

Ichthyosaurus

1000011000102000011000110 00{12}21210011210210001020010000101011110110000101
000111101210103011110000120011111010{01}010201310112011012100100012001 21{12}0221
000000110210011011111?101111101{01}1{01}1001121110110000

Stenopterygius

10000{01}10001{01}20000110001110011121001121021 0?010200101001010111111100001
010001111012111030110100011200111101100102013101110{01}1{01}121001000120112 1302
210?00001102100110111121111110100{01}1(01)01121111110000

Aegirosaurus

10000110001011011 1?00?01?0111121101?2??2?0???????????0111011201????00?0?0?00?1
?0???????0100?01?1130111111210000020131011211001111110001 20?1213?1210?00001102100
12111?121??????0?0??1001???11?1?0000

Acamptonectes

???0?????????1???????????????????????0?????111?1??11?0?0?0????11?0??0????0??????12 11102011010101012310??112?1?0????????????1??1??1 0???1?0?0???00??????1?????0

Maiaspondylus

?10001?0????????????????????????0??????????????0??????????????01?0?00?0?00???????? ??????????01?01301??111?1?00??????10?????0??????????1????????????????????????????????1? ?111?0?????00?????????????

Arthropterygius

???110?0?????????????1????????????????????? ???0110101002310111110l0??1?????????21??????1????????????????100000?????????????1??11 1?1????????00??????1??????

Athabascasaurus

1111?0?00010?0??11?00?01010011211??01002?0?11??????10??0?011??11??0?0?0?00???1? ???20?121302210?????????????????11 ?10?????0?00??00????????????00

Sveltonectes

1?000110001011?111?0????001???2?1?1??01????11120???1?01010??0111?0?0??0?00????? 01211103011010110120011????100?102013101121100l?110?0?01???1213012101000011021001 2111?1210???1?0?0??1001?2???1????00

Brachypterygius

11100110?????1???1?0???1???????1111??????11111?1????0010101?0?1110000?0?00?????? ???1????????01?0130111110100?102013101121100l?11010001???????????????????????????? ???1???????????1???????????00??

Caypullisaurus

00100{01}100?1??100???0??11???0?12???111??????1?????????010101101?????1????????? 11012111030?1??011013101111101010102013111212001?11110001?????????1010000110210 0121?11??1??????0?0??100?????????0000

Malawania

??11??121 010??????2?0?12001111010?01????31011100001???????01?????????????????????????????1??1 ???0??0?0???00?????????????0

Mollesaurus

10?000?0????11?011?0???????0????1?112?????1110?10?010??????????1???00?0?00???1?? ???1??1 0???1?0????????????????????

Leninia

???00??0????11?111?00?01?00?111110???01110?110??????0???????1???????????????????
???
????????????????????????

Chacaicosaurus

??????10????????????????????????????????1011???????011101???????????????????????
??????????1???20011111011101010131011100001?100?0?01??????????1??????????????????1??
??1?????????????????????????

Hauffiopteryx

??00001000100001?1?00001?0011021011?1?02??001???????101110111????00??0?00????
?0120010?0110?1?1112001111101110102013101?100001?10011001??1121202210?10001102101
1??111121?11?1?0100?100???????110000

Platypterygius australis

1011101000101100110010 1?10122?21101111020001112101010010101120111000010?00??
????12111030110121101310111110100010201311112120 01?10111001???121302?101010111011
0012{01}1111?101111?0?00??001??11?1??0000

Platypterygius americanus

101001100010?10011?0??0??0?22?21?0111???????????????1001010110 1?1??00010?00?????
?121110??????21101300111110100010 20?31??????????10?1?001???121302?1011100??????????
??1??1011?1?0?0???00????????????00

Cryopterygius

1011001000???1?0?1?0????????????1011????????????????0010?01?20?1?000??0?000111?0
1211102011012?1022001111?0101010201310112110 01?10011?0120112120221010000110?10?1
????1121?11?1?010??100??2?1?1????00

Ophthalmosaurus icenicus

11000110001011001 1?000110001112110102102001 1102{01}0111001010110 01110?0010?0
0011110121110301101211023101111201000102013101121100121001 1?0120112130221010000 1
10210012011 1121{01}11111010{01}?10011211111?0000

Ophthalmosaurus natans

1100001000101 10??1?0001?100110211011 2?0200111?2101??00101011?011?0000?0?00011
1101211103011012 1?113101111?01?001020?310?????0???1??11001??????????10?11101102100
120111??1?11?1?0?01?100??????????000

Thalattoarchon

????0???????0???0?1?0111?101?10100????00201??????????0?????1??001?000????00??????
???10????????10?1???????????????????1
01011?0?0??1000????????????

Palvennia

??0?0?10??101?0??1?000??000????1??????020?1?11???????010101?0??1??00??0?00??????
?????????????????31??1???
?????????????????????????00

Sisteronia

??1??111?0??0???????????11?0??????0???????120
11?????????1??300???11
????????????????????????

Supplementary Data S2. New added characters and matrix for the relationships of diapsids

The matrix was modified from a recent study of marine reptile relationships[5]. *Sclerocormus* and five more new characters were added to the matrix. Given the small degree of modification, only the new added characters and coding are listed below.

Five new added Character States

214. Nasal, reaching the tip of snout: (0) false; (1) true
215. Flat gastral elements with caudad one outlying the craniad one: (0) absent; (1) present
216. Pineal foramen position relative to orbit: (0) posterior; (1) between orbits
217. Rostral and caudal median ridges on neural spines: (0) absent; (1) present
218. Carpus elongated, as long as the more distal elements or longer: (0) false; (1) true

Character matrix for the relationships of diapsids, with aquatic adaptations codes as ambiguous (Extended Data Fig. 2a)

Seymouriidae

0000000000000000000000100??00000010?0000000?00000000000000000000000000000
000000000000000000000000?00000001010(01)1100000000000100000000(01)1000?0??0
0000000000001000000000000000000000000000000000000000000000000000000000?

Synapsida

(01)0000000(01)(01)0000000000000??000011(01)0?100(01)0000101000(01)000000000
0000000000000000000000000000000(01)0000(01)0000000000(01)01(01)100000010000000
100000010?0000?0??00000000000(01)01000000000001000(01)0000000000000000000(02
)00000(01)0000000000000000000

Parareptilia

(01)00000(01)10000000(01)0000(01)1(01)??(01)0(01)010(01)0?(01)000000?(0)(01)100
00000000000000000(01)(01)(01)(01)000001(01)00(01)(01)0(01)(01)00002(01)010(01)1(01)
(01)(01)00(01)0(01)00000010{01}000000(01)000000011000(01)000?(02)000?0??(012)(01)0
0000?000000(01)0000(012)0(12)000(01)(01)000(01)00(01)0010000000000000(01)00000(01
)01000000000(12)000000{01}

Captorhinidae

000100010000000000000000??00001011?(01)000010?001000000?000000010000000000
0000100000001???0000?01000000001000001000000000000000?01000000000000001?000?
000001000000000000200000000000000000000000000020000000000000000000000000

Araeoscelidia

1000000100000?00000000000100010110000000000(01)0100000010000000010?00?000?
?0001000010000010010000100000000000000000000000110000?0100000001000010001?000
0000001010101000000(12)0000100000000000000000000001000100000000000000300010000000
0

Rhynchocephalia

0(02)000010100001?(12)000001100(01)0001(01)011(01)(02)1011(01)1(12)010(01)1(01
)0010(01)1(01)0111(01)111001(01)1100011010000100(01)2010101101011131(01)0000010

2(01)000(01)010(01)010001100000100(01)(12)100?00011100(01)0010001000(01)2010000
1000(01)0110111100(01)1111(01)(01)00(01)0000011000011100(01)0100000

Squamata

(012)0(01)000(01)0(01)00(012)010(12)0000(01)11000000(01)0(01)1(01)(012)(02)1011
0(12)2201??(01)(01)0(01)(01)(01)1001111111(01)01111(01)001(01)0011(01)010(01)01(012
)101011(01)0(01)11(01)311000001(01)2(01)0001(02)1010(01)000110(01)00001000(12)101
?0(02)01(01)10(01)10010101000(01)(02)11000(01)(01)010(01)011011(01)110111111(01)00
100001(01)1100001110020100000

Prolacerta

1100000101000?01011000000110000110001111012000110001001101110111001112?0
001001000000?0101011010100110100000010100001012010 0?0110010111?012?00??00110
0011000000000101220011010110101110010111010002000 00?02000003100101 0000?

Trilophosaurus

110000???0000???00111??1101000???1231111010??0????0?0?11111?01?110111000?1
00111?0100??2101011100??101002?0?11210001(12)100100?01??0101110000100?0??1100
01?1?000100?10020?00100011010111001011101000100000?020000011?110100?00

Rhynchosauria

01111010000000?011111011010000011112311111121001110000011111110011?001110
01100100001010101011110111112110200110200001011010 0?001001011100121 00?0?011
00011100101000101100011000110101111010111011003000 00112?0000110010100?00

Archosauriformes

(01)1(01)(01)101(01)(01)1000(01)0101110000(01)(01)00001010(02)(02)1?(01)1011(12
)0010(01)0010(01)1(01)111?011111?11?1(01)0110100001(01)1(01)1(012)1011(01)111001?
1110000(01)10(12)00001(012)10(01)(01)0(01)01010(01)1(02)112001(12)?00?0(12)0110001
1011001000(01)0110001(012)01011010111101(01)11100(01)(01)02000001(01)2(01)00001(
01)0(01)1(01)(12)00?00

Claudiosaurus

100000?110000?11010?0?0001?0011011001011022010011 00?001000?10???10???000
10010100000120100001?100100100000001010000?0110000?0100010101000010000001100
0100?000100011020001001000010110000000000001010000001000001011101110 0000

Coelurosauravus

00?0001111000???000???10000001 0011?011110221?0010?0????1?1110?11????1?????
???????????????1?01??????31?0?00??????0??010110?00?0?0001000?01??1??00????????0??
???0?00020?002?0?00???10100000000000101001001 00?0000110010?00???

Kuehneosauridae

010000101000001111 0?0000010001001020101102220001130101000 1111???001??100
0110?0010?00??0?011010001 1?010000000010?0010101100?111?1?0002000??0000001110
00000100100?02120?00????00?10211001??100?01?10000???0000011100?1000?00

Acerosodontosaurus

1???0??10????00?????0??00??0???0?1??????002001???????????0??0????????????????
????0????????11?????1?1????0??????0?00?00?0??0???0?0?01??????0????0?????????????00
100?20?10?1?????????????????????0?????????0???3????0??0???

Tangasaurus

???

?0????????????????????????????????????0??????0?????0??1???2???????????????1??0??0?100
???????00????????????01???????0????01?????0???0??0????00

Youngina

1000000000000001000?00?001?001101100??000110?000000100???0?10?1???????????
10??00??00100?01??????00??1000000101000000?0?0000?10??0?01001??????00????0?10
10?01?00100??0??0??0001??1??????000?10?0000000(01)???1??03????0?0?000

Thadeosaurus

???0????
????????????????????????????1?10????0?00?0??0??0?00?01001??000??0????0??010?010001
20?20010000000?011100??0?0?1?1000???00?000000?10?100?0?00

Lanthanolania

10???0?00?000?01????0?0????001?1????????012???????0?????????????????????????0??
????0??????????1??????????0????0?2???
????????????????????????????????????0???????????3???????0????

Orovenator

10?0000?1000000000010000?010001???0{02}101???1{12}?????0?0??????0?10?00?0??
??000100000?000000?10??0?00001?01?000000000?0?00????????????????????????????????
???0????000?????????3???????0????

Sophineta

000000101000?101000?11102000?01?11001?11022000010????????1111????????????01
?0?10???0000010??0????????310?0000???0?0?00?1??1?0010?0???01???????????????????????
????????????????????0???????????????000?????00????????010????????0??

Pamelina

010000101000??11110000000101?10?1020101102220?0113????????1111??????????11?01
?0??0???00??0101?0?0?????010?00?0?010?0?10?0??0?0101?11??02???????????????????????
????????????????????????????????????0??1??00??????????11??????????

Tanystropheus

11000011?1000?01011?00100010010111101111012201??0001001101110111?1111?10
01001000000002010100101001101000000011210001012110(012)?01100101020000?00?000
11000110000000001012100120101111011100011110020000010010200001310010100000

Choristodera

211000010101010111(01)00?1100000010?(01)2111110110?(01)001001011101110111?
0011?0??110010001000011010010?0??10110000010100001110100010(01)1001100100011
00?00111000101?00010001(02)1120010010(01)0?101110?101110101010010010001000100
110100000

Macrocnemus

11000011?1000?01?11?0?1?01?000001021111101210?????0??0?1?1011?11?01?1110?
1?0??0??00???0101001?1001101?0000010110001012110??00100101?10011?000000110001
10000000001?122001?000111101110000111010000000100102000?131?010100000

Hovasaurus

?????????????????????0??00?101?0??1?0?0??002011??00?10????01???01?0???1???????
?????????2?????1????????????????0?0???00000?0??0???0?01?10002??0?000010?????100010
00120?2?010?10000?01?10000?0??101020???00000000?300?100?0010

Pachypleurosaurus

1000001000(01)00??2010?0??0010011111001(01)11022010111?0?10?1011101??1???
11?1001111?0111???0?010010?111?11000000102100010101110101000110020002?00?001
1001211?0200(01)1101(01)01(12)00120100011011000011000001 1(01)10???(01)?????0????
???1000?0

Simosaurus

200000101010 0??2010?0??0000001101111101102201011 1?010011001101??1???10?10
00111?0110002110110 10?11??1100011011211001110111010 10?111102000??00?00110012
1000200111011112001201?0011?110000110000211?10?????????0?2??????000?0

Placodus

001001110101 0??2010?11?0010101011110111100201 0?01?010010011101??1?1?1101
102001101 10?0100110111100011110110110210100010110101100110102000 1?00?111110
111000000111010112001201000110111000110000(02)11220????????0??2??0??100000

Pistosauridae

10?000101010 0??2011?0??01100011011131011012011??1?0?0111001101??1???11?10
01011?0110?0?1?0???10?11??10000110112100011 10??1010100?1?102(01)0???(01)0?0(01)
11101?1??0200?11011112011001010(01)?01?000????001??0(012)1???????????2??????000
00

Askeptosaurus

1000011100100001001 00?0010101010100011110121 01??0101?11101111?11?01?1?00
01?0?0100001121100011001 11?010000001121000101011011010001010 10?000000?001000
010000000000101210110010 00?101110000111000102?1??0?0?00000?????100000

Clarazia

001100110010 0???001?0????11011100?00?1110122?1??010??111?1111???????1?0001
10?010??0?1?0111011?000??3110000111011 10?0?0110?0010?0?01010?01?000???110?010
0000000?0????2001?00?00??11110?0111000011 0?0???????000?0?????100000

Thalattosaurus

1011001100100???011?01?0?110?1100?00?111012201??010??111?1111???????1???01
?0?01???0?1?1111011000111211000 01?12011?1010?00?10???00??01???????????????????
100?00????????1?????????01??????????01???1??????0?00???????1000??

Helveticosaurus

00?1001?1?????0?000?0?1?0????1??11???0???
????????0?0?001??000?00??0000????00?0?01101?101001?10200?1?00?100110001000000
10001?111001?010000100110??111000?11320?0????0???0?????0??0000

Largocephalosaurus

00?0001?00000??201110?100110001011310111020210?1?10??0???0010????????1?0?1
?21?100?0???100000??000??01?0(01)0?0??2??01101?1110?101000(01)??2(23)2?00100011
1101210002001?101(02)1210110010(01)00?0011??0(01)100??(02)10100????????????????
0000?0

Sinosaurosphargis

00?0001000000???01110?1001110?00??30011102021 0?1??0??0???010??????1??00??
?210100?0002110000???0???01?00010?121001?00?1??2?20????0??202???1010211101 2???
????1?10????10110000?????????????????2??100?????????????????100???

Wumengosaurus

10?0011100001?0100110?00010?(01)010?1???0110221?0111??????1?01?0???????????

???????0??????0?01001?0000??1?10000?????00101?110??0100001?020?00?000000101101
0002000?001302200100101 00?0110??0??0000?11311?????????0????????00000
Nanchangosaurus
10?0?01?0?000?0100110?0001111001111010110221001100 1?00???00?0?????????????
??1?????????0??100???0??????02????????0?1?1??10?00100000002010111012121100?1??0
000?00113122?11?1?00???001??????????01020???????????????????01000
Hupehsuchus
10?0?01101000?0100111?0001111001111000110221001100 1??0???00?0????????????0
??11?0??1???0?0100???0?0????02????12100?101?110?00100000002010111012121100?1??
0000?00113122111111 0001?00100100100000 1020????????????????????010{01}0
Chaohusaurus
10?0011101001?0100111?001120000101001011022100110 01??0???0110?????????0??
0?01?100?1???0?00001??001?01?10000112101 0100?110?0210100(01)1010000100000 2100
0210?00000000113122111(01)011000?1010000111 00001(01)001??????????????????00100
Utatsusaurus
10?0011101001?0101110?0?1121000111101111020100110 01??0???001?????????????0
?01010??1???1100001?0011?01?10000111100010 1?110??2101?010010000?0000021100010
000000000113122?11(01)011000?10100100110000 10001???????0???1??????00100
Cartorhynchus
00?0011101001?0100010?00002(01)00100100111102210011001??0?1?01?0??????????
???????????????0?00001??000????12?????????0?100?110?0210??0?101000011003021100 01
0000000001131221 11?0?10?0?001??????????01?010??????????????????10101
Sclerocormus
00??011??1001?0100?11?100001001001000111{01}200??0?00????????1?????????????
??????????????1???0???????????12?????????0???0??1???2100?0??0100?0?10131211???1???
0???0?1?????????0?100??001??????????01?000???????????1??????11111

Character matrix for the relationships of diapsids including aquatic adaptations (Extended Data Fig. 2b)

Character Matrix

Seymouriidae
000000000000000000000100??00000010?0000000?00000000000000000000000000000
0000000000000000000000000?00000001010(01)1100000000000001 00000000(01)1000?0??0
0000000000001000000000000000000000000000000000000000000000000000000000000?
Synapsida
(01)0000000(01)(01)0000000000000??000011(01)0?100(01)0000101000(0)0000000000
00000000000000000000000000000(01)0000(01)0000000000(01)01(01)1000000100000001
00000010?0000?0??00000000000(01)0100000000000001000(01)000000000000000000(02)
00000(01)0000000000000000000
Parareptilia
(01)00000(01)10000000(01)0000(01)1(01)??(01)0(01)010(01)0?(01)000000?(01)(01)10

000000000000000000(01)(01)(01)(01)000001(01)00(01)(01)0(01)(01)00002(01)010(01)1(01
)(01)(01)00(01)0(01)00000010{01}000000(01)000000011000(01)000?(02)000?0??(012)(01)
00000?000000(01)0000(012)0(12)000(01)(01)000(01)00(01)0010000000000000(01)00000(0
1)01000000000(12)000000{01}

Captorhinidae

0001000100000000000000??00001011?(01)000010?001000000?00000001000000000
00001000000001???0000?010000000010000010000000000000?0100000000000001?000?
0000010000000000002000000000000000000000000000002000000000000000000000000

Araeoscelidia

1000000100000?000000000000100010110000000000(01)0100000010000000010?00?000?
?00010000100000100100001000000000000000000000000110000?010000000100001001?000
000000101010100000(12)0000100000000000000000000100010000000000000030001000000
0

Rhynchocephalia

0(02)000010100001?(12)000001100(01)0001(01)011(01)(02)1011(01)1(12)010(0)1(01)
0010(01)1(01)0111(01)111001(01)1100011010000100(01)2010101101011131(01)00000102
(01)000(01)010(01)0100011000000100(01)(12)100?00011100(01)0010001000(01)20100001
000(01)0110111100(01)1111(01)(01)00(01)000001110000111100(01)0100000

Squamata

(012)0(01)000(01)0(01)00(012)010(12)0000(01)11000000(01)0(01)1(01)(012)(02)1011
0(12)2201??(01)(01)0(01)(01)(01)1001111111(01)01111(01)001(01)0011(01)010(01)01(012
)101011(01)0(01)11(01)311000001(01)2(01)0001(02)1010(01)000110(01)00001000(12)101
?0(02)01(01)10(01)10010101000(01)(02)11000(01)(01)010(01)011011(01)110111111(01)00
100001(01)1100001110020100000

Prolacerta

1100000101000?0101100000011000011000111101200011000100110111011100111?0
001001000000?010101101010011010000001010000101201000?0110010111?012?00??00110
0011000000000101220011010110101110010111010002000000?0200000310010100000?

Trilophosaurus

110000???0000???00111??1101000???1231111010??0????0?0?11111?01?110111000?1
00111?0100??2101011100??101002?0?11210001(12)100100?01??0101110000100?0??1100
01?1?000100?10020?00100011010111001011101000100000?020000011?110100?00

Rhynchosauria

0111101000000?011111011010000011112311111121001110000011111110011?001110
011001000010101010111101111121102001102000010110100?00100101110012100?0?011
0001110010100010110001100011010111101011101100300000112?0000110010100?00

Archosauriformes

(01)1(01)(01)101(01)(01)1000(01)0101110000(01)(01)00001010(02)(02)1?(01)1011(12
)0010(01)0010(01)1(01)111?011111?11?1(01)0110100001(01)1(01)1(012)1011(01)111001?
1110000(01)10(12)00001(012)10(01)(01)0(01)01010(01)1(02)112001(12)?00?0(12)0110001
1011001000(01)0110001(012)0101101011101(01)11100(01)(01)02000001(01)2(01)00001(
01)0(01)1(01)(12)00?00

Claudiosaurus

100000?110000?11010?0?0001?0011011001011022010011000?001000?10????10???000

1001010000012010000l?1001001000000101000O?0110000?0100010101000010000001100
0100?00010001102000100100001011000000000000101000001000001011101110000O
Coelurosauravus
00?0001111000???000???10000001001l?011110221?0010?0????1?1110?11????1?????
???????????????1?01??????31?0?00??????0??010110?00?0?0001000?01??1??00????????0??
???0?00020?002?0?00???1010000000000101001001 00?0000110010?000??
Kuehneosauridae
010000101000001111 0?000001000100102010110222000113010100011 11???001??100
0110?0010?00??0?0110100011?010000000010?0010101100?111?1?0002000??0000001110
00000100100?02120?00????00?10211001??100?01?10000???0000011100?1000?00
Acerosodontosaurus
1???0??10????00?????0??00??0???0?1??????002001???????????0??0????????????????
????0????????11?????1?1????0??????0?00?00?0??0???0?0?01??????0????0??????????????00
100?20?10?1?????????????????????0?????????0???3????0??0???
Tangasaurus
??
?0??????????????????????????????????0??????0?????0??1???2???????????????1??0??0?100
???????00???????????01???????0????01?????0???0??0????00
Youngina
1000000000000001000?00?001?001101100??000110?000000100???0?10?1???????????
10??00??00100?01??????00??1000000101000000?0?0000?10??0?01001??????00????0?10
10?01?00100??0??0??0001??1??????000?10?0000000(01)???1??03????0?0?000
Thadeosaurus
???0????
???????????????????????????1?10????0?00?0??0??0?00?01001??000??0????0??010?010001
20?20010000000?011100??0?0?1?1000???00?000000?10?100?0?00
Lanthanolania
10???0?00?000?01????0?0????001?1????????012???????0??????????????????????????0??
????0?????????1?????????0????0?2???
????????????????????????????????0??????????3??????0????
Orovenator
10?0000?100000000010000?010001???0{02}101???1{12}?????0?0??????0?10?00?0??
??000100000?000000?10??0?00001?01?000000000?0?00????????????????????????????????
??0????000?????????3??????0????
Sophineta
000000101000?101000?11102000?01?11001?11022000010????????1111???????????01
?0?10???0000010??0????????310?0000???0?0?00?1??1?0010?0???01?????????????????????
???????????????????0?????????????000?????00????????010???????0??
Pamelina
010000101000??1111000000010l?10?102010110222 0?0113???????1111????????11?01
?0??0???00??0101?0?0?????010?00?0?010?0?10?0??0?0101?11??02?????????????????????
??????????????????????????????????0??1??00?????????11??????????
Tanystropheus
11000011?1000?01011?001000100101111011110122 01??00010011011 10111?1111?10

0100100000000201010010100110100000011210001012110(012)?01100101020000?00?000
11000110000000001012100120101111011100011110020000010010200001310010100000
Choristodera

211000010101010111(01)00?1100000010?(01)2111110110?(01)001001011101110111?
0011?0??110010001000011010010?0??10110000010100001110100010(01)1001100100011
00?00111000101?00010001(02)1120010010(01)0?101110?101110101010010010001000100
110100000

Macrocnemus

11000011?1000?01?11?0?1?01?000001021111101210?????0??0?1?1011?11?01?1110?
1?0??0??00???0101001?1001101?0000010110001012110??00100101?10011?000000110001
10000000001?122001?000111101110000111010000000100102000?131?010100000

Hovasaurus

???????????????????0??00?101?0??1?0?0??002011??00?10????01???01?0???1???????
?????????2?????1??????????????0?0???00000?0??0???0?01?10002??0?000010?????100010
00120?2?010?10000?01?10000?0??101020???00000000?300?100?0010

Pachypleurosaurus

1000001000(01)00??2010?0??00100111111001(01)11022010111?0?10?1011101??1???
11?1001111?0111???0?010010?111?11000000102100010101110101000110020002?00?001
1001211?0200(01)1101(01)01(12)001201000110110000110000011(01)10111(01)10111013
0112(01)1000?0

Simosaurus

2000001010100??2010?0??00000011011111011022010111?010011001101??1???10?10
00111?011000211011010?11??110001101121100111011101010?111102000??00?00110012
10002001110111112001201?0011?110000110000211?10111?10111012011212000?0

Placodus

0010011101010??2010?11?00101010111101111002010?01?010010011101??1?1?1101
10200110110?01001101111000111101101102101000101101011001101020001?00?111110
111000000111010112001201000110111000110000(02)11220111010110112010?1100000

Pistosauridae

10?0001010100??2011?0??01100011011131011012011??1?0?0111001101??1???11?10
01011?0110?0?1?0???10?11??1000011011210001110??1010100?1?102(01)0???(01)0?0(01)
11101?1??0200?11011112011001010(01)?01?000?????001??0(012)1111??01111?2?1?21?0
0000

Askeptosaurus

100001110010000100100?00101010101010001111012101??0101?11101111?11?01?1?00
01?0?010000112110001100111?01000000112100010101101101000101010?000000?001000
01000000000010121011001000?101110000111000102?1110000000000301121100000

Clarazia

0011001100100???001?0????11011100?00?1110122?1??010??111?1111???????1?0001
10?010??0?1?0111011?000??311000011101110?0?0110?0010?0?01010?01?000???110?010
0000000?0????2001?00?00??11110?01110000110?011?0?0?000?00?1??100000

Thalattosaurus

1011001100100???011?01?0?110?1100?00?111012201??010??111?1111???????1???01

?0?01???0?1?11110110001112110000l?12011?1010?00?10???00??01???????????????????
100?00????????1?????????01??????????01???111????0?00?3011??1000??
Helveticosaurus
00?1001?1?????0?000?0?1?0????1??11???0???
????????0?0?001??000?00??0000?????00?0?01101?101001?10200?1?00?1001100010000000
10001?111001?010000100110??111000?1132010????01110301120 2?0000
Largocephalosaurus
00?0001?00000??201110?1001100010113101110202l0?1?10??0???0010????????1?0?1
?21?100?0???100000??000??01?0(01)0?0??2??01101?1110?101000(01)??2(23)2?00100011
1101210002001?101(02)1210110010(01)00?0011??0(01)100??(02)10100111010111213011
?10000?0
Sinosaurosphargis
00?0001000000???01110?1001110?00??3001110202l0?1??0??0????010??????1??00??
?210100?0002110000???0???01?00010?121001?00?1??2?20????0??202???10102111012???
????1?10????10110000?????????????????2??10011????1??2?401??1100???
Wumengosaurus
10?0011100001?0100110?00010?(01)010?1???0110221?0111??????1?01?0???????????
???????0??????0?01001?0000??1?10000?????00101?110??0100001?020?00?000000101101
0002000?0013022001001010 0?0110??0??0000?11311111010111013011?1200000
Nanchangosaurus
10?0?01?0?000?0100110?000111100111101011022100110 01?00???00?0?????????????
??1?????????0??100???0??????02?????????0?1?1??10?001000000020101110121211 00?1??0
000?00113122?11?1?00???001??????????01020?1110??11?1?3011?1201000
Hupehsuchus
10?0?01101000?0100111?00011110011110001102210011001??0???00?0????????????0
??11?0??1???0?0100???0?0????02????12100?101?110?0010000000201011101212110 0?1??
0000?00113122111111000l?001001001000001020?11101011111301121201 0{01}0
Chaohusaurus
10?0011101001?0100111?001120000101001011022100110 01??0???0110?????????0??
0?01?100?1???0?00001??001?01?1000011210101 00?110?0210100(01)10100001000002100
0210?00000001131221 11(01)011000?10100001110000 1(01)001111010111113011(012)120
0100
Utatsusaurus
10?0011101001?0101110?0?11210001111011110201001100 1??0???001?????????????0
?01010??1???1100001?0011?01?10000111100010 1?110??2101?01001000 0?0000021100010
0000000011312 2?11(01)011000?101001001100001000111101010111101121200100
Cartorhynchus
00?0011101001?0100010?00002(01)001001001111022100110 01??0?1?01?0??????????
???????????????0?00001??000????12?????????0?100?110?0210??0?101000011003021 10001
000000000113122111?0?10?0?001??????????01?0101110???111?3011?1210101
Sclerocormus
00??011??1001?0100?11?10000100100100011 1{01}200??0?00????????1?????????????
??????????????1???0????????????12?????????0???0??1???2100?0??0100?0?10131211???1???
0???0?1?????????0?100??001??????????01?00011?0?0?11?11??1??211111

Supplementary Table S1. Measurements (in mm) of the holotype of *Sclerocormus parviceps* gen. et sp. nov. (AGB6265).

Length of skeleton	1599.3
Length of skull along the mid-line	76.3
Length of skull from the tip to the posterior margin of the supratemporal	100.0
Length of dorsal trunk	602.5
Length of tail	920.5
Length of the prenarial snout	19.0
Length of the constricted narrow premaxillary snout	22.1
Length of the preorbital snout	35.1
External naris opening	ab3.8
Anterior-posterior length of the orbit	36.3
Anterior-posterior length of the upper temporal fenestra	ab36.7
Anterior-posterior length of the pineal fossa	9.1
Width of the parietal skull table at the level of pineal fossa	19.7
Depth of the post occipital embayment	24.5
Number of cervical vertebrae	5
Number of dorsal trunk vertebrae	ca. 29 (14+15?)
Number of sacral vertebrae	2
Number of caudal vertebrae	ca. 67
Height of neural spine of 1st trunk vertebra	47.0
Width of neural spine of 1st trunk vertebra (at mid height)	13.2
Height of neural spine of 5th trunk vertebra	43.4
Width of neural spine of 5th trunk vertebra	13.9
Length of 5th rib	ca. 182.6
Width of proximal end of 5th trunk rib	22.4
Width of distal end of 5th trunk rib	10.4
Length of the 1st sacral vertebra, preserved part	14.8
Height of the 1st sacral vertebra	17.2
Length of the 2nd sacral vertebra	15.5
Height of the 2nd sacral vertebra	16.8
Length of the 1st caudal vertebra	15.4
Height of the 1st caudal vertebra	17.5
Length of the 5th caudal vertebra	14.0
Height of the 5th caudal vertebra	18.8
Length of the top of neural spine of the 5th caudal vertebra	15.9
Height of the neural spine of the 5th caudal vertebra	18.7
Length of the 1st sacral rib	19.3

Width of the proximal end of the 1st sacral rib	7.6
Width of the distal end of the 1st sacral rib	11.6
Length of the 2nd sacral rib	17.5
Width of the proximal end of the 2nd sacral rib	9.3
Width of the distal end of the 2nd sacral rib	6.9
Length of the 1st caudal rib	15.5
Width of the proximal end of the 1st caudal rib	7.5
Width of the distal end of the 1st caudal rib	3.2
Length of the dorsal process of the right clavicle	68.6
Width of the lateral corner of the right clavicle	10.6
Length of the dorsal-ventral diameter of the scapula	47.8
Antero-posterior length of the scapula	34.9
Length of preserved carpal and hand	170.8
Length of the preserved carpal space	103.0
Length of the preserved metacarpal-phalanges	67.8
Width of the preserved distal end of the ulna	33.4
Largest diameter of the ulnare	16.1
Largest diameter of the preserved intermedium	15.7
Largest diameter of the radiale	5.8
Largest diameter of the centralia	11.6
Largest diameter of the distal carpal 5	13.3
Largest preserved diameter of the distal carpal 4	12.5
Length of 1st metacarpal	10.6
Length of 2nd metacarpal	10.5
Length of 3rd metacarpal	12.4
Length of 4th metacarpal	12.5
Length of 5th metacarpal	9.1
Length of the right ilium	33.3
Width of the dorsal end of the right ilium	17.2
Width at the medial part of the right ilium	12.4
Width of the ventral end of the right ilium	21.6
Antero-posterior length of the right pubis	24.0
Latero-medial length of the right pubis	22.7
Antero-posterior length of the right ischium	29.1
Latero-medial length of the left ischium	>19.7
Length of tarsal and foot	113.9
Length of the preserved tarsal space	53.5
Length of the preserved metatarsal-phalanges	60.4

Length of the right femur	40.2
Width of the proximal end of the right femur	22.1
Width at the medial part of the right femur	22.3
Width of the distal end of the right femur	22.9
Length of the right tibia	27.0
Width of the proximal end of the right tibia	16.7
Width at the medial part of the right tibia	12.3
Width of the distal end of the right tibia	14.8
Length of the right fibula	32.4
Width of the proximal end of the right fibula	16.5
Width at the medial part of the right fibula	12.5
Width of the distal end of the right fibula	19.9
Largest diameter of the astragalus	13.4
Largest diameter of the calcaneus	11.6
Length of 1st metatarsal	5.1
Length of 2nd metatarsal	8.5
Length of 3rd metatarsal	10.9
Length of 4th metatarsal	11.2
Length of 5th metatarsal	9.3

Supplementary Table S2. Data source for Fig. 4. Values for non-shastasaurids were measured from published reconstructions with scales. *Cartorhynchus* and Shastasaurid values are from the text of references. The body length of *Cartorhynchus* assumes a tail/body proportion of *Chaohusaurus* but it may be as large as 472 mm based on the proportion in *Sclerocormus* (i.e., the point would shift right in Fig. 4a).

	Body Length (mm)	Skull Length (mm)	Source
Chaohusaurus sp.	990	119	AGM L-4
Chaohusaurus sp.	763	101	Fig. 3 in ref[4]
Chaohusaurus newborn	183	35	Fig. 4 in ref[4]
Cartorhynchus lenticarpus	~400	58	Ref[2]
Hupehsuchus sp.	885	134	Fig. 1 in ref[5]
Utatsusaurus hataii	2562	347	Fig. 68 in ref[6]
Guizhouichthyosaurus tangae	5400	855	ref[7]
Guanlingsaurus liangae	8300	689	ref[8]
Mixosaurus cornalianus	751	188	Fig. 68 in ref[6]
Temnodontosaurus platyodon	6879	1467	Fig. 68 in ref[6]
Ophthalmosaurus icenicus	4409	1063	Fig. 68 in ref[6]
Stenopterygius quadriscissus	2332	439	Fig. 4 in ref[4]
Stenopterygius newborn	641	201	Fig. 4 in ref[4]
Sclerocormus parviceps	1601	100	Present study

1. Ji C., JIang D.-y., Motani R., Rieppel O., Hao W.-c., Sun Z.-y. Phylogeny of Ichthyopterygia incorporating the recent discoveries from South China. *J Vertbr Paleontol* 36(1), e1025956, doi: 10.1080/02724634.2015.1025956 (2016).
2. Motani R., Jiang D.-y., Chen G.-b., Tintori A., Rieppel O., Ji C., Huang J.-d. A basal ichthyosauriform with a short snout from the Lower Triassic of China. *Nature* **517**, 485-488 (2015)
3. Chen X., Motani R., Cheng L., Jiang D., Rieppel O. The enigmatic marine reptile Nanchangosaurus from the Lower Triassic of Hubei, China and the phylogenetic affinity of Hupehsuchia. *PLoS One* 9(7), e102361, doi:10.1371/journal.pone.0102361 (2014).
4. Motani, R., Jiang, D. Y., Tintori, A., Rieppel, O. & Chen, G. B. Terrestrial origin of viviparity in Mesozoic marine reptiles indicated by Early Triassic embryonic fossils. *Plos One* **9**, doi:10.1371/journal.pone.0088640 (2014).
5. Motani, R. *et al.* Lunge feeding in early marine reptiles and fast evolution of marine tetrapod feeding guilds. *Scientific Reports* **5**, doi:10.1038/srep08900 (2015).
6. McGowan, C. & Motani, R. *Ichthyopterygia*. Vol. 8 (Verlag Dr. Friedrich Pfeil, 2003).
7. Chen, X. & Cheng, L. A new species of large-sized and long-body ichthyosaur from the Late Triassic Guanling biota, Guizhou, China. *Geological Bulletin of China* **22**, 228-235 (2003).

8. Sander, P. M., Chen, X., Cheng, L. & Wang, X. Short-snouted toothless ichthyosaur from China suggests Late Triassic diversification of suction feeding ichthyosaurs. *PLoS One* **6**, e19480, doi:10.1371/journal.pone.0019480 (2011).

Supplementary Table S3. Data for Fig. 5.

We did not include *Quasianosteosaurus* because it is based on two very fragmentary pieces of rock, one preserving only impression of a part of the cheek, and the other a small part of the snout. Its published diagnosis did not contain any unique apomorphy. Its addition would favor our argument for the taxonomic turnover at the Early/Middle Triassic boundary but only result in unnoticeable changes in Fig. 5.

Genus	Species	CLADE	FirstSubstage	LastSubstage
Cartorhynchus	*lenticarpus*	Ichthyosauriformes	Spathian	Spathian
Chaohusaurus	*chaoxianensis*	Ichthyopterygia	Spathian	Spathian
Chaohusaurus	*geishanensis*	Ichthyopterygia	Spathian	Spathian
Chaohusaurus	sp.	Ichthyopterygia	Spathian	Spathian
Chaohusaurus	*zhangjiawanensis*	Ichthyopterygia	Spathian	Spathian
Corosaurus	*alcovensis*	Sauropterygia	Spathian	Spathian
Eohupehsuchus	*brevicollis*	Hupehsuchia	Spathian	Spathian
Eretmorhipis	*carrolldongi*	Hupehsuchia	Spathian	Spathian
Grippia	*longirostris*	Ichthyopterygia	Spathian	Spathian
Hanosaurus	*hupehensis*	Sauropterygia	Spathian	Spathian
Hupehsuchus	*nanchangensis*	Hupehsuchia	Spathian	Spathian
Isfjordosaurus	*minor*	Ichthyopterygia	Spathian	Spathian
Keichousaurus	*yuananensis*	Sauropterygia	Spathian	Spathian
Kwangsisaurus	*orientalis*	Sauropterygia	Spathian	Spathian
Majiashanosaurus	*discocoracoidis*	Sauropterygia	Spathian	Spathian
Nanchangosaurus	*suni*	Hupehsuchia	Spathian	Spathian
Omphalosaurus	*nettarhynchus*	Omphalosauria	Spathian	Spathian
Omphalosaurus	*nisseri*	Omphalosauria	Spathian	Spathian

Parahupehsuchus	*longus*	Hupehsuchia	Spathian	Spathian
Sclerocormus	*parviceps*	Ichthyosauriformes	Spathian	Spathian
Thaisaurus	*chonglakmanii*	Ichthyopterygia	Spathian	Spathian
Utatsusaurus	*hataii*	Ichthyopterygia	Spathian	Spathian
Agkistrognathus	*campbelli*	Thalattosauriformes	Spathian_question	Longobardian_question
Paralonectes	*merriami*	Thalattosauriformes	Spathian_question	Longobardian_question
Parvinatator	*wapitiensis*	Ichthyopterygia	Spathian_question	Longobardian_question
Gulosaurus	*helmi*	Ichthyopterygia	Spathian_question	Longobardian_question
Cymatosaurus	*fridericianus*	Sauropterygia	Aegean	Aegean
Cymatosaurus	*latifrons*	Sauropterygia	Aegean	Aegean
Cymatosaurus	*minor*	Sauropterygia	Aegean	Aegean
Cymatosaurus	*multidentatus*	Sauropterygia	Aegean	Aegean
Germanosaurus	*schafferi*	Sauropterygia	Aegean	Aegean
Chinchenia	*sungi*	Sauropterygia	Aegean_question	Aegean_question
Sanchiaosaurus	*dengi*	Sauropterygia	Aegean_question	Aegean_question
Thalattosaurus	*borealis*	Thalattosauriformes	Aegean_question	Longobardian_question
Shingyisaurus	*unexpectus*	Sauropterygia	Anisian_question	Anisian_question
Tanystropheus	*conspicuus*	Protorosauria	Anisian_question	Anisian_question
Nothosaurus	*winkelhorsti*	Sauropterygia	Bithynian	Bithynian
Nothosaurus	*winterswjikensis*	Sauropterygia	Bithynian	Bithynian
Palatodonta	*bleekeri*	Sauropterygia	Bithynian	Bithynian
Pararcus	*diepenbroeki*	Sauropterygia	Bithynian	Bithynian
Psephosaurus	*mosis*	Sauropterygia	Bithynian	Bithynian
Phalarodon	*atavus*	Ichthyopterygia	Bithynian	Illyrian
Placodus	*gigas*	Sauropterygia	Bithynian	Fassanian
Atopodentatus	*unicus*	Sauropterygia	Pelsonian	Pelsonian

Diandongosaurus	*acutidentatus*	Sauropterygia	Pelsonian	Pelsonian
Dianopachysaurus	*dingi*	Sauropterygia	Pelsonian	Pelsonian
Dinocephalosaurus	*orientalis*	Protorosauria	Pelsonian	Pelsonian
Largocephalosaurus	*polycarpon*	Saurosphargidae	Pelsonian	Pelsonian
Largocephalosaurus	*qianensis*	Saurosphargidae	Pelsonian	Pelsonian
Lariosaurus	*hongguoensis*	Sauropterygia	Pelsonian	Pelsonian
Mixosaurus	*panxianensis*	Ichthyopterygia	Pelsonian	Pelsonian
Nothosaurus	*yangjuanensis*	Sauropterygia	Pelsonian	Pelsonian
Nothosaurus	*zhangi*	Sauropterygia	Pelsonian	Pelsonian
Phalarodon	*fraasi*	Ichthyopterygia	Pelsonian	Longobardian
Placodus	*inexpectatus*	Sauropterygia	Pelsonian	Pelsonian
Qianosuchus	*mixtus*	Archosauria	Pelsonian	Pelsonian
Sinosaurosphargis	*yunguiensis*	Saurosphargidae	Pelsonian	Pelsonian
Tanystropheus	*haasi*	Protorosauria	Pelsonian	Pelsonian
Tholodus	*schmidi*	Ichthyopterygia	Pelsonian	Pelsonian
Wumengosaurus	*delicatomandibularis*	Sauropterygia	Pelsonian	Pelsonian
Xinminosaurus	*catactes*	Ichthyopterygia	Pelsonian	Pelsonian
Anarosaurus	*heterodontus*	Sauropterygia	Illyrian	Illyrian
Anarosaurus	*pumilio*	Sauropterygia	Illyrian	Illyrian
Augustasaurus	*hagdorni*	Sauropterygia	Illyrian	Illyrian
Besanosaurus	*leptorhynchus*	Ichthyopterygia	Illyrian	Illyrian
Cyamodus	*kuhnschnyderi*	Sauropterygia	Illyrian	Fassanian
Cyamodus	*muensteri*	Sauropterygia	Illyrian	Illyrian
Cyamodus	*rostratus*	Sauropterygia	Illyrian	Illyrian
Cymbospondylus	*buchseri*	Ichthyopterygia	Illyrian	Illyrian
Cymbospondylus	*nichollsi*	Ichthyopterygia	Illyrian	Illyrian

Cymbospondylus	*piscosus*	Ichthyopterygia	Illyrian	Illyrian
Thalattoarchon	*saurophagis*	Ichthyopterygia	Illyrian	Illyrian
Dactylosaurus	*gracilis*	Sauropterygia	Illyrian	Illyrian
Mixosaurus	*cornalianus*	Ichthyopterygia	Illyrian	Illyrian
Mixosaurus	*kuhnschnyderi*	Ichthyopterygia	Illyrian	Illyrian
Nothosaurus	*giganteus*	Sauropterygia	Illyrian	Julian
Nothosaurus	*juvenilis*	Sauropterygia	Illyrian	Illyrian
Nothosaurus	*marchicus*	Sauropterygia	Illyrian	Illyrian
Nothosaurus	*mirabilis*	Sauropterygia	Illyrian	Fassanian
Omphalosaurus	*nevadanus*	Omphalosauria	Illyrian	Illyrian
Phalarodon	*callawayi*	Ichthyopterygia	Illyrian	Illyrian
Phantomasaurus	*neubigi*	Ichthyopterygia	Illyrian	Illyrian
Pistosaurus	*longaevus*	Sauropterygia	Illyrian	Illyrian
Blezingeria	*ichthyospondylus*	Thalattosauriformes	Fassanian	Fassanian
Cyamodus	*hildegardensis*	Sauropterygia	Fassanian	Fassanian
Lariosaurus	*buzzii*	Sauropterygia	Fassanian	Fassanian
Neusticosaurus	*edwardsi*	Sauropterygia	Fassanian	Fassanian
Neusticosaurus	*peyeri*	Sauropterygia	Fassanian	Fassanian
Neusticosaurus	*pusillus*	Sauropterygia	Fassanian	Fassanian
Nothosaurus	*jagisteus*	Sauropterygia	Fassanian	Fassanian
Odoiporosaurus	*Teruzzii*	Sauropterygia	Fassanian	Fassanian
Omphalosaurus	*wolfi*	Omphalosauria	Fassanian	Fassanian
Paraplacodus	*broilii*	Sauropterygia	Fassanian	Fassanian
Serpianosaurus	*mirigiolensis*	Sauropterygia	Fassanian	Fassanian
Simosaurus	*gaillardoti*	Sauropterygia	Fassanian	Julian
*_Psephosaurus_*	*picardi*	Sauropterygia	Illyrian/Fassanian	Illyrian/Fassanian

*_Psephosaurus_*	*sinaiticus*	Sauropterygia	Illyrian/Fassanian	Illyrian/Fassanian
Clarazia	*schinzi*	Thalattosauriformes	Illyrian/Fassanian	Illyrian/Fassanian
Eusaurosphargis	*dalsassoi*	Helveticosauridae	Illyrian/Fassanian	Illyrian/Fassanian
Helveticosaurus	*zollingeri*	Helveticosauridae	Illyrian/Fassanian	Illyrian/Fassanian
Hescheleria	*ruebeli*	Thalattosauriformes	Illyrian/Fassanian	Illyrian/Fassanian
Lariosaurus	*stensioei*	Sauropterygia	Illyrian/Fassanian	Illyrian/Fassanian
Macrocnemus	*bassanii*	Protorosauria	Illyrian/Fassanian	Illyrian/Fassanian
Nothosaurus	*haasi*	Sauropterygia	Illyrian/Fassanian	Illyrian/Fassanian
Nothosaurus	*tchernovi*	Sauropterygia	Illyrian/Fassanian	Illyrian/Fassanian
Tanystropheus	*langobardicus*	Protorosauria	Illyrian/Fassanian	Illyrian/Fassanian
Lariosaurus	*curionii*	Sauropterygia	Ladinian_question	Ladinian_question
Askeptosaurus	*italicus*	Thalattosauriformes	Fassanian	Fassanian
Anshunsaurus	*wushaensis*	Thalattosauriformes	Longobardian	Longobardian
Ceresiosaurus	*calcagnii*	Sauropterygia	Longobardian	Longobardian
Ceresiosaurus	*lanzi*	Sauropterygia	Longobardian	Longobardian
Glyphoderma	*kangi*	Sauropterygia	Longobardian	Longobardian
Keichousaurus	*hui*	Sauropterygia	Longobardian	Longobardian
Lariosaurus	*balsami*	Sauropterygia	Longobardian	Longobardian
Lariosaurus	*valceresii*	Sauropterygia	Longobardian	Longobardian
Lariosaurus	*xingyiensis*	Sauropterygia	Longobardian	Longobardian
Macrocnemus	*fuyuanensis*	Protorosauria	Longobardian	Longobardian
Neusticosaurus	*staubi*	Sauropterygia	Longobardian	Longobardian
Neusticosaurus	*toeplitschi*	Sauropterygia	Longobardian	Longobardian
Nothosaurus	*cymatosauroides*	Sauropterygia	Longobardian	Longobardian
Nothosaurus	*youngi*	Sauropterygia	Longobardian	Longobardian
Psephosaurus	*suevicus*	Sauropterygia	Longobardian	Longobardian

Qianichthyosaurus	*xingyiensis*	Ichthyopterygia	Longobardian	Longobardian
Qianxisaurus	*chajiangensis*	Sauropterygia	Longobardian	Longobardian
Wangosaurus	*brevirostris*	Sauropterygia	Longobardian	Longobardian
Yunguisaurus	*liae*	Sauropterygia	Longobardian	Longobardian
Nothosaurus	*edingerae*	Sauropterygia	Longobardian/Julian	Longobardian/Julian
Anshunsaurus	*huangguoshuensis*	Thalattosauriformes	Julian	Julian
Bobosaurus	*forojuliensis*	Sauropterygia	Julian	Julian
Concavispina	*biseridens*	Thalattosauriformes	Julian	Julian
Guanlingsaurus	*liangae*	Ichthyopterygia	Julian	Julian
Guizhouichthyosaurus	*tangae*	Ichthyopterygia	Julian	Julian
Henodus	*chelyops*	Sauropterygia	Julian	Julian
Miodentosaurus	*brevis*	Thalattosauriformes	Julian	Julian
Protenodontosaurus	*italicus*	Sauropterygia	Julian	Julian
Psephochelys	*polyosteoderma*	Sauropterygia	Julian	Julian
Qianichthyosaurus	*zhoui*	Ichthyopterygia	Julian	Julian
Sinocyamodus	*xinpuensis*	Sauropterygia	Julian	Julian
Xinpusaurus	*bamaolinensis*	Thalattosauriformes	Julian	Julian
Xinpusaurus	*suni*	Thalattosauriformes	Julian	Julian
Placochelys	*placodonta*	Sauropterygia	Julian/Tuvalian	Julian/Tuvalian
Californosaurus	*perrini*	Ichthyopterygia	Tuvalian	Tuvalian
Nectosaurus	*halinus*	Thalattosauriformes	Tuvalian	Tuvalian
Shastasaurus	*pacificus*	Ichthyopterygia	Tuvalian	Tuvalian
Shonisaurus	*popularis*	Ichthyopterygia	Tuvalian	Tuvalian
Thalattosaurus	*alexandrae*	Thalattosauriformes	Tuvalian	Tuvalian
Toretocnemus	*californicus*	Ichthyopterygia	Tuvalian	Tuvalian
Toretocnemus	*zitteli*	Ichthyopterygia	Tuvalian	Tuvalian

Endennasaurus	*acutirostris*	Thalattosauriformes	Alaunian	Alaunian
Macgowania	*janiceps*	Ichthyopterygia	Alaunian	Alaunian
Psephoderma	*alpinum*	Sauropterygia	Alaunian	Rhaetian
Shonisaurus	*sikanniensis*	Ichthyopterygia	Alaunian	Alaunian
Himalayasaurus	*tibetensis*	Ichthyopterygia	Lacian/Alaunian	Lacian/Alaunian
Callawayia	*neoscapularis*	Ichthyopterygia	Lacian	Lacian
Hudsonelpidia	*brevirostris*	Ichthyopterygia	Lacian	Lacian
Sikannisuchus	*huskyi*	Archosauria	Lacian	Lacian
Macroplacus	*raeticus*	Sauropterygia	Rhaetian	Rhaetian
Psephoderma	*anglicum*	Sauropterygia	Rhaetian	Rhaetian

Supplementary Method

Bayesian phylogenetic analysis

We used MrBayes 3.2.6[1] for Bayesian inference of phylogeny approximately following the settings used by Slater (2013)[2]. The character coding was treated as "variable" to account for the lack of autapomorphies and constant cahracteres in the data. Gamma distribution was used to account for the variation in rates among characters. The clock was set as fossilization to take advantage of the fossilized birth-death process model. The fossiltip option was used to treat all taxa as terminal. Independent Gamma Rate (IGR) model was used to treat each branch as having an independent rate drawn from a gamma distribution[3]. The clock rate prior was set as a lognormal distribution around the mean rate that was calculated from the character steps and time duration along the longest stretch of branches in a parsimony tree. Varying this value, however, had minimal effects on the result.

The age of each taxon was calibrated based on the stratigraphic record, derived from the Paleobiology Database and the Geologic Time Scale 2012[4]. Specifically, the prior age range of a taxon was defined as a uniform distribution spanning the geologic substage of its first occurrence, as well as the substage before. Geologic stages were uses instead for Paleozoic taxa, because geologic substages are not officially defined.

Default options were employed for other priors. Outgroup topology was constrained. Each search was based on four runs, each with four chains. The analysis was stopped when the standard deviation of marginal split frequencies reached 0.01, which took about 19 to 22 million generations. Convergence of topology was confirmed in RWTY (https://github.com/danlwarren/RWTY). The first 25% of the data were discarded as burn-in during posterior calculations of parameters and consensus topology.

1 Ronquist, F. *et al.* MrBayes 3.2: Efficient Bayesian Phylogenetic Inference and Model Choice Across a Large Model Space. *Systematic Biol* **61**, 539-542, doi:10.1093/sysbio/sys029 (2012).

2 Slater, G. J. Phylogenetic evidence for a shift in the mode of mammalian body size evolution at the Cretaceous-Palaeogene boundary. *Methods Ecol Evol* **4**, 734-744, doi:10.1111/2041-210x.12084 (2013).

3 Lepage, T., Bryant, D., Philippe, H. & Lartillot, N. A general comparison of relaxed molecular clock models. *Mol Biol Evol* **24**, 2669-2680, doi:10.1093/molbev/msm193 (2007).

4 Gradstein, F. M., Ogg, J. G., Schmitz, M. D. & Ogg, G. M. *The Geologic Time Scale 2012*. (Elsevier, 2012).

Supplementary NEX File Data1 -- NEX file data for the phylogenetic analysis to show the position of the new taxon among Ichthyosauriformes

#NEXUS
BEGIN TAXA;
TITLE Taxa;
DIMENSIONS NTAX=73;
TAXLABELS
Petrolacosaurus Claudiosaurus Thadeosaurus Hovasaurus Wumengosaurus Nanchangosaurus Eohupehsuchus Hupehsuchus Parahupehsuchus Eretmorhipis Sclerocormus Cartorhynchus Chaohusaurus_geishanensis Chaohusaurus_chaoxianensis Chaohusaurus_zhangjiawanensis Utatsusaurus Grippia Gulosaurus Parvinatator Mixosaurus_panxianensis Mixosaurus_cornalianus Mixosaurus_kuhnschnyderi Phalarodon_atavus Phalarodon_fraasi Phalarodon_callawayi Cymbospondylus_piscosus Cymbospondylus_buchseri Cymbospondylus_nichollsi Xinminosaurus Besanosaurus Guizhouichthyosaurus Callawayia_wolonggangensis Guanlingsaurus Shastasaurus Shonisaurus_popularis Shonisaurus_sikanniensis Californosaurus Toretocnemus Qianichthyosaurus_zhoui Qianichthyosaurus_xingyiensis Callawayia_neoscapularis Hudsonelpidia Macgowania Suevoleviathan Temnodontosaurus Leptonectes_tenuirostris Leptonectes_moorei Leptonectes_solei Excalibosaurus Eurhinosaurus Ichthyosaurus Stenopterygius Aegirosaurus Acamptonectes Maiaspondylus Arthropterygius Athabascasaurus Sveltonectes Brachypterygius Caypullisaurus Malawania Mollesaurus Leninia Chacaicosaurus_ Hauffiopteryx Platypterygius_australis Platypterygius_americanus Cryopterygius Ophthalmosaurus_icenicus Ophthalmosaurus_natans Thalattoarchon Palvennia Sisteronia
;
END;
BEGIN CHARACTERS;
TITLE Character_Matrix;
DIMENSIONS NCHAR=195;
FORMAT DATATYPE = STANDARD GAP = - MISSING = ? SYMBOLS = " 0 1 2 3 4 5 6";
CHARSTATELABELS
1 Premaxilla_dorsal_process / long short, 2 Premaxilla_ventral_process / long short, 3 Maxilla_anterior_process / reduced extending_anteriorly_as_far_as_nasal_or_further_anteriorly, 4 Maxilla_dorsal_lamina / absent present, 5 Maxilla_prefrontal_contact / absent present, 6 Maxilla_external_naris_contact / present absent, 7 Maxilla_longer_than_premaxilla / TRUE FALSE, 8 External_naris_orientation / lateral dorsal, 9 Shallow_groove_anterior_to_the_exn / absent present, 10 Narial_shelf / absent present, 11 Nasal_anteriorly_extending_beyond_exn / FALSE TRUE, 12 Nasal_parietal_contact_lateral_to_frontal / absent present, 13 Nasal_postfrontal_contact / no_contact 'contact extensive, posterior extension of nasal separates frontal from prefrontal in dorsal view' eliminated_by_prefrontal_medially_extension, 14 Descending_process_of_the_nasal_on_the_dorsal_border_of_the_nares / absent present, 15 Nasals_rostrally_reaching_snout_tip / FALSE TRUE, 16 Processus_narialis_of_prefrontal /

absent present, 17 Supraorbital_crest_on_prefrontal_and_postfrontal / absent present, 18 'Prefrontal-postfrontal contact' / absent present, 19 Prefrontal_exposure_in_UTF / absent present, 20 Anterior_orbital_margin / of_regular_rounded_shape irregular, 21 Postfrontal_medial_extension / over_the_anteriormost_margin_of_utf not_over_the_anteriormost_margin_of_utf, 22 'Supratemporal antero-medial extension' / short long, 23 'Supratemporal-postorbital contact' / absent present, 24 'Supertemporal / squamosal relative size' / equal_or_st_smaller st_clearly_larger, 25 Sagittal_eminence / absent 'present but small, involving only the parietal' 'present and large, involving the parietal, frontal and nasal', 26 Frontal_dorsal_exposure / clearly_present nearly_absent, 27 Frontal_participation_in_utf / absent present, 28 Squamosal_triangular_shape / FALSE TRUE squamosal_absent, 29 Squamosal_participation_in_utf / present absent squamosal_absent, 30 'Squamosal-quadrate articulation' / present absent, 31 'Postorbital postero-dorsal corner' / 'narrow, giving triradiate shape' broad_and_triangular absent_or_round, 32 Postorbital_participation_in_utf / present absent, 33 Jugal_anterior_margin / 'tapering, between lacrimal and maxilla' 'broad and fan-like, covering ma', 34 'Jugal/quadratojugal lateral contact' / present absent, 35 Lower_temporal_arch_between_jugal_and_quadratojugal / present lost, 36 Quadratojugal / longer_than_tall taller_than_long., 37 Quadratojugal_exposure / quadratojugal_small extensive 'small, largely covered by squamosal and postorbital', 38 Parietal_ridge / absent present, 39 Parietal_supratemporal_process / short long, 40 Parietal_anterior_processes / 'contacting each other anteriorly, eliminating frontal from pineal foramen' 'narrowly separated anteriorly, forming parietal fork, and frontal dorsally visible along the pineal foramen' 'widely open, resulting in absence of clear fork', 41 'Parietal-frontal suture inter-digitation' / absent present, 42 Anterior_terrace_of_utf / absent present_but_small present_and_large, 43 Basioccipital_peg / clearly_present absent_or_extremely_reduced, 44 Basioccipital_extracondylar_area / wide reduced_to_a_narrow_band_of_concavity, 45 'Basioccipital/atlas articulation convexity' / flat_or_anterior posterior, 46 Ventral_notch_in_the_extracondylar_area_of_the_basioccipital / present absent, 47 'Pterygoid, transverse flange' / 'antero-lateral' not_well_defined 'postero-lateral', 48 Basipterygoid_processes / 'short, giving basisphenoid a square outline in dorsal view' 'markedly expanded laterally, being wing-like, giving basisphenoid a marked pentagonal shape in dorsal view', 49 Interpterygoid_vacuity / present absent_or_extremely_reduced, 50 Ectopterygoid / present absent, 51 Shape_of_the_paroccipital_process_of_the_opisthotic / short_and_robust elongated_and_slender, 52 Stapes_proximal_head / 'slender, much smaller than opisthotic proximal head' 'massive, as large or larger than opisthotic', 53 Cheek_orientation / lateral posterior, 54 Overbite / absent_or_very_slightly present, 55 Prenarial_snout_longer_than_the_postorbital_skull / FALSE TRUE, 56 Snout_extremely_slender / no yes, 57 'Snout, constriction' / absent present, 58 Snout_flattened / FALSE TRUE, 59 'Scleral ring extensively ossified, filling or almost filling the orbit' / FALSE TRUE, 60 Pineal_foramen_posterior_to_or_between_orbits / posterior between, 61 Angular_lateral_exposure_at_its_maximum_depth / 'semi-equal to surangular' clearly_shallower_than_surangular much_deeper_than_surangular, 62 Coronoid_region / slightly_elevated_or_high flat, 63 Root_striations / present absent, 64 Plicidentine / absent at_least_partly_present, 65 Bony_fixation_of_teeth / present absent, 66 Tooth_horizontal_section / circular 'disto-medially compressed' lateral_compressed, 67

Tooth_size_relative_to_the_skull_width / over_0.1 below_0.05, 68 Dentigerous_region_in_adult / complete largely_reduced edentulous, 69 Dental_groove / present_throughout_jaw_margin only_present_anteriorly absent, 70 Anterior_sockets / present absent, 71 Maxilla_multiple_tooth_row / absent present, 72 Dentary_labial_shelf / present absent, 73 Posterior_tooth_crown / conical rounded flat, 74 Tooth_crown_surface_of_at_least_one_maxillary_tooth_with_mesiodistal_ridge / FALSE TRUE, 75 Ossified_sternum / absent present, 76 Ossified_cleithrum / present absent, 77 Clavicle_orientation_at_proximal_end / oblique_to_sagittal_plane transverse, 78 Clavicle_scapular_process_length_distal_to_clavicular_main_body / short long, 79 Interclavicle_anterior_process_separating_clavicles / present absent, 80 Interclavicle_posterior_process / 'rod-like' triangular absent, 81 Scapular_blade_shaft / absent present_at_least_proximally, 82 Scapula_anterior_flange / complete emarginated absent, 83 'Scapula antero-proximal extension toward clavicle' / absent present, 84 rominent_acromion_process_of_scapula / absent present, 85 Scapula_posterior_extension / present absent, 86 Scapular_axis_and_glenoid_facet_orientations / nearly_parallel at_60_degrees_or_more, 87 Coracoid_facet_on_scapula / fused_scapulocoracoid absent equal_or_smaller_than_glenoid_facet_of_scapula twice_as_large_as_glenoid_facet, 88 Coracoid_parasagittal_length_vs._transverse_width / 'semi-equal or longer than wide' clearly_wider_than_long, 89 Coracoid_foramen / present absent, 90 Coracoid_anterior_notch_or_concavity / absent present, 91 Coracoid_posterior_notch_or_concavity / absent present, 92 Intercoracoid_facet / 'short, medial margin round shaped' 'long, medial margin relatively straight', 93 Humerus_anterior_flange / absent present_and_complete 'present but reduced proximally, leaving leading edge tuberosity', 94 'Plate-like dorsal ridge on humerus' / absent present, 95 Protruding_triangular_deltopectoral_crest_on_humerus / present_but_small 'present and very large, bordered by concave areas', 96 Humerus_distal_proximal_width_ratio / nearly_equal distal_wider, 97 Humerus_with_posterodistally_deflected_ulnar_facet_and_distally_facing_radial_facet / ulnar_facet_contains_convexity_in_lateral_view absent present, 98 Humerus_distal_articular_facets / not_terminal radial_facet_larger_than_ulna_facet two_facet_nearly_equal three_facets, 99 Humerus_anterodistal_facet_for_accessory_zeugopodial_element_anterior_to_radius / absent present, 100 'Humerus/intermedium contact' / absent present, 101 Radius_peripheral_shaft / complete_or_nearly_complete notch_or_absent, 102 Radius_contiguous_shaft / about_half_or_more_of_radial_length notch_or_absent, 103 Ulna_peripheral_shaft / complete_or_nearly_complete notch_or_absent, 104 Ulna_contiguous_shaft / complete_or_nearly_complete notch_or_absent, 105 Shape_of_the_posterior_surface_of_the_ulna / radius_not_discoidal rounded_or_straight_and_nearly_as_thick_as_the_rest_of_the_element 'concave with a thin, blade-like margin', 106 'Radius/ulna relative size' / nearly_equal radius_much_larger_than_ulna ulna_larger_than_radius, 107 'Radio-ulnar foramen' / present absent, 108 'Radiale, anterior notch' / absent present, 109 Manual_pisiform / present absent, 110 'Manual pisiform 2 (neomorph)' / absent present, 111 Intermedium / longer_than_wide as_wide_as_long_or_wider_than_long, 112 Proximal_carpals / packed 'small, round and separated', 113 Distal_carpal_1 / ossified unossified absent, 114 Distal_carpal_2 / ossified

unossified absent, 115 Manual_centralia / present absent, 116 Mc_I_peripheral_shaft / complete notch_or_largely_reduced absent mc_1_not_ossified, 117 Mc_III_shaft / present absent, 118 Mc_V / present not_ossified, 119 Manual_digit_2_distal_elements_peripheral_shaft / complete 'notch or absent;', 120 Forelimb_hyperphalangy_with_more_than_five_phalanges_ossified_in_longest_digit / absent present, 121 'Notching of anterior facet of leading edge elements of forefin in adults, except radiale' / elements_not_discoidal present absent, 122 First_preaxial_accessory_digits_on_forelimb / absent present, 123 Postaxial_accessory_digit_on_forelimb / absent only_one more_than_one, 124 'Proximal manual phalanges proximo-distal packing' / 'well-packed' not_packed, 125 'Manual digit S4-5' / absent present, 126 'Propodial + epipodial versus manus length' / 'propodial + epipodial longer' manus_longer, 127 'Forelimb/hindlimb ratio' / nearly_equal_or_hind_longer forelimb_longer_but_less_than_twice_as_hindlimb forelimb_longer_twice_as_much_as_hindlimb, 128 Forelimb_Zeugopodium_flattened / FALSE TRUE, 129 Manual_anterior_sesamoid / absent present, 130 Second_preaxial_accessory_digit_of_forelimb / absent present, 131 Delayed_mesopodial_ossification / present absent, 132 Forelimb_hypophalangy_with_less_than_five_digits_ossified_in_longest_digit / absent present, 133 'Carpus elongated, as long as the more distal forelimb part or longer' / FALSE TRUE, 134 Extra_preaxial_carpal_proximally / absent present, 135 Interdigital_separation / present absent, 136 Iliac_blade_shape / with_thick_shaft 'plate-like' narrow_and_styloidal, 137 'Iliac antero-medial prominence' / absent present, 138 'Ilium-pubis relative proximo-distal length' / 'semi-equal' ilium_clearly_shorter, 139 'Pubis, styloidal or plate-like' / 'plate-like' styloidal, 140 Pubis_obturator_foramen / completely_enclosed_in_pubis mostly_in_pubis_but_open_on_one_side part_of_obturator_fossa, 141 Pubis_and_ischium_median_symphysis / present not_well_defined, 142 Pubis_and_ischium_fused_in_adult / complete absent present_only_medially present_medially_and_distally, 143 Pubis_ischium_relative_size / nearly_equal_or_ischium_slightly_larger pubis_twice_as_large_as_ischium, 144 'Ischium, styloidal or plate-like' / 'plate-like, longer sagittally than wide transversely' 'plate-like, wider transversely than long sagittally' styloidal, 145 Thyroid_fenestra / absent one_median_opening 'two openings, being medially separated', 146 'Femur strongly constricted medially, forming a slender shaft region, proximal width remarkably larger than medial width' / present absent, 147 'Femur antero-distal expansion, forming a distinctive structure at the distal end' / absent present., 148 'Prominent, ridge-like dorsal and ventral processes demarcated from the head of the femur and extending up to mid-shaft' / absent present, 149 Wide_distal_femur_blade / 'absent, the proximal and distal extremity of the femur being sub-equal in dorsal view' present, 150 Femur_distal_facets / two three, 151 'Femur/astragalus contact' / absent present, 152 Femur_anterodistal_facet_for_accessory_zeugopodial_element_anterior_to_tibia / absent present, 153 Tibia_contiguous_shaft / complete_or_nearly_complete notch_or_absent, 154 Tibia_peripheral_shaft / complete_or_nearly_complete notch_or_absent, 155 'Tibia antero-proximal end nearly rectangular, forming a deep notch on the anterior margin' / absent present, 156 Fibula_posterior_extent / 'not fixed, fibula being mobile relative to femur' much_posterior_to_femur about_the_same_level_as_femur, 157 Fibula_contiguous_margin / concave nearly_straight_or_convex, 158 Fibula_posterior_flange / absent present, 159

Spatium_interosseum_between_tibia_and_fibula_size / in_contact_or_closely_placed_with_each_other widely_separated_from_each_other, 160 Spatium_interosseum_between_tibia_and_fibula_presence / present absent, 161 Hind_fin_leading_edge_element_in_adults / elements_not_discoidal notched straight, 162 Postaxial_accessory_digit_in_hind_limb / absent present, 163 Pes_digit_1 / present absent, 164 Dt_2_in_line_with_dt_3_and_4 / TRUE 'false, dt 2 in line with calcaneum', 165 'Atlas/axis fusion' / absent present, 166 Presacral_count / less_than_30 'between 40-52' 55_or_more, 167 Preflexual_caudal_count / tail_stem_not_well_defined '1/2 of the prepelvic count' '2/3 of the prepelvic count or more', 168 Posterior_dorsal_centra_shape / cylindrical discoidal, 169 'Posterior dorsal/anterior caudal centra degree of shortening' / 3.5_times_or_less_as_high_as_long four_times_or_more_as_high_as_long, 170 Cervical_bicipital_rib_facet / absent present, 171 Rib_articulation_in_dorsal_region / predominantly_unicapitate exclusively_bicapitate, 172 'Antero-dorsal rib facets' / confluent_with_anterior_facet_in_at_least_near_pelvic_girdle not_confluent_in_any_of_the_centra, 173 'Posterior-dorsal bicipital rib facet' / absent 'present, might be resulted from the split of diapophysis', 174 Last_caudal_rib_facet_reaching_the_caudal_peak_area / FALSE TRUE, 175 'Rib with broadened mid-shaft rostro-caudally' / absent present, 176 Sacral_ribs / 'at least two, distinguishable' absent, 177 Anterior_dorsal_neural_spine / normal narrow, 178 'Neural spines of atlas-axis' / 'functionally separate, never fused' 'completely overlapping, may be fused', 179 Neural_spine_anticlination_in_tail / absent present, 180 'Dorsal neural arch, transverse process' / present absent, 181 Second_dorsal_segment_on_dorsal_neural_spines / absent present, 182 Anterior_dorsal_neural_spines_with_thickened_central_axis_and_rostral_and_caudal_median_ridges_or_flanges / absent present, 183 'Caudal peak with curved vertebral column, near anticlination of neural spine' / absent present, 184 'Anterior caudal vertebral size about 1/2 of the largest dorsal vertebrae or less' / FALSE TRUE, 185 'Mid-caudal vertebrae height change' / gradually_decrease increase sudden_decrease, 186 'Pre-flexural wedge-shaped centra' / absent present, 187 Tail_proportion_relative_to_the_rest_of_the_body / as_long_or_longer_than_the_rest_of_the_body distinctly_shorter, 188 Lunate_tailfin_suggested_by_tailbend / absent well_developed_lunate_tailfin, 189 'Chevrons in caudal-peak region' / present absent, 190 'Fluke vertebrae, laterally flattened and packed' / absent present, 191 Posterior_gastralia_immediately_craniad_of_pelvic_girdle / present absent, 192 Flat_gastral_elements_with_caudad_one_outlying_the_craniad_one / absent present, 193 Dermal_ossicle / absent present, 194 Dermal_armor_above_caudal_vertebrae_with_hemal_arches / absent present, 195 'Dermal armor above dorsal neural spines, first layer' / absent present ;

MATRIX

Petrolacosaurus

000000000000000000000000000000000000000000?00?0?00??000000?000?0000000000000000
00000000000000000000000000000000000000000000000000000000000000000000000000000000
00000000000000000000000000000000?0000

Claudiosaurus

000100010000??00000000?000000000?101001010??0?0?10??0000000000?000000?0100010000
00??0000000000?1000000000000001000000000000000000000000000?0000100100000000000000

0000000000?0?00000?00000000?0?000000

Thadeosaurus ??????0???
???????????0????????????110??000000000000000??0000000000000000000000000000000000
000000000100100000000000000000000?0000?0000000?00000000?0?0?0000

Hovasaurus ?????????????????0??00?000000000??0?0000?0?01?1
?0?0???0????0?0?????????????100?1000000000000000001?00000000000000000000000000000
0000000000001100000000000000000000000000010?00000?0001000000?000000

Wumengosaurus
00110010100000000000???000000000101000010??0?1?010?001010100??0??100?0110010001
12100020111000000200000000001001111000000000001000000000100011000000000000000000
0200??100000??0000000000000000?000000

Nanchangosaurus
0??100?????0010000??000?000000000?010?1000??????????0????1?0???????2????????????????
???0100?10?0?20?0000000????????????????????1????0????????????????????????????????1?0?0
0?0?000??010?????????1101

Eohupehsuchus
0111001000000000?1010000000000000001????0000??????????0?1001?0?0?????2????????00?000
0010?0000?10?01200000000001000001000??000????1001?00000?0??????10010?000010000???
??1?0?0?01?0?0???10????????01111

Hupehsuchus 01?1?0?00000000?0(0
1)?10???0?0?0??0???1?000?0??0?2?0???0010010000?????2????????00??00??0020000010?0120
000000000100000100000000000111001000010000?1001100100001010000200001000?00001?00
001(0 1)0000?0?001111

Parahupehsuchus ???
???????1???????????????????????????????????1??0120000000000010000010000001000011001001
0??????????10?000000010000000?1?0??00??100?0?10????????01111

Eretmorhipis ???
???1??11200000000000000000100000010000111010010
00001?100110?00000001000000000?1?0??000?100?00100????0??01111

Sclerocormus
00111010001001100100111000000?000????01200??????????0000101100????????????01000000
0011?????????????????1?1???10111101001010010111000010100001110001001000000010000200
0?0200?0??0100101010000000001100

Cartorhynchus
110100000010001000000000?00000?00?1010010?1????????0?0000101100?????2???1???100000
0000???????20?0110010100000101111????10000101110000101001???????1001?000001000020
?0?0?0?00???100??100????????00000

Chaohusaurus_geishanensis ??0???1???1??000?1?0??0????0??1?00010?????????????
??10111011111??110??1110?11???00?0002?????10?0110000000000101010110000000001?10001
00??????????????01?00???????020??????0?0???0??0?100????0????0000

Chaohusaurus_chaoxianensis
11010010001000000100000100000110000100000010?0?1?01??10111011111??1100?0110011112
0000002011001 0?111000000000001011110100000001011100010010000111001100100000010000
2000?1100000000000110010000??100000

Chaohusaurus_zhangjiawanensis ???????????00????1??00??000?0??0?????000?1??0?????
????1???1???????????????01?1??000000??????10?0110000000000?01?10??00??0??????????????
00?00?100?10010?0000?00?0?????110?00?0?100?11001?0?????00000

Utatsusaurus
011100110010000000011001000011100110101001????0?11??0010101111??01100001000101000
00000101110100001000000000000010001100000000000100100010000001001100100000010000
0000011000?000000?1100100000?100000

Grippia
011100?1001000?00001101100000?1001101010?1????0?????0?101??111?10110001110?1????00
00001011101001010000000000001000120000000101?10010001???00?????1001?????????????00
00??001??1???0??1???????????000??

Gulosaurus
00?1001100?0000000?10011000?1??0?????01001????0?????00101011?1?1??10??1?10??????000
0101011101?0?010000000000000000001000??0001???1001?001?????????????????????????????
?0010?0?0?0??100?????????0000

Parvinatator ???1?0?000?????0?0?0??11???011100?1???????????????
????1?10?110?1?000?00100??????????????????????0100000000000000001100010000??10010
001??

Mixosaurus_panxianensis
1111101000100000110010112000112?011?110102??????????0010101111??0?0000011001111100
00001011101001010010100000001100012000100000111001000110000011011001000000100000
000?111010?1?011?11001010000100000

Mixosaurus_cornalianus
1111101000100000110 01?1120??1121000?110102??1???11??0010101111??00100?01100111110
000001011101001010000100000011000120001000001110010001 1?000011011001000000010000
0000?111010011011?11001010000100000

Mixosaurus_kuhnschnyderi
1???1?1??01????11?010??20?????1?00??101?2????????????01?101111???200000110?????10000
001011?01001010?1010000?????00?????????????????1??0????????????????????????????????????
00????1???????1????1?0???

Phalarodon_atavus
111110?0011000?011001011200011210000110102????2?11??0?101??1??110200{1
2}00?00?????1?00000??????1001?30000100000000100012000100000??1001???1??????????10?0
0??????00??000??1?1?????????1?1???1???0?0?0????

Phalarodon_fraasi
11?110?0011????111?01?????????2????????????????????????1?1?1?1??1?200100021?1??????00?
??0????1?0101??0010?0?00100001????????0???10010001?0???011??00?????000100????00????
??????0???11001??????1??000

Phalarodon_callawayi
1111101101 1??00011?0??1120????2100011????2??????????0010101?1????0?0110021??????????
??1????????????????????????0????????????????????????1???00011???0?1000000100000000???0
00?????10?????????????????

Cymbospondylus_piscosus
011100100010100001000111101011100000101101??0?2011??0010101110?100002000000 1?0??

0100001000101000010?00100000???1???????????????1???????10000110011001000000010000??
??021101000?010?1100100000?100000

Cymbospondylus_buchseri ????0?????1???0?0100??1??0????110??????1?????????
???0010??1?10????0???010001?0??0100001?001?1?00010?00100000??11??????1????????1????
?????????????????????????????????2?10?000?0?0??100?????????00?0

Cymbospondylus_nichollsi ???100?0??101??00100111?101?1?1001101010?1??0?2?
1???0?????1110????????????0100??????????0010???
??????????????????????????????0??1?1000???0??????????????????

Xinminosaurus ??????1??
??0?1?101??0?1?2?01011200100120000000?1001?10010100001000001010111100000000011100
10001100?011???100100000001000000?0?2110?0???010?110010000001?0000

Besanosaurus ???11?1???1?????01?0????????????0???????????????
????01110???1?1?0?0??0100??????020000201110100001001111010 1???????1?112????1??????
???100011100110000000001000??????2?10100??010??100????0?0100000

Guizhouichthyosaurus 01110010{0
1}010100001000?01100011210??11??101??????????0010101111?1?000000100011110020001 21
111010000100111111010102013101120010111001000110001110011000000000100001010?2?1
010?0?010?11001???0001?0000

Callawayia_wolonggangensis 011100101010100001?000011010112{0
1}00012011?1????2?1???001{0
1}101111?1?000000100?????0????0??011101?0001001?11111?101????31011100101??????0?1??
????????????????????????????2?10?0?0??????????????????0???

Guanlingsaurus 011110100010100001?000111010112{0
1}0????01001??????????0010101111?????2??????011????20001?1111?10000100111111010112
0131110200101010011101100021100110000000001010010100211 0?00?1010?110010000011000
00

Shastasaurus ????0?1???100???01?0000110100?2100?1101101?01?2
011??0?1?????11?1?0????????011???12000121111110000100 11?11111???0??1?????1??????1001
????1000111001100000000010000???0???101?????1??????1????????????

Shonisaurus_popularis ??????1?????1???????????1??????????????1???01???????
00??????11?1?0?1200???????101200012111111000010010101100???????311111????111???????
10?011100110000000?10100??1??2?101??0?010??10?1??????100000

Shonisaurus_sikanniensis ??????????1??
??0???111???0?12001?0?????0120?0???111?1000010010101 10???????????????????????????????
?????????00000????01?0?????2?1010?0???0?????????????10?0??

Californosaurus ??
?????????????????????????????2000030110?10000200101010011 01???1????????1???1001???11
10021100110000000001 10000?????1210?01?1010?1100112????1?0600

Toretocnemus ????????????????????????????????0?????????????????
??001?1?1?10?110?0010100???????????????0011?0?12001010100110102013111110000 1?10010
0012000011001010100011120000 1011???10?0?1??1???1?0?????????????

Qianichthyosaurus_zhoui 1101001000100000001?0??1110?1111{0
1}0???2????0?01???????001010111001?0?0??010001111??2000??011011000120010101001 1010
20131111101001110010001200001100101010000112000020 11?111010?11010?110011210?1100

000

Qianichthyosaurus_xingyiensis
10?100?00??0000011?0??1??0??????0????????????????????010101?1??????00?0?00???????200
00?0??0?10001100101010001 00????3111110000 1?????000120?011101?01010?00?120000?????1
1100001?0?0?110010210?1?00000

Callawayia_neoscapularis
11???0100010 1?0?01?00?01101?1121?????11101?01?2?????001010?111?110000001000 1??????
0?0??1111010001100111011010010 20131?111001011100100012 0?021100110010001{0
1}1210001?10022101000?0101?100?12?0??1?0000

Hudsonelpidia ???????????????????0??????????????????????????????
?001010??????????????????????????????????200112001111 1000??10??131011?01001?10010001
2101?1101?1001?001011????10?????????????0?1??100????????10000

Macgowania
10000110001??00?01?0???????1??210??11???0???????????001010111001?0000?01000111??121
?103?????2??0120011111001101020131011101001?10010001????????????????????????????????
???????0?0??100?????????0000

Suevoleviathan 10000{0
1}1000101000 01?0??0110?1?12100111101?0??????????001010111101?0000?01000111??121010
3?110120?11200111110101010201310112010{0
1}111001000111012110121001000110210011111 0121?1?111010??1001?2?01?110000

Temnodontosaurus 10100110001110000110000111011121001111{0
1}10000102001000010101101011000010100011110 12?010301101{0
2}001120011111010101020131111 10{0 1}0011100100012 0?121{1
2}0121101000110210101011112 10101?10101?10011210001?0000

Leptonectes_tenuirostris
1000?110?0111000010000?1010111210011110 21010??1?01??1011101??0111010010100011 1101
2101030110100011200111110{0
1}0101020131011200001110010001200121102200010001102101 11011?121010111010??100112
10??110000

Leptonectes_moorei 1000?110?01{0
1}10000100???100??112100110?011???????????1011101 12011?0?001010001111012?010301101
0001120011111010101020131011200001?10010001?????11?????0??00????????????????0?0???0
????100?????????00?0

Leptonectes_solei
10?0?110?01?1???0?00???111???12?????????2??????????????01110????1????????100011????21?1
03011010??1?2??1111?0?010112 0131?10?0010??10011101200121101 2000??001102101???11?1
?1??????0?0??100????0??1?0000

Excalibosaurus
10?00110001??00?01?0???1?1???12?0????????0??1???????1111101101?1101001010001111 0121
1103?1101200112001111101110102013111110 1001?1001000120???11?1?100100011 02101110?1
11?1?1??1?010??10011??000110000

Eurhinosaurus
100000100010?00001?0000101?1?12?0011210??0?01?????0011111011?1011010010100011 1101
2101030110?20?1120011111011001020131111101001110010001200121101210010001102 10111

0111121010?1?0100?100112?000110000

Ichthyosaurus 1000011000102000011000110000{1
2}2121001121021000102001000010101111011000010100011110121010301111000012001111 10
10{0 1}01020131011201101210010001200121{1 2}022100000011021001101111?101111101{0
1}1{0 1}1001121110110000

Stenopterygius 10000{0 1}10001{0
1}200001100011100111210011210210?01020010100101011111110000101000111101211103011
010001120011111011001020131011 10{0 1}1{0
1}121001000120112130221 0?000011021001101111211111110100{0 1}1(0 1)0112111111 0000

Aegirosaurus
10000110001011011 1?00?01?0111121101?2??2?0??????????0111011201???00?0?0?00?1?0???
????0100?01?1130111111210000020131011211001111110001 20?1213?1210?000011021001211 1
?121??????0?0??1001???11?1?0000

Acamptonectes ???0?????????1??????????????????????0?????111?1?
?11?0?0?0????11?0??0????0??????12111020110101012310??112?1?0????????????1????????????
??????????????????????????????1??10???1?0?0???00??????1?????0

Maiaspondylus ?10001?0????????????????????????0??????????????0?
?????????????01?0?00?0?00??????????????????01?01301??111?1?00??????10?????0??????????1
????????????????????????????????1??111?0?????00?????????????

Arthropterygius ???110?0???
??????????1?????????????????????????0110101002310111110 10??1?????????21??????1????????
????????100000??????????????1??111?1????????00??????1??????

Athabascasaurus
1111?0?00010?0??11?00?010100112 11??01002?0?11??????10??0?011??11??0?0?0?00???1?????
???20?121302210?????????????????11?10?
????0?00??00????????????00

Sveltonectes
1?000110001011?111?0????001???2?1?1??01????11120???1?01010??0111?0?0??0?00?????0121
110301101011012001 1????100?1020131011211001?110?0?01???1213012101000011021001211 1
?1210???1?0?0??1001?2???1????00

Brachypterygius
11100110?????1???1?0???1???????1111??????11111?1????0010101?0?1110000?0?00?????????1
????????01?0130111111 0100?102013101121100 1?11010001????????????????????????????????1
???????????1???????????00??

Caypullisaurus 00100{0
1}100?1??100???0??11???0?12???111??????1?????????01010 1101?????1?????????11012111030
?1??0110131011111010101020131111212001?11110001?????????10100001102100121?11??1??
????0?0??100?????????0000

Malawania ???
???????????????????????????11??121010??????2?0?120011111010?01????31011100001???????0
1?????????????????????????????1??1???0??0?0???00?????????????0

Mollesaurus
10?000?0????11?011?0???????0????1?112?????1110?10?010??????????1???00?0?00???1???????
???1??10???1

?0???????????????????

Leninia ???00??0????11?111?00?01?00?111110???01110?110?
?????0???????1??
???

Chacaicosaurus_ ??????10??????????????????????????????????1011????
???011101?????????????????????????????????1???20011111011101010131011100001?100?0?0
1??????????1??????????????????1????1?????????????????????????

Hauffiopteryx ??0000100010000 1?1?00001?0011021011?1?02??001??
?????101110111?????00??0?00?????0120010?0110?1?1112001111011101020131 01?100001?10
011001??1121202210?10001102101 1??111121?11?1?0100?100???????110000

Platypterygius_australis
10111010001011001100101?10122?2110111102000111210101001010112011100001 0?00??????1
2111030110121101310111110100010201311112 12001?10111001???121302?1010101110110012
{0 1}1111?101111?0?00??001??11?1??0000

Platypterygius_americanus
101001100010?10011?0??0??0?22?21?0111??????????????10010101101?1??00010?00??????121
110??????21101300111110100010 20?31??????????10?1?001???121302?1011100?????????????1?
?1011?1?0?0???00????????????00

Cryopterygius
1011001000???1?0?1?0????????????1011????????????????0010?01?20?1?000??0?000111?01211
102011012?1022001111?0101010201310112 11001?10011?0120112120221010000110?10?1????
1121?11?1?010??100??2?1?1????00

Ophthalmosaurus_icenicus
110001100010110011?000110001112110102102001110 2{0
1}01110010101100111 0?0010?000111101211103011012110231011112010001020131011211001
210011?0120112130221010000110210012011 1121{0 1}11111010{0 1}?10011211111?0000

Ophthalmosaurus_natans
110000100010110??1?0001?100110211011 2?0200111?2101??00101011?011?0000?0?000111101
21110301101 21?113101111?01?001020?310?????0???1??11001??????????10?111011021001201
11??1?11?1?0?01?100??????????000

Thalattoarchon ????0??????0???0?1?0111?101?10100????00201???????
???0?????1??001?000????00??1
0????????10?1???????????????????101011?0?0??1000????????????

Palvennia ??0?0?10??101?0??1?000??000????1??????020?1?11??
?????010101?0??1??00??0?00????????????????????????31???????????????????????????????????
??????????????????????????????1????????????????????????????00

Sisteronia ??1??111?0??0
???????????11?0???????0???????12011??????????1??300????????????????????????????????????
????????????????????????????????11????????????????????????

;

END;

begin PAUP;

log file = SI_D1.paup.log replace = Yes start;

SET AUTOCLOSE = YES;

```
SET INCREASE =    NO;
SET MAXTREES =    30000;
SET CRITERION =    PARSIMONY;
SET WARNTREE =    NO;
SET WARNTSAVE =    NO;
SET WARNROOT = NO;
SET WARNREDEF = NO;
SET WARNRESET = NO;
        assume ancstates =    standard;
        ctype unord :    all;
    constraint younginiform ( monophyly ) = ( ( 3 , 4 ) );
        DEFAULTS CONTREE /    STRICT MAJRULE LE50 INDICES Outroot =    paraphyl File
=    consense.tr;
        DEFAULTS HSEARCH ADDSEQ =    RANDOM NREPS =    100 SWAP =    TBR HOLD =
50 ENFORCE =    NO;
        DEFAULTS BANDB ADDSEQ =    FURTHEST ENFORCE =    NO;
        DEFAULTS BOOTSTRAP NREPS =    1000 CONLEVEL =    50 METHOD =
HEURISTIC;
        DEFAULTS DESCRIBE ChgList =    YES;
        export file =    SI_D1.tnt.nex format =    nexus interleaved =    no;
    delete Parvinatator Acamptonectes Maiaspondylus Arthropterygius Malawania Chacaicosaurus
Mollesaurus Leninia Thalattoarchon Palvennia Sisteronia;
        export file =    SI_D1_small.tnt.nex format =    nexus interleaved =    no;
    undelete Parvinatator Acamptonectes Maiaspondylus Arthropterygius Malawania
Chacaicosaurus Mollesaurus Leninia Thalattoarchon Palvennia Sisteronia;
        OUTGROUP 1;
    hsearch constraints = younginiform enforce = yes;
    savetrees file =    SI_D1.paup.tre replace =    Yes;
    contree /    file =    SI_D1.paup.con.tre replace =    Yes;
    gettrees file =    SI_D1.paup.tre duptrees =    Eliminate;
    describe 1 / LabelNode = No ChgList = No CMShowEq = No Plot = None;
END;
BEGIN ASSUMPTIONS;
    TYPESET * UNTITLED   =  unord:  1 -  195;
    EXSET * UNTITLED  = ;
END;
BEGIN NOTES;
    SUT   TAXA = Taxa TAXON = 26 NAME = color INTEGER = 14;
    SUT   TAXA = Taxa TAXON = 27 NAME = color INTEGER = 14;
    SUT   TAXA = Taxa TAXON = 28 NAME = color INTEGER = 14;
    SUT   TAXA = Taxa TAXON = 29 NAME = color INTEGER = 14;
    SUT   TAXA = Taxa TAXON = 30 NAME = color INTEGER = 5;
    SUT   TAXA = Taxa TAXON = 31 NAME = color INTEGER = 5;
    SUT   TAXA = Taxa TAXON = 32 NAME = color INTEGER = 5;
```

```
SUT   TAXA = Taxa TAXON = 33 NAME = color INTEGER = 5;
SUT   TAXA = Taxa TAXON = 34 NAME = color INTEGER = 5;
SUT   TAXA = Taxa TAXON = 35 NAME = color INTEGER = 5;
SUT   TAXA = Taxa TAXON = 36 NAME = color INTEGER = 5;
SUT   TAXA = Taxa TAXON = 46 NAME = color INTEGER = 11;
SUT   TAXA = Taxa TAXON = 47 NAME = color INTEGER = 11;
SUT   TAXA = Taxa TAXON = 48 NAME = color INTEGER = 11;
SUT   TAXA = Taxa TAXON = 49 NAME = color INTEGER = 11;
SUT   TAXA = Taxa TAXON = 50 NAME = color INTEGER = 11;
SUT   TAXA = Taxa TAXON = 61 NAME = color INTEGER = 16;
SU  T = 58 C = 2 N = color I = 5;
SU  T = 60 C = 5 N = color I = 5;
SU  T = 60 C = 6 N = color I = 5;
SU  T = 62 C = 6 N = color I = 5;
SU  T = 28 C = 7 N = color I = 17;
SU  T = 29 C = 7 N = color I = 2;
SU  T = 19 C = 8 N = color I = 5;
SU  T = 44 C = 12 N = color I = 5;
SU  T = 63 C = 13 N = color I = 5;
SU  T = 63 C = 14 N = color I = 5;
SU  T = 65 C = 14 N = color I = 5;
SU  T = 29 C = 15 N = color I = 17;
SU  T = 72 C = 15 N = color I = 5;
SU  T = 63 C = 16 N = color I = 5;
SU  T = 31 C = 19 N = color I = 5;
SU  T = 22 C = 21 N = color I = 5;
SU  T = 26 C = 23 N = color I = 5;
SU  T = 63 C = 23 N = color I = 5;
SU  T = 22 C = 26 N = color I = 5;
SU  T = 58 C = 27 N = color I = 5;
SU  T = 16 C = 29 N = color I = 5;
SU  T = 35 C = 29 N = color I = 5;
SU  T = 62 C = 30 N = color I = 17;
SU  T = 63 C = 30 N = color I = 5;
SU  T = 29 C = 31 N = color I = 5;
SU  T = 39 C = 31 N = color I = 5;
SU  T = 60 C = 31 N = color I = 5;
SU  T = 22 C = 32 N = color I = 5;
SU  T = 35 C = 32 N = color I = 5;
SU  T = 38 C = 33 N = color I = 5;
SU  T = 57 C = 33 N = color I = 5;
SU  T = 60 C = 36 N = color I = 5;
SU  T = 22 C = 38 N = color I = 5;
SU  T = 22 C = 39 N = color I = 5;
```

```
SU  T = 22 C = 40 N = color I = 5;
SU  T = 63 C = 41 N = color I = 5;
SU  T = 65 C = 43 N = color I = 5;
SU  T = 41 C = 44 N = color I = 5;
SU  T = 63 C = 46 N = color I = 5;
SU  T = 17 C = 49 N = color I = 5;
SU  T = 22 C = 54 N = color I = 5;
SU  T = 29 C = 55 N = color I = 2;
SU  T = 22 C = 56 N = color I = 4;
SU  T = 57 C = 59 N = color I = 5;
SU  T = 57 C = 60 N = color I = 5;
SU  T = 44 C = 61 N = color I = 5;
SU  T = 21 C = 62 N = color I = 5;
SU  T = 22 C = 62 N = color I = 5;
SU  T = 54 C = 62 N = color I = 5;
SU  T = 39 C = 63 N = color I = 5;
SU  T = 22 C = 66 N = color I = 5;
SU  T = 22 C = 68 N = color I = 5;
SU  T = 22 C = 70 N = color I = 5;
SU  T = 22 C = 71 N = color I = 5;
SU  T = 41 C = 77 N = color I = 5;
SU  T = 44 C = 77 N = color I = 7;
SU  T = 44 C = 78 N = color I = 7;
SU  T = 30 C = 79 N = color I = 5;
SU  T = 41 C = 79 N = color I = 5;
SU  T = 22 C = 81 N = color I = 5;
SU  T = 44 C = 81 N = color I = 7;
SU  T = 22 C = 82 N = color I = 5;
SU  T = 37 C = 82 N = color I = 5;
SU  T = 40 C = 82 N = color I = 5;
SU  T = 44 C = 82 N = color I = 7;
SU  T = 22 C = 83 N = color I = 5;
SU  T = 44 C = 83 N = color I = 7;
SU  T = 22 C = 84 N = color I = 5;
SU  T = 44 C = 84 N = color I = 7;
SU  T = 22 C = 85 N = color I = 5;
SU  T = 37 C = 85 N = color I = 5;
SU  T = 44 C = 85 N = color I = 7;
SU  T = 22 C = 86 N = color I = 5;
SU  T = 37 C = 86 N = color I = 5;
SU  T = 40 C = 86 N = color I = 5;
SU  T = 44 C = 86 N = color I = 7;
SU  T = 18 C = 87 N = color I = 5;
SU  T = 22 C = 87 N = color I = 5;
```

```
SU  T = 37 C = 87 N = color I = 5;
SU  T = 44 C = 87 N = color I = 7;
SU  T = 22 C = 88 N = color I = 5;
SU  T = 44 C = 88 N = color I = 7;
SU  T = 22 C = 89 N = color I = 5;
SU  T = 44 C = 89 N = color I = 7;
SU  T = 22 C = 90 N = color I = 5;
SU  T = 44 C = 90 N = color I = 7;
SU  T = 44 C = 91 N = color I = 7;
SU  T = 22 C = 92 N = color I = 5;
SU  T = 44 C = 92 N = color I = 7;
SU  T = 22 C = 93 N = color I = 5;
SU  T = 37 C = 93 N = color I = 5;
SU  T = 22 C = 94 N = color I = 5;
SU  T = 37 C = 94 N = color I = 5;
SU  T = 22 C = 95 N = color I = 5;
SU  T = 25 C = 95 N = color I = 5;
SU  T = 22 C = 96 N = color I = 5;
SU  T = 63 C = 97 N = color I = 5;
SU  T = 22 C = 98 N = color I = 5;
SU  T = 22 C = 99 N = color I = 5;
SU  T = 63 C = 99 N = color I = 5;
SU  T = 63 C = 100 N = color I = 5;
SU  T = 22 C = 101 N = color I = 5;
SU  T = 22 C = 102 N = color I = 5;
SU  T = 31 C = 102 N = color I = 5;
SU  T = 33 C = 102 N = color I = 5;
SU  T = 34 C = 102 N = color I = 5;
SU  T = 22 C = 103 N = color I = 5;
SU  T = 58 C = 103 N = color I = 5;
SU  T = 22 C = 104 N = color I = 5;
SU  T = 58 C = 104 N = color I = 5;
SU  T = 22 C = 105 N = color I = 5;
SU  T = 63 C = 105 N = color I = 5;
SU  T = 22 C = 106 N = color I = 5;
SU  T = 58 C = 106 N = color I = 5;
SU  T = 22 C = 107 N = color I = 5;
SU  T = 63 C = 107 N = color I = 5;
SU  T = 44 C = 108 N = color I = 5;
SU  T = 42 C = 111 N = color I = 5;
SU  T = 62 C = 112 N = color I = 17;
SU  T = 62 C = 113 N = color I = 17;
SU  T = 62 C = 114 N = color I = 17;
SU  T = 62 C = 115 N = color I = 17;
```

```
SU  T = 33 C = 120 N = color I = 5;
SU  T = 33 C = 121 N = color I = 4;
SU  T = 63 C = 122 N = color I = 5;
SU  T = 54 C = 123 N = color I = 5;
SU  T = 63 C = 123 N = color I = 5;
SU  T = 24 C = 128 N = color I = 4;
SU  T = 62 C = 128 N = color I = 17;
SU  T = 62 C = 129 N = color I = 17;
SU  T = 58 C = 130 N = color I = 5;
SU  T = 62 C = 130 N = color I = 17;
SU  T = 23 C = 131 N = color I = 4;
SU  T = 62 C = 131 N = color I = 17;
SU  C = 132 N = color I = 4;
SU  T = 1 C = 132 N = color I = 4;
SU  T = 2 C = 132 N = color I = 4;
SU  T = 3 C = 132 N = color I = 4;
SU  T = 4 C = 132 N = color I = 4;
SU  T = 5 C = 132 N = color I = 4;
SU  T = 6 C = 132 N = color I = 4;
SU  T = 7 C = 132 N = color I = 4;
SU  T = 8 C = 132 N = color I = 4;
SU  T = 9 C = 132 N = color I = 4;
SU  T = 10 C = 132 N = color I = 4;
SU  T = 11 C = 132 N = color I = 4;
SU  T = 12 C = 132 N = color I = 4;
SU  T = 13 C = 132 N = color I = 4;
SU  T = 14 C = 132 N = color I = 4;
SU  T = 15 C = 132 N = color I = 4;
SU  T = 16 C = 132 N = color I = 4;
SU  T = 17 C = 132 N = color I = 4;
SU  T = 18 C = 132 N = color I = 4;
SU  T = 19 C = 132 N = color I = 4;
SU  T = 20 C = 132 N = color I = 4;
SU  T = 21 C = 132 N = color I = 4;
SU  T = 22 C = 132 N = color I = 4;
SU  T = 23 C = 132 N = color I = 4;
SU  T = 25 C = 132 N = color I = 4;
SU  T = 26 C = 132 N = color I = 4;
SU  T = 27 C = 132 N = color I = 4;
SU  T = 28 C = 132 N = color I = 4;
SU  T = 29 C = 132 N = color I = 4;
SU  T = 30 C = 132 N = color I = 4;
SU  T = 31 C = 132 N = color I = 4;
SU  T = 32 C = 132 N = color I = 4;
```

```
SU  T = 33 C = 132 N = color I = 4;
SU  T = 34 C = 132 N = color I = 4;
SU  T = 35 C = 132 N = color I = 4;
SU  T = 36 C = 132 N = color I = 4;
SU  T = 37 C = 132 N = color I = 4;
SU  T = 38 C = 132 N = color I = 4;
SU  T = 39 C = 132 N = color I = 4;
SU  T = 40 C = 132 N = color I = 5;
SU  T = 41 C = 132 N = color I = 4;
SU  T = 42 C = 132 N = color I = 4;
SU  T = 43 C = 132 N = color I = 4;
SU  T = 44 C = 132 N = color I = 4;
SU  T = 45 C = 132 N = color I = 4;
SU  T = 46 C = 132 N = color I = 4;
SU  T = 47 C = 132 N = color I = 4;
SU  T = 48 C = 132 N = color I = 4;
SU  T = 49 C = 132 N = color I = 4;
SU  T = 50 C = 132 N = color I = 4;
SU  T = 51 C = 132 N = color I = 4;
SU  T = 52 C = 132 N = color I = 4;
SU  T = 53 C = 132 N = color I = 4;
SU  T = 54 C = 132 N = color I = 4;
SU  T = 55 C = 132 N = color I = 4;
SU  T = 56 C = 132 N = color I = 4;
SU  T = 57 C = 132 N = color I = 4;
SU  T = 58 C = 132 N = color I = 4;
SU  T = 59 C = 132 N = color I = 4;
SU  T = 60 C = 132 N = color I = 4;
SU  T = 61 C = 132 N = color I = 4;
SU  T = 62 C = 132 N = color I = 17;
SU  T = 63 C = 132 N = color I = 4;
SU  T = 64 C = 132 N = color I = 4;
SU  T = 65 C = 132 N = color I = 4;
SU  T = 66 C = 132 N = color I = 4;
SU  T = 67 C = 132 N = color I = 4;
SU  T = 68 C = 132 N = color I = 4;
SU  T = 69 C = 132 N = color I = 4;
SU  T = 70 C = 132 N = color I = 4;
SU  T = 71 C = 132 N = color I = 4;
SU  T = 72 C = 132 N = color I = 4;
SU  T = 73 C = 132 N = color I = 4;
SU  C = 133 N = color I = 4;
SU  T = 1 C = 133 N = color I = 4;
SU  T = 2 C = 133 N = color I = 4;
```

```
SU  T = 3 C = 133 N = color I = 4;
SU  T = 4 C = 133 N = color I = 4;
SU  T = 5 C = 133 N = color I = 4;
SU  T = 6 C = 133 N = color I = 4;
SU  T = 7 C = 133 N = color I = 4;
SU  T = 8 C = 133 N = color I = 4;
SU  T = 9 C = 133 N = color I = 4;
SU  T = 10 C = 133 N = color I = 4;
SU  T = 11 C = 133 N = color I = 4;
SU  T = 12 C = 133 N = color I = 4;
SU  T = 13 C = 133 N = color I = 4;
SU  T = 14 C = 133 N = color I = 4;
SU  T = 15 C = 133 N = color I = 4;
SU  T = 16 C = 133 N = color I = 4;
SU  T = 17 C = 133 N = color I = 4;
SU  T = 18 C = 133 N = color I = 4;
SU  T = 19 C = 133 N = color I = 4;
SU  T = 20 C = 133 N = color I = 4;
SU  T = 21 C = 133 N = color I = 4;
SU  T = 22 C = 133 N = color I = 4;
SU  T = 23 C = 133 N = color I = 4;
SU  T = 24 C = 133 N = color I = 4;
SU  T = 25 C = 133 N = color I = 4;
SU  T = 26 C = 133 N = color I = 4;
SU  T = 27 C = 133 N = color I = 4;
SU  T = 28 C = 133 N = color I = 4;
SU  T = 29 C = 133 N = color I = 4;
SU  T = 30 C = 133 N = color I = 4;
SU  T = 31 C = 133 N = color I = 4;
SU  T = 32 C = 133 N = color I = 4;
SU  T = 33 C = 133 N = color I = 4;
SU  T = 34 C = 133 N = color I = 4;
SU  T = 35 C = 133 N = color I = 4;
SU  T = 36 C = 133 N = color I = 4;
SU  T = 37 C = 133 N = color I = 4;
SU  T = 38 C = 133 N = color I = 4;
SU  T = 39 C = 133 N = color I = 4;
SU  T = 40 C = 133 N = color I = 4;
SU  T = 41 C = 133 N = color I = 4;
SU  T = 42 C = 133 N = color I = 4;
SU  T = 43 C = 133 N = color I = 4;
SU  T = 44 C = 133 N = color I = 4;
SU  T = 45 C = 133 N = color I = 4;
SU  T = 46 C = 133 N = color I = 4;
```

```
SU  T = 47 C = 133 N = color I = 4;
SU  T = 48 C = 133 N = color I = 4;
SU  T = 49 C = 133 N = color I = 4;
SU  T = 50 C = 133 N = color I = 4;
SU  T = 51 C = 133 N = color I = 4;
SU  T = 52 C = 133 N = color I = 4;
SU  T = 53 C = 133 N = color I = 4;
SU  T = 54 C = 133 N = color I = 4;
SU  T = 55 C = 133 N = color I = 4;
SU  T = 56 C = 133 N = color I = 4;
SU  T = 57 C = 133 N = color I = 4;
SU  T = 58 C = 133 N = color I = 4;
SU  T = 59 C = 133 N = color I = 4;
SU  T = 60 C = 133 N = color I = 4;
SU  T = 61 C = 133 N = color I = 4;
SU  T = 62 C = 133 N = color I = 4;
SU  T = 63 C = 133 N = color I = 4;
SU  T = 64 C = 133 N = color I = 4;
SU  T = 65 C = 133 N = color I = 4;
SU  T = 66 C = 133 N = color I = 4;
SU  T = 67 C = 133 N = color I = 4;
SU  T = 68 C = 133 N = color I = 4;
SU  T = 69 C = 133 N = color I = 4;
SU  T = 70 C = 133 N = color I = 4;
SU  T = 71 C = 133 N = color I = 4;
SU  T = 72 C = 133 N = color I = 4;
SU  T = 73 C = 133 N = color I = 4;
SU  T = 29 C = 136 N = color I = 5;
SU  T = 29 C = 137 N = color I = 5;
SU  T = 25 C = 139 N = color I = 5;
SU  T = 25 C = 140 N = color I = 5;
SU  T = 25 C = 141 N = color I = 5;
SU  T = 25 C = 142 N = color I = 5;
SU  T = 68 C = 142 N = color I = 5;
SU  T = 25 C = 143 N = color I = 5;
SU  T = 66 C = 143 N = color I = 5;
SU  T = 27 C = 144 N = color I = 5;
SU  T = 25 C = 147 N = color I = 5;
SU  T = 25 C = 149 N = color I = 5;
SU  T = 25 C = 150 N = color I = 5;
SU  T = 25 C = 151 N = color I = 5;
SU  T = 25 C = 152 N = color I = 5;
SU  T = 58 C = 152 N = color I = 5;
SU  T = 25 C = 153 N = color I = 5;
```

```
    SU  T = 25 C = 154 N = color I = 5;
    SU  T = 25 C = 155 N = color I = 5;
    SU  T = 25 C = 156 N = color I = 5;
    SU  T = 25 C = 157 N = color I = 5;
    SU  T = 25 C = 158 N = color I = 5;
    SU  T = 25 C = 159 N = color I = 5;
    SU  T = 70 C = 159 N = color I = 5;
    SU  T = 25 C = 160 N = color I = 5;
    SU  T = 25 C = 161 N = color I = 5;
    SU  T = 25 C = 162 N = color I = 5;
    SU  T = 44 C = 162 N = color I = 5;
    SU  T = 25 C = 163 N = color I = 5;
    SU  T = 25 C = 164 N = color I = 5;
    SU  T = 66 C = 164 N = color I = 5;
    SU  T = 62 C = 166 N = color I = 5;
    SU  T = 44 C = 167 N = color I = 5;
    SU  T = 65 C = 167 N = color I = 5;
    SU  T = 73 C = 170 N = color I = 5;
    SU  T = 22 C = 171 N = color I = 5;
    SU  T = 22 C = 172 N = color I = 5;
    SU  C = 173 N = selected B = TRUE;
    SU  T = 44 C = 173 N = color I = 5;
    SU  T = 62 C = 175 N = color I = 2;
    SU  T = 26 C = 179 N = color I = 4;
    SU  T = 62 C = 181 N = color I = 17;
    SU  T = 62 C = 182 N = color I = 17;
    SU  T = 56 C = 183 N = color I = 5;
    SU  T = 58 C = 185 N = color I = 5;
    SU  T = 21 C = 189 N = color I = 5;
    SU  T = 39 C = 191 N = color I = 5;
    SU  T = 65 C = 191 N = color I = 5;
    SU  T = 62 C = 195 N = color I = 17;
END;
BEGIN LABELS;
    CHARGROUPLABEL 01Cranium COLOR = (RGB 1 0.03529412 0.41960784) ;
    CHARGROUPLABEL 02Mandible COLOR = (RGB 0.80784314 0.03529412 1) ;
    CHARGROUPLABEL 03Dentition COLOR = (RGB 1 0.03529412 0.03529412) ;
    CHARGROUPLABEL 04PectoralGirdle COLOR = (RGB 0.03529412 0.03529412 1) ;
    CHARGROUPLABEL 05Forelimb COLOR = (RGB 0.03529412 0.61176471 1) ;
    CHARGROUPLABEL 06PelvicGirdle COLOR = (RGB 0.03529412 1 0.61176471) ;
    CHARGROUPLABEL '07Hind_limb' COLOR = (RGB 0.80784314 1 0.03529412) ;
    CHARGROUPLABEL 08Axial COLOR = (RGB 1 0.61176471 0.03529412) ;
    CHARGROUPLABEL 09Dermal COLOR = (RGB 1 0.41960784 0.03529412) ;
END;
```

```
BEGIN SETS;
    CHARPARTITION * UNTITLED  =  01Cranium :  1 -  60, 02Mandible :  61 -  62, 03Dentition :  63 -  74, 04PectoralGirdle :  75 -  92, 05Forelimb :  93 -  135, 06PelvicGirdle :  136 -  145, '07Hind_limb' :  146 -  164, 08Axial :  165 - 190, 09Dermal :  191 -  195;
END;
BEGIN MESQUITECHARMODELS;
    ProbModelSet * UNTITLED   =  'Mk1 (est.)':  1 -  195;
END;
Begin MESQUITE;
        MESQUITESCRIPTVERSION 2;
        TITLE AUTO;
        tell ProjectCoordinator;
        timeSaved 1460677565168;
        getEmployee #mesquite.minimal.ManageTaxa.ManageTaxa;
        tell It;
            setID 0 7121830078017352332;
            tell It;
                setSelected  2;
                setDefaultOrder  0 2 1 3 10 4 5 6 7 8 9 11 12 13 14 15 16 17 18 19 20 21 22 23 24 25 26 27 28 29 30 31 32 33 34 35 36 37 38 39 40 41 42 43 44 45 46 47 48 49 50 51 52 53 54 55 56 57 58 59 60 61 62 63 64 65 66 67 68 69 70 71 72;
                attachments ;
            endTell;
        endTell;
        getEmployee
#mesquite.charMatrices.ManageCharacters.ManageCharacters;
        tell It;
            setID 0 2516980770027965603;
            tell It;
                setSelected  173;
                setDefaultOrder  0 1 2 3 4 5 165 6 7 8 9 10 11 12 183 13 14 15 166 16 17 18 19 169 20 21 22 23 24 168 25 26 27 28 29 30 31 32 33 34 167 35 36 37 38 39 40 41 42 43 44 45 48 49 164 50 163 188 170 184 46 47 51 52 53 54 55 56 57 58 59 60 61 62 63 64 65 66 67 68 69 70 71 72 73 74 75 76 77 78 79 80 81 82 83 84 85 86 87 88 89 90 91 92 93 94 95 96 97 98 99 177 174 175 176 101 102 103 104 105 106 107 108 109 110 111 112 171 172 173 178 179 185 189 100 113 114 180 115 116 118 119 120 117 121 122 123 124 125 126 127 128 129 130 131 132 133 134 135 136 137 138 139 140 141 142 159 143 144 145 146 147 148 157 190 149 150 151 152 181 193 194 154 155 156 158 160 161 162 182 153 186 187 191 192;
                attachments ;
            endTell;
            mqVersion 304;
```

```
			checksumv 0 3 2246349582 null  getNumChars 195 numChars 195 getNumTaxa 73 numTaxa 73  short true  bits 2305843009213694031  states 79 sumSquaresStatesOnly  32109.0  sumSquares  -1.4757395258967641E20 longCompressibleToShort false usingShortMatrix true  NumFiles 1 NumMatrices 1;
			mqVersion;
		endTell;
		getWindow;
		tell It;
			suppress;
			setResourcesState false false 147;
			setPopoutState 400;
			setExplanationSize 0;
			setAnnotationSize 0;
			setFontIncAnnot 0;
			setFontIncExp 0;
			setSize 1583 1066;
			setLocation -2 0;
			setFont SanSerif;
			setFontSize 10;
			getToolPalette;
			tell It;
			endTell;
			desuppress;
		endTell;
		getEmployee  #mesquite.minimal.ManageTaxa.ManageTaxa;
		tell It;
			showTaxa  #7121830078017352332 #mesquite.lists.TaxonList.TaxonList;
			tell It;
				setTaxa #7121830078017352332;
				getWindow;
				tell It;
					newAssistant #mesquite.lists.TaxonListCurrPartition.TaxonListCurrPartition;
					setExplanationSize 30;
					setAnnotationSize 20;
					setFontIncAnnot 0;
					setFontIncExp 0;
					setSize 1436 994;
					setLocation -2 0;
					setFont SanSerif;
					setFontSize 10;
					getToolPalette;
```

```
                    tell It;
                        setTool
mesquite.lists.TaxonList.TaxonListWindow.arrow;
                    endTell;
                endTell;
                showWindow;
                getEmployee #mesquite.lists.ColorTaxon.ColorTaxon;
                tell It;
                    setColor Magenta;
                    removeColor off;
                endTell;
                getEmployee
#mesquite.lists.TaxonListAnnotPanel.TaxonListAnnotPanel;
                tell It;
                    togglePanel off;
                endTell;
            endTell;
        endTell;
        getEmployee
#mesquite.trees.BasicTreeWindowCoord.BasicTreeWindowCoord;
        tell It;
            makeTreeWindow                              #7121830078017352332
#mesquite.trees.BasicTreeWindowMaker.BasicTreeWindowMaker;
            tell It;
                suppressEPCResponse;
                setTreeSource  #mesquite.trees.StoredTrees.StoredTrees;
                tell It;
                    setTreeBlock 1;
                    toggleUseWeights off;
                endTell;
                setAssignedID 935.1455299445086.6002950344002218511;
                getTreeWindow;
                tell It;
                    setExplanationSize 30;
                    setAnnotationSize 20;
                    setFontIncAnnot 0;
                    setFontIncExp 0;
                    setSize 1436 994;
                    setLocation -2 0;
                    setFont SanSerif;
                    setFontSize 10;
                    getToolPalette;
                    tell It;
                    endTell;
```

```
                    getTreeDrawCoordinator
#mesquite.trees.BasicTreeDrawCoordinator.BasicTreeDrawCoordinator;
                tell It;
                    suppress;
                    setTreeDrawer
#mesquite.trees.SquareLineTree.SquareLineTree;
                    tell It;
                        setNodeLocs
#mesquite.trees.NodeLocsStandard.NodeLocsStandard;
                        tell It;
                            branchLengthsToggle on;
                            toggleScale on;
                            toggleBroadScale off;
                            toggleCenter on;
                            toggleEven on;
                            setFixedTaxonDistance 0;
                        endTell;
                        setEdgeWidth 4;
                        showEdgeLines on;
                        orientUp;
                    endTell;
                    setBackground White;
                    setBranchColor Black;
                    showNodeNumbers off;
                    showBranchColors on;
                    labelBranchLengths off;
                    centerBrLenLabels on;
                    showBrLensUnspecified on;
                    showBrLenLabelsOnTerminals on;
                    setBrLenLabelColor 0 0 255;
                    setNumBrLenDecimals 6;
                    desuppress;
                    getEmployee
#mesquite.trees.BasicDrawTaxonNames.BasicDrawTaxonNames;
                    tell It;
                        setFontSize 18;
                        setColor Black;
                        toggleColorPartition off;
                        toggleColorAssigned on;
                        toggleShadePartition off;
                        toggleShowFootnotes on;
                        toggleNodeLabels on;
                        toggleCenterNodeNames off;
                        toggleShowNames on;
```

```
                namesAngle ?;
            endTell;
        endTell;
            setTreeNumber 2;
            setTree
'(1:0.9234831,((4:14.25266,(3:4.514811,2:4.604786):9.601883):14.64353,(5:39
.59726,((6:13.72827,((7:5.538208,8:5.549427):3.485887,(9:2.844809,10:2.9442
56):6.168474):4.719481):18.44112,((11:10.20773,12:10.11602):17.16676,((13:7
.538427,14:7.575328,15:7.545774):16.04869,((16:9.983254,(17:4.725165,18:4.7
43107):5.316873):10.1417,((19:10.62683,(28:9.799832,29:11.93106,71:13.10332
,(26:2.945482,27:6.219088):6.882901):2.302205):6.399617,((21:6.4125,(20:2.5
27387,22:3.666646):2.741084):5.742898,(23:5.7483,(24:3.080747,25:3.162838):
2.687751):3.089472):9.654351,((30:8.401217,31:10.13796,32:14.84348,33:14.91
571,(34:5.759099,(35:1.471234,36:4.815767):4.789123):11.85078):4.804461,(41
:21.08138,((40:2.954525,(38:6.795291,39:4.032479):3.245529):4.171259,(37:9.
976843,(42:4.880057,(43:4.565558,((45:9.591676,((49:2.568422,50:17.25893):8
.29251,((46:2.388347,47:12.12656,48:8.176731):2.527138,(65:7.92546,(61:41.0
6722,64:3.984093):18.14757):15.128):3.241694):4.36457):5.161783,(44:28.1237
6,(51:3.342737,(52:7.83566,(54:47.58846,56:17.33788,57:62.77426,60:27.75169
,62:10.51983,68:31.23149,69:15.76705,70:22.66994,72:31.23018,(73:14.52449,(
66:11.12571,67:10.36175):2.724454):49.17971,(53:9.992952,59:7.407002,(55:25
.82897,58:14.50977,63:16.83062):18.65066):17.82318):12.00393):13.85847):6.7
45672):6.907801):8.20571):6.218167):10.13689):4.345229):2.755162):5.424514)
:8.588199):3.116697):3.441235):3.72404):4.877922):3.830148):10.19395):13.26
325);';
            setDrawingSizeMode 0;
            toggleLegendFloat on;
            scale 0;
            toggleTextOnTree off;
            togglePrintName on;
            showWindow;
        endTell;
        desuppressEPCResponse;
        getEmployee #mesquite.trees.ColorBranches.ColorBranches;
        tell It;
            setColor Red;
            removeColor off;
        endTell;
        getEmployee #mesquite.ornamental.BranchNotes.BranchNotes;
        tell It;
            setAlwaysOn off;
        endTell;
        getEmployee
#mesquite.ornamental.ColorTreeByPartition.ColorTreeByPartition;
```

```
                tell It;
                    colorByPartition off;
                endTell;
                getEmployee
#mesquite.ornamental.DrawTreeAssocDoubles.DrawTreeAssocDoubles;
                tell It;
                    setOn on;
                    setDigits 4;
                    writeAsPercentage off;
                    toggleCentred off;
                    toggleHorizontal on;
                    toggleWhiteEdges on;
                    setFontSize 10;
                    setOffset 0  0;
                endTell;
                getEmployee
#mesquite.ornamental.DrawTreeAssocStrings.DrawTreeAssocStrings;
                tell It;
                    setOn on;
                    toggleCentred on;
                    toggleHorizontal on;
                    setFontSize 10;
                    setOffset 0  0;
                endTell;
                getEmployee #mesquite.trees.TreeInfoValues.TreeInfoValues;
                tell It;
                    panelOpen false;
                endTell;
            endTell;
        endTell;
        getEmployee
#mesquite.charMatrices.BasicDataWindowCoord.BasicDataWindowCoord;
        tell It;
            showDataWindow                                #2516980770027965603
#mesquite.charMatrices.BasicDataWindowMaker.BasicDataWindowMaker;
            tell It;
                getWindow;
                tell It;
                    setExplanationSize 30;
                    setAnnotationSize 20;
                    setFontIncAnnot 0;
                    setFontIncExp 0;
                    setSize 1436 994;
                    setLocation -2 0;
```

```
                    setFont SanSerif;
                    setFontSize 10;
                    getToolPalette;
                    tell It;
                        setTool
mesquite.charMatrices.ColorCells.ColorCells.ColorCells;
                    endTell;
                    setTool
mesquite.charMatrices.ColorCells.ColorCells.ColorCells;
                    colorCells
#mesquite.charMatrices.ColorCells.ColorCells;
                tell It;
                    setColor Pink;
                    removeColor off;
                endTell;
                    colorRowNames
#mesquite.charMatrices.TaxonGroupColor.TaxonGroupColor;
                    colorColumnNames
#mesquite.charMatrices.CharGroupColor.CharGroupColor;
                    colorText  #mesquite.charMatrices.NoColor.NoColor;
                    setBackground White;
                    toggleShowNames off;
                    toggleShowTaxonNames on;
                    toggleTight off;
                    toggleThinRows off;
                    toggleShowChanges on;
                    toggleSeparateLines off;
                    toggleShowStates on;
                    toggleAutoWCharNames on;
                    toggleAutoTaxonNames off;
                    toggleShowDefaultCharNames off;
                    toggleConstrainCW on;
                    toggleBirdsEye off;
                    toggleShowPaleGrid off;
                    toggleShowPaleCellColors off;
                    toggleShowPaleExcluded off;
                    togglePaleInapplicable on;
                    toggleShowBoldCellText off;
                    toggleAllowAutosize on;
                    toggleColorsPanel off;
                    toggleDiagonal on;
                    setDiagonalHeight 80;
                    toggleLinkedScrolling on;
                    toggleScrollLinkedTables off;
```

```
                endTell;
                showWindow;
                getWindow;
                tell It;
                    forceAutosize;
                endTell;
                getEmployee
#mesquite.charMatrices.ColorByState.ColorByState;
                tell It;
                    setStateLimit 9;
                    toggleUniformMaximum on;
                endTell;
                getEmployee
#mesquite.categ.StateNamesEditor.StateNamesEditor;
                tell It;
                    makeWindow;
                    tell It;
                        setExplanationSize 30;
                        setAnnotationSize 20;
                        setFontIncAnnot 0;
                        setFontIncExp 0;
                        setSize 1436 994;
                        setLocation -2 0;
                        setFont SanSerif;
                        setFontSize 10;
                        getToolPalette;
                        tell It;
                        endTell;
                        rowsAreCharacters on;
                        toggleConstrainChar on;
                        toggleConstrainCharNum 3;
                        togglePanel off;
                        toggleSummaryPanel off;
                    endTell;
                    showWindow;
                endTell;
                getEmployee #mesquite.categ.StateNamesStrip.StateNamesStrip;
                tell It;
                    showStrip on;
                endTell;
                getEmployee #mesquite.charMatrices.AnnotPanel.AnnotPanel;
                tell It;
                    togglePanel off;
                endTell;
```

```
                getEmployee
#mesquite.charMatrices.CharReferenceStrip.CharReferenceStrip;
                tell It;
                    showStrip off;
                endTell;
                getEmployee
#mesquite.charMatrices.QuickKeySelector.QuickKeySelector;
                tell It;
                    autotabOff;
                endTell;
                getEmployee
#mesquite.charMatrices.SelSummaryStrip.SelSummaryStrip;
                tell It;
                    showStrip off;
                endTell;
                getEmployee
#mesquite.categ.SmallStateNamesEditor.SmallStateNamesEditor;
                tell It;
                    panelOpen true;
                endTell;
            endTell;
        endTell;
        getEmployee
#mesquite.charMatrices.ManageCharacters.ManageCharacters;
        tell It;
            showCharacters                                    #2516980770027965603
#mesquite.lists.CharacterList.CharacterList;
            tell It;
                setData 0;
                getWindow;
                tell It;
                    newAssistant
#mesquite.lists.DefaultCharOrder.DefaultCharOrder;
                    newAssistant
#mesquite.lists.CharListInclusion.CharListInclusion;
                    newAssistant
#mesquite.lists.CharListPartition.CharListPartition;
                    newAssistant
#mesquite.parsimony.CharListParsModels.CharListParsModels;
                    setExplanationSize 30;
                    setAnnotationSize 20;
                    setFontIncAnnot 0;
                    setFontIncExp 0;
                    setSize 1436 994;
```

```
                    setLocation -2 0;
                    setFont SanSerif;
                    setFontSize 10;
                    getToolPalette;
                    tell It;
                        setTool
mesquite.lists.CharacterList.CharacterListWindow.ibeam;
                    endTell;
                    setActive;
                endTell;
                showWindow;
                getEmployee
#mesquite.lists.CharListAnnotPanel.CharListAnnotPanel;
                tell It;
                    togglePanel off;
                endTell;
            endTell;
        endTell;
        endTell;
end;
```

Supplementary NEX File Data2 -- NEX file data for the phylogenetic hypotheses of the new taxon among Diapsida when aquatic adaptations are recoded as ambiguous

```
#NEXUS

Begin data;
	Dimensions ntax=43 nchar=218;
	Format datatype=standard symbols="0123" gap=-;
	Matrix

Seymouriidae
0000000000000000000000100??00000010?0000000?00000000000000000000000000000000000
000000000000000000?00000001010(01)11000000000000100000000(01)1000?0??00000000000
0010000000000000000000000000000000000000000000000000000000000?
Synapsida
(01)0000000(01)(01)0000000000000??000011(01)0?100(01)0000101000(01)0000000000000000
0000000000000000000000000(01)0000(01)0000000000(01)01(01)1000000100000000100000010?
0000?0??00000000000(01)0100000000000001000(01)0000000000000000000(02)00000(01)00000
00000000000000
Parareptilia
(01)00000(01)10000000(01)0000(01)1(01)??(01)0(01)010(01)0?(01)000000?(0 )(01)1000000000
0000000000(0 1)(0 1)(0 1)(0 1)000001(0
1)00(01)(01)0(01)(01)00002(01)010(01)1(01)(01)(01)00(0 1)0(0 1)00000010{0 1}000000(0
1)000000011000(0 1)000?(0 2)000?0??(012)(01)00000?000000(0 1)0000(0 1 2)0(1 2)000(0 1)(0
1)000(0 1)00(0 1)0010000000000000(0 1)00000(01)01000000000(1 2)000000{0 1}
Captorhinidae
0001000100000000000000??00001011?(01)000010?001000000?0000000100000000000001000
000001???0000?0100000000100000100000000000000?0100000000000001?000?00000100000
0000000200000000000000000000000000002000000000000000000000000
Araeoscelidia
1000000100000?0000000000001000101100000000(01)0100000010000000010?00?000??0001000
0100000100100001000000000000000000000000110000?0100000010000100 1?000000000101010
1000000(1 2)0000100000000000000000000100010000000000000300010000000
Rhynchocephalia 0(0 2)000010100001?(1 2)000001100(0 1)0001(0 1)011(0 1)(0 2)1011(0
1)1(1 2)010(0 1)1(0 1)0010(0 1)1(0 1)0111(0 1)111001(0
1)1100011010000100(01)2010101101011131(01)00000102(0 1)000(0 1)010(0
1)0100011000000100(0 1)(1 2)100?00011100(01)0010001000(01)20100001000(0
1)0110111100(0 1)1111(0 1)(0 1)00(0 1)00000111000011100(0 1)0100000
Squamata (0 1 2)0(0 1)000(0 1)0(0 1)00(0 1 2)010(1 2)0000(0 1)11000000(0 1)0(0
1)1(0 1)(0 1 2)(0 2)10110(1 2)2201??(0 1)(0 1)0(0 1)(0 1)(0 1)1001111111(0 1)01111(0 1)001(0
1)0011(0 1)010(0 1)01(0 1 2)101011(0 1)0(0 1)11(0 1)311000001(0 1)2(0 1)0001(0 2)1010(0
1)000110(0 1)00001000(1 2)101?0(0 2)01(0 1)10(0 1)10010101000(0 1)(0 2)11000(0 1)(0
1)010(0 1)011011(0 1)110111111(0 1)00100001(0 1)1100001110020100000
Prolacerta
```

1100000101000?010110000001100001100011110120001100010011011101110011?0001001000
000?0101011010100110100000010100001012010?0110010111?012?00??00110001100000000
1012200110101101011100101110100020000?02000003100101000?
Trilophosaurus
110000???0000???00111??1101000???1231111010??0????0?0?11111?01?110111000?100111?01
00??2101011100??101002?0?11210001(1
2)100100?01??0101110000100?0??110001?1?000100?10020?001000110101110010111010001000
000?020000011?110100?00
Rhynchosauria
0111101000000?0111110110100000111123111112100111000001111110011?0011100110010000
1010101011110111112110200110200001011010?0010010111001210?0?0110001110010100001
0110001100011010111101011101100300000112?0000110010100?00
Archosauriformes (0 1)1(0 1)(0 1)101(0 1)(0 1)1000(0 1)0101110000(0 1)(0 1)00001010(0
2)(0 2)1?(0 1)1011(1
2)0010(01)0010(01)1(01)111?011111?11?1(01)0110100001(01)1(01)1(012)1011(01)111001?1110
000(01)10(12)00001(012)10(01)(01)0(01)01010(01)1(02)112001(12)?00?0(12)011000110110010
00(0 1)0110001(0 1 2)01011010111101(0 1)11100(0 1)(0 1)02000001(0 1)2(0 1)00001(0 1)0(0
1)1(0 1)(1 2)00?00
Claudiosaurus
100000?110000?11010?0?0001?0011011001011022010011000?001000?10???10???00010010100
000120100001?10010010000001010000?0110000?0100010101000010000000110001000?0001000
1102000100100000101100000000000001010000010000010111011100000
Coelurosauravus
00?0001111000???000???10000001001 1?011110221?0010?0????1?1110?11????1?????????????
??????1?01??????31?0?00??????0??010110?00?0?0001000?01??1??00????????0?????0?00020?0
02?0?00???1010000000000010100100100?0000110010?00???
Kuehneosauridae
010000101000000111110?000001000100102010110222000113010100011111???001??1000110?001
0?00??0?0110100011?0100000000010?0010101100?111?1?0002000??00000011100000000100100?
02120?00????00?10211001??100?01?10000???0000011100?1000?00
Acerosodontosaurus
1???0??10????00?????0??00??0???0?1??????002001???????????0??0?????????????????????0???
?????11?????1?1????0??????0?00?00?0??0???0?0?01??????0????0?????????????00100?20?10?1
??????????????????????0?????????0???3????0??0???
Tangasaurus ??
??????????????0????????????????????????????????????0??????0?????0??1???2????????????????1??
0??0?100???????00????????????01???????0????01?????0???0??0????00
Youngina
100000000000000001000?00?001?0011011100??000110?000000100???0?10?1???????????10??00??
00100?01??????00??10000001010000000?0?0000?10??0?01001??????00????0?1010?01?00100?
?0??0??0001??1??????000?10?0000000(0 1)???1??03????0?0?000
Thadeosaurus ??
????????0?????????????????????????????????1?10????0?00?0??0??0?00?01001??000??0????0??01
0?01000120?2001000000?011100??0?0?1?1000???00?000000?10?100?0?00

Lanthanolania
10???0?00?000?01????0?0????001?1????????012???????0???????????????????????0??????0???
??????1?????????0????0?2??
????????????????????0??????????3??????0????
Orovenator
10?0000?10000000000010000?010001???0{02}101???1{12}?????0?0??????0?10?00?0????000100
000?000000?10??0?00001?01?000000000?0?00???
???????????????????????????0????000?????????3??????0????
Sophineta
000000101000?101000?11102000?01?11001?11022000010????????1111???????????01?0?10???
0000010??0???????310?0000???0?0?00?1??1?0010?0???01?????????????????????????????????
??????0?????????????000?????00????????010???????0??
Pamelina
010000101000??11110000000101?10?10201011022220?0113???????1111????????11?01?0??0???
00??0101?0?0?????010?00?0?010?0?10?0??0?0101?11??02??????????????????????????????????
?????????????????????0??1??00?????????11??????????
Tanystropheus
11000011?1000?01011?00100010010111101111012201??0001001101110111?1111?10010010000
000020101001010011010000001121000101211 0(012)?011001010 20000?00?0001100011000000
000101210012010111101110001111002000001001020000131001010 0000
Choristodera
2110000101010101 11(01)00?1100000010?(01)2111110110?(01)001001011101110111?0011?0??1
10010001000011010010?0??1011000001010000111010001 0(01)10011001000110 0?0011100010
1?00010001(0 2)1120010010(0 1)0?101110?1011101010100100100010001001101 00000
Macrocnemus
11000011?1000?01?11?0?1?01?00000102111110121 0?????0??0?1?1011?11?01?1110?1?0??0??0
0???0101001?1001101?000001011000101211 0??00100101?10011?0000001100011000000000 1?
122001?00011110111000011101000000100102000?131?010100000
Hovasaurus ?????????????????????0??00?101?0??1?0?0??002011??00?10????01???01
?0???1?????????????????2?????1???????????????0?0???00000?0??0???0?01?10002??0?000010??
???10001000120?2?010?10000?01?10000?0??101020???00000000?300?100?0010
Pachypleurosaurs
1000001000(01)00??2010?0??0010011111 1001(01)11022010111?0?10?1011101??1???11?100111
1?0111???0?010010?111?1100000010210001010111010100011002000 2?00?0011001211?0200(0
1)1101(0 1)01(1 2)00120100011011000011000001 1(0 1)10???(0 1)?????0???????1000?0
Simosaurus
20000010101 00??2010?0??0000001101111101102201 0111?01001100110 1??1???10?1000111?01
1000211011010?11??110001101121100111011101010?111102000??00?0011001210002 00111011
112001201?0011?11000011000 0211?10?????????0?2??????000?0
Placodus
001001110101 0??2010?11?001010101111011110020 10?01?010010011101??1?1?1101102001101
10?010011011110001111011011021010001011010110011010200 01?00?11111011100000011101
0112001201000110111000110000(0 2)11220????????0??2??0??100000
Pistosauridae

10?0001010100??2011?0??01100011011131011012011??1?0?0111001101??1???11?1001011?01
10?0?1?0???10?11??1000011011210001110??1010100?1?102(01)0???(01)0?0(01)11101?1??020
0?11011112011001010(0 1)?01?000????001??0(0 1 2)1???????????2??????00000
Askeptosaurus
100001110010000100100?001010101010001111012101??0101?11101111?11?01?1?0001?0?0100
00112110001100111?01000000112100010101101101000101010?000000?0010000100000000001
0121011001000?101110000111000102?1??0?0?00000??????100000
Clarazia
0011001100100???001?0????11011100?00?1110122?1??010??111?1111???????1?000110?010??0
?1?0111011?000??311000011101110?0?0110?0010?0?01010?01?000???110?0100000000?0????2
001?00?00??11110?01110000110?0???????000?0?????100000
Thalattosaurus
1011001100100???011?01?0?110?1100?00?111012201??010??111?1111???????1???01?0?01???0
?1?111101100011121100001?12011?1010?00?10???00??01????????????????????100?00????????
1?????????01??????????01???1??????0?00???????1000??
Helveticosaurus
00?1001?1?????0?000?0?1?0????1??11???0??
0?0?001??000?00??0000?????00?0?01101?101001?10200?1?00?10011000100000010001?11100
1?010000100110??111000?11320?0????0???0?????0??0000
Largocephalosaurus
00?0001?00000??201110?100110001011310111020210?1?10??0???0010????????1?0?1?21?100?
0???100000??000??01?0(01)0?0??2??01101?1110?101000(01)??2(23)2?00100011110121000200
1?101(0 2)1210110010(0 1)00?0011??0(0 1)100??(0 2)10100??????????????????0000?0
Sinosaurosphargis
00?0001000000???01110?1001110?00??300111020210?1??0??0???010??????1??00???210100?
0002110000???0???01?00010?121001?00?1??2?20????0??202???10102111012???????1?10????1
0110000??????????????????2??100??????????????????100???
Wumengosaurus
10?0011100001?0100110?00010?(01)010?1???0110221?0111??????1?01?0???????????????????0?
?????0?01001?0000??1?10000?????00101?110??0100001?020?00?0000001011010002000?0013
0220010010100?0110??0??0000?11311?????????0????????00000
Nanchangosaurus
10?0?01?0?000?0100110?00011110011110101102210011001?00???00?0????????????????1??????
???0??100???0??????02?????????0?1?1??10?00100000002010111012121100?1??0000?00113122
?11?1?00???001??????????01020????????????????????01000
Hupehsuchus
10?0?01101000?0100111?00011110011110001102210011001??0???00?0?????????????0??11?0??1
???0?0100???0?0????02????12100?101?110?00100000002010111012121100?1??0000?00113122
1111110001?001001001000001020??????????????????????010{0 1}0
Chaohusaurus
10?0011101001?0100111?00112000010100101102210011001??0???0110?????????0??0?01?100?
1???0?00001??001?01?100001121010100?110?0210100(01)101000010000021000210?00000001
13122111(0 1)011000?101000011100001(0 1)001??????????????????????00100
Utatsusaurus

10?0011101001?0101110?0?112100011110111102010011001??0???001?????????????0?01010??
1???1100001?0011?01?10000111100010 1?110??2101?010010000?000002110001000000000113
122?11(0 1)011000?101001001100001000 1???????0???1??????00100
Cartorhynchus
00?0011101001?0100010?00002(01)0010010011110221001100 1??0?1?01?0???????????????????
??????0?00001??000????12?????????0?100?110?0210??0?101000011003021100010000000 00113
122111?0?10?0?001??????????01?010??????????????????10101
Sclerocormus
00??011??1001?0100?11?100001001001000111{01}200??0?00????????1????????????????????
??????1???0???????????12?????????0???0??1???2100?0??0100?0?10131211???1???0???0?1?????
????0?100??001??????????01?000???????????1??????11111
;
End;

begin PAUP;
log file = SI_D2.txt replace = Yes start;
SET AUTOCLOSE = YES;
SET INCREASE = AUTO;
SET CRITERION = PARSIMONY;
SET WARNTREE = NO;
SET WARNTSAVE = NO;
SET WARNROOT = NO;
SET WARNREDEF = NO;
SET WARNRESET = NO;
assume ancstates = standard;
ctype unord : all;
DEFAULTS CONTREE / STRICT MAJRULE LE50 INDICES Outroot = paraphyl File = consense.tr;
DEFAULTS HSEARCH ADDSEQ = RANDOM NREPS = 100 SWAP = TBR HOLD = 50 ENFORCE = NO;
DEFAULTS BANDB ADDSEQ = FURTHEST ENFORCE = NO;
DEFAULTS BOOTSTRAP NREPS = 1000 CONLEVEL = 50 METHOD = HEURISTIC;
DEFAULTS DESCRIBE ChgList = YES;
SET INCREASE = AUTO;
export file = SI_D2.tnt.nex format = nexus interleaved = no;
OUTGROUP 1;
hsearch;
describe 1 / LabelNode=No ChgList=No CMShowEq=No Plot=None;
savetrees file = SI_D2.tre replace = Yes;
contree / file = SI_D2Con.tre replace = Yes;
describe 1 / LabelNode=No ChgList=No CMShowEq=No Plot=None;
gettrees file = SI_D2.tre duptrees = Eliminate;
describe 1 / LabelNode=No ChgList=No CMShowEq=No Plot=None;
END;

Supplementary NEX File Data3 -- NEX file data for the phylogenetic hypotheses of the new taxon among Diapsida when aquatic adaptations are recoded normally

#NEXUS

Begin data;
Dimensions ntax=43 nchar=218;
Format datatype=standard symbols="0123" gap=-;
Matrix

Seymouriidae
000000000000000000000100??00000010?0000000?000000000000000000000000000000000000
000000000000000000?00000001010(01)11000000000000100000000(01)1000?0??00000000000
00100000000000000000000000000000000000000000000000000000000000?
Synapsida
(01)0000000(01)(01)0000000000000??000011(01)0?100(01)0000101000(01)0000000000000000
000000000000000000000000(01)0000(01)0000000000(01)01(01)1000000100000001000000l0?
0000?0??00000000000(01)0100000000000001000(01)0000000000000000000(02)00000(01)00000
00000000000000
Parareptilia
(01)00000(01)10000000(01)0000(01)1(01)??(01)0(01)010(01)0?(01)000000?(0)(01)1000000000
0000000000(0 1)(0 1)(0 1)(0 1)000001(0
1)00(01)(01)0(01)(01)00002(01)010(01)1(01)(01)(01)00(0 1)0(0 1)00000010{0 1}000000(0
1)000000011000(0 1)000?(0 2)000?0??(012)(01)00000?000000(0 1)0000(0 1 2)0(1 2)000(0 1)(0
1)000(0 1)00(0 1)0010000000000000(0 1)00000(01)01000000000(1 2)000000{0 1}
Captorhinidae
0001000100000000000000??00001011?(01)000010?001000000?0000000100000000000001000
000001???0000?010000000010000010000000000000?0100000000000001?000?00000100000
00000002000000000000000000000000000002000000000000000000000000
Araeoscelidia
1000000100000?000000000001000101100000000(01)0100000010000000010?00?000??0001000
0100000100100001000000000000000000000000110000?010000001000010001?000000000101010
1000000(1 2)000010000000000000000000100010000000000000300010000000
Rhynchocephalia 0(0 2)000010100001?(1 2)000001100(0 1)0001(0 1)011(0 1)(0 2)1011(0
1)1(1 2)010(0 1)1(0 1)0010(0 1)1(0 1)0111(0 1)111001(0
1)1100011010000100(01)2010101101011131(01)00000102(0 1)000(0 1)010(0
1)0100011000000100(0 1)(1 2)100?00011100(01)0010001000(01)20100001000(0
1)0110111100(0 1)1111(0 1)(0 1)00(0 1)000001110000111100(0 1)0100000
Squamata (0 1 2)0(0 1)000(0 1)0(0 1)00(0 1 2)010(1 2)0000(0 1)11000000(0 1)0(0
1)1(0 1)(0 1 2)(0 2)10110(1 2)2201??(0 1)(0 1)0(0 1)(0 1)(0 1)1001111111(0 1)01111(0 1)001(0
1)0011(0 1)010(0 1)01(0 1 2)101011(0 1)0(0 1)11(0 1)311000001(0 1)2(0 1)0001(0 2)1010(0
1)000110(0 1)00001000(1 2)101?0(0 2)01(0 1)10(0 1)10010101000(0 1)(0 2)11000(0 1)(0
1)010(0 1)011011(0 1)110111111(0 1)00100001(0 1)1100001110020100000
Prolacerta

1100000101000?0101100000011000011000111101200011000100110111011100111?0001001000
000?01010110101001101000000101000010120100?0110010111?012?00??001100011000000000
10122001101011010111001011101000200000?020000031001010000?
Trilophosaurus
110000???0000???00111??1101000???1231111010??0????0?0?11111?01?110111000?100111?01
00??2101011100??101002?0?11210001(1
2)100100?01??0101110000100?0??110001?1?000100?10020?00100011010111001011101000100
000?020000011?110100?00
Rhynchosauria
0111101000000?01111101101000001111231111112100111000001111110011?0011100110010000
10101010111101111121102001102000010110100?00100101110012100?0?011000111001010001
0110001100011010111101011101100300000112?0000110010100?00
Archosauriformes (0 1)1(0 1)(0 1)101(0 1)(0 1)1000(0 1)0101110000(0 1)(0 1)00001010(0
2)(0 2)1?(0 1)1011(1
2)0010(01)0010(01)1(01)111?011111?11?1(01)0110100001(01)1(01)1(012)1011(01)111001?1110
000(01)10(12)00001(012)10(01)(01)0(01)01010(01)1(02)112001(12)?00?0(12)011000110110010
00(0 1)0110001(0 1 2)01011010111101(0 1)11100(0 1)(0 1)02000001(0 1)2(0 1)00001(0 1)0(0
1)1(0 1)(1 2)00?00
Claudiosaurus
100000?110000?11010?0?0001?001101100101102201001100?001000?10???10???00010010100
000120100001?10010010000001010000?0110000?01000101010000100000011000100?0001000
1102000100100001011000000000001010000010000010111011100000
Coelurosauravus
00?0001111000???000???100000010011?011110221?0010?0????1?1110?11????1?????????????
??????1?01??????31?0?00??????0??010110?00?0?0001000?01??1??00????????0?????0?00020?0
02?0?00???1010000000000010100100100?0000110010?00???
Kuehneosauridae
0100001010000011110?00000100010010201011022200011301010001111???001??1000110?001
0?00??0?0110100011?010000000010?0010101100?111?1?0002000??000000111000000100100?
02120?00????00?10211001??100?01?10000???0000011100?1000?00
Acerosodontosaurus
1???0??10????00?????0??00??0???0?1??????002001???????????0??0?????????????????????0???
?????11?????1?1????0??????0?00?00?0??0???0?0?01??????0????0?????????????00100?20?10?1
?????????????????????0?????????0???3????0??0???
Tangasaurus ??
??????????????0?????????????????????????????????0??????0?????0??1???2????????????????1??
0??0?100???????00???????????01???????0????01?????0???0??0????00
Youngina
1000000000000001000?00?001?001101100??000110?000000100???0?10?1???????????10??00??
00100?01??????00??10000001010000000?0?0000?10??0?01001??????00????0?1010?01?00100?
?0??0??0001??1??????000?10?0000000(0 1)???1??03????0?0?000
Thadeosaurus ??
????????0???????????????????????????????1?10????0?00?0??0??0?00?01001??000??0????0??01
0?01000120?20010000000?011100??0?0?1?1000???00?000000?10?100?0?00

Lanthanolania
10???0?00?000?01????0?0????001?1????????012???????0????????????????????????0??????0???
??????1?????????0????0?2??
????????????????????0??????????3??????0????

Orovenator
10?0000?1000000000010000?010001???0{02}101???1{12}?????0?0??????0?10?00?0????000100
000?000000?10??0?00001?01?000000000?0?00???
????????????????????????????0????000?????????3??????0????

Sophineta
000000101000?101000?11102000?01?11001?11022000010????????1111???????????01?0?10???
0000010??0???????310?0000???0?0?00?1??1?0010?0???01??????????????????????????????????
??????0?????????????000?????00????????010???????0??

Pamelina
010000101000??111100000000101?10?1020101102220?0113???????1111????????11?01?0??0???
00??0101?0?0?????010?00?0?010?0?10?0??0?0101?11??02?????????????????????????????????
?????????????????????0??1??00?????????11??????????

Tanystropheus
11000011?1000?01011?001000100101111011110122O1??0001001101110111?1111?10010010000
00002010100101001101000000112100010121100(012)?01100101020000?00?0001100011000000
000101210012010111101110001111002000001001020000131001010000O

Choristodera
21100001010101010111(01)00?1100000010?(01)2111110110?(01)0010010111011101111?0011?0??1
10010001000011010010?0??101100000101000011101000010(01)10011001000110O?0011100010
1?00010001(0 2)1120010010(0 1)0?101110?101110101010010010001000100110100000

Macrocnemus
11000011?1000?01?11?0?1?01?00000102111101210?????0??0?1?1011?11?01?1110?1?0??0??0
0???0101001?1001101?000001011000101211O??00100101?10011?000000110001100000000001?
122001?0001111011100001110100000001001O2000?131?010100000

Hovasaurus ????????????????????0??00?101?0??1?0?0??002011??00?10????01???01
?0???1?????????????????2?????1???????????????0?0???00000?0??0???0?01?10002??0?000010??
???10001000120?2?010?10000?01?10000?0??101020???00000000?300?100?0010

Pachypleurosaurs
1000001000(01)00??2010?0??00100111111001(01)11022010111?0?10?1011101??1???11?100111
1?0111???0?010010?111?11000000102100010101110101000110020002?00?0011001211?0200(0
1)1101(0 1)01(1 2)001201000110110000110000011(0 1)10???(0 1)?????0???????1000?0

Simosaurus
2000001010100??2010?0??0000001101111101102201011?1?01001100110l??1???10?1000111?01
100021101101O?11??1100011011211001110111O1010?111102000??00?0011001210002001110111
112001201?0011?11000011000021 1?10?????????0?2??????000?0

Placodus
0010011101010??2010?11?0010101011110111100201 0?01?0100100111O1??1?1?1101102001101
10?010011011110001111011011021010001011010110011010 20001?00?1111101110000001 1101
0112001201000110111000110000(0 2)11220????????0??2??0??100000

Pistosauridae

10?0001010100??2011?0??0110001101113101101201 1??1?0?0111001101??1???11?1001011?01
10?0?1?0???10?11??10000110112100011 10??1010100?1?102(01)0???(01)0?0(01)11101?1??020
0?11011112011001010(0 1)?01?000?????001??0(0 1 2)1???????????2??????00000
Askeptosaurus
10000111001000010010 0?00101010101000111101210 1??0101?11101111?11?01?1?0001?0?0100
00112110001100111?010000001121000101011011010001010 10?000000?0010000100000000001
012101100100 0?1011100001110001 02?1??0?0?00000??????100000
Clarazia
00110011001 00???001?0????11011100?00?1110122?1??010??111?1111???????1?000110?010??0
?1?0111011?000??31100001110 1110?0?0110?0010?0?01010?01?000???110?010000000 0?0????2
001?00?00??11110?01110000110?0???????000?0?????100000
Thalattosaurus
10110011001 00???011?01?0?110?1100?00?111012201??010??111?1111???????1???01?0?01???0
?1?1111011000111211000 01?12011?1010?00?10???00??01???????????????????100?00????????
1?????????01??????????01???1??????0?00???????1000??
Helveticosaurus
00?1001?1?????0?000?0?1?0????1??11???0???
0?0?001??000?00??0000?????00?0?01101?101001?10200?1?00?100110001000000010001?11100
1?010000100110??111000?11320?0????0???0?????0??0000
Largocephalosaurus
00?0001?00000??201110?100110001011310111020210?1?10??0???0010????????1?0?1?21?100?
0???100000??000??01?0(01)0?0??2??01101?1110?101000(01)??2(23)2?001000111101210 00200
1?101(0 2)1210110010(0 1)00?0011??0(0 1)100??(0 2)10100?????????????????0000?0
Sinosaurosphargis
00?0001000000???01110?1001110?00??300111020210?1??0??0????010??????1??00???210100?
0002110000???0???01?00010?121001?00?1??2?20????0??202???10102111012???????1?10????1
0110000?????????????????2??100?????????????????100???
Wumengosaurus
10?0011100001?0100110?00010?(01)010?1???0110221?0111??????1?01?0??????????????????0?
?????0?01001?0000??1?10000?????00101?110??0100001?020?00?000000101101000 2000?0013
0220010010100?0110??0??0000?11311?????????0????????00000
Nanchangosaurus
10?0?01?0?000?0100110?0001111001111010110221001 1001?00???00?0???????????????1??????
???0??100???0??????02?????????0?1?1??10?001000000020101110121211 00?1??0000?00113122
?11?1?00???001??????????01020???????????????????01000
Hupehsuchus
10?0?01101000?0100111?0001111001111000110221001 1001??0???00?0?????????????0??11?0??1
???0?0100???0?0????02????12100?101?110?0010000000201011101212110 0?1??0000?00113122
1111110001?00100100100000 1020????????????????????010{0 1}0
Chaohusaurus
10?0011101001?0100111?001120000101001011022100 11001??0???0110?????????0??0?01?100?
1???0?00001??001?01?1000011210101 00?110?0210100(01)10100001000000210 00210?00000001
13122111(0 1)011000?101000011100001(0 1)001??????????????????00100
Utatsusaurus

10?0011101001?0101110?0?1121000111101111020100110 01??0???001?????????????0?01010??
1???1100001?0011?01?10000111000101?110??2101?010010000?000002110001000000000113
122?11(0 1)011000?1010010011000010001???????0???1??????00100
Cartorhynchus
00?0011101001?0100010?00002(01)0010010011110221001100 1??0?1?01?0???????????????????
??????0?00001??000????12?????????0?100?110?0210??0?1010000110030211000100000000113
122111?0?10?0?001??????????01?010??????????????????10101
Sclerocormus
00??011??1001?0100?11?1000010010010001 11{01}200??0?00????????1?????????????????????
??????1???0???????????12?????????0???0??1???2100?0??0100?0?10131211???1???0???0?1?????
????0?100??001??????????01?000???????????1??????11111 ;
End;

begin PAUP;
log file =\tSI_D3.txt replace =\tYes start;
SET AUTOCLOSE =\tYES;
SET INCREASE =\tAUTO;
SET CRITERION =\tPARSIMONY;
SET WARNTREE =\tNO;
SET WARNTSAVE =\tNO;
SET WARNROOT = NO;
SET WARNREDEF = NO;
SET WARNRESET = NO;
assume ancstates =\tstandard;
ctype unord :\tall;
DEFAULTS CONTREE /\tSTRICT MAJRULE LE50 INDICES Outroot =\tparaphyl File =\tconsense.tr;
DEFAULTS HSEARCH ADDSEQ =\tRANDOM NREPS =\t100 SWAP =\tTBR HOLD =\t50 ENFORCE =\tNO;
DEFAULTS BANDB ADDSEQ =\tFURTHEST ENFORCE =\tNO;
DEFAULTS BOOTSTRAP NREPS =\t1000 CONLEVEL =\t50 METHOD =\tHEURISTIC;
DEFAULTS DESCRIBE ChgList =\tYES;
SET INCREASE =\tAUTO;
export file =\tSI_D3.tnt.nex format =\tnexus interleaved =\tno;
OUTGROUP 1;
hsearch;
savetrees file =\tSI_D3.tre replace =\tYes;
contree /\tfile =\tSI_D3Con.tre replace =\tYes;
gettrees file =\tSI_D3.tre duptrees =\tEliminate;
describe 1 / LabelNode=No ChgList=No CMShowEq=No Plot=None;
END;
